# Supplementary material for: Area law of noncritical ground states in 1D long-range interacting systems
Source: Nat Commun. 2020 Sep 8;11:4478. doi: 10.1038/s41467-020-18055-x (PMC7479120; doi:10.1038/s41467-020-18055-x)
Supplement: Supplementary file 1 — Supplementary Information [file 41467_2020_18055_MOESM1_ESM.pdf]

Supplementary Information for
"Area law of non-critical ground states in 1D long-range interacting systems"

Tomotaka Kuwahara[1,2,3*]
[1] *Mathematical Science Team, RIKEN Center for Advanced Intelligence Project (AIP), 1-4-1 Nihonbashi, Chuo-ku, Tokyo 103-0027, Japan* and
[2] *Interdisciplinary Theoretical & Mathematical Sciences Program (iTHEMS) RIKEN 2-1, Hirosawa, Wako, Saitama 351-0198, Japan*

Keiji Saito[3†]
[3] *Department of Physics, Keio University, Yokohama 223-8522, Japan*

CONTENTS

Supplementary Note 1. Outline of area law proof 2
A. Set up and assumption 2
1. Definition of the Hamiltonian 2
2. Main assumption 3
3. Schmidt rank 4
B. Main results 4
C. Specific values of $\bar{\alpha}$ and g_0 5
1. Proof of Lemma 1 6
2. Proof of Lemma 2 6
D. Outline of the proof 7
1. Preliminaries 7
2. Brief outline 8

Supplementary Note 2. Details of technical lemmas, propositions and sub-theorems 10
A. Gap condition for the truncated Hamiltonian $H_{\rm t}$ 10
B. Perturbation of the ground state 11
C. Convenient lemmas on the Schmidt rank 12
D. The Eckart-Young theorem 13
E. Overlap between the ground state and low-entangled state 14
1. Proof of Lemma 7 15
F. Upper bound of the entanglement entropy by the AGSP operators 16
G. Schmidt rank of the polynomials of the truncated Hamiltonian 18
1. Proof of Lemma 9 19
2. Proof of Lemma 10 20
H. Construction of the AGSP 21
I. Effective Hamiltonian with a small norm 22
1. Proof of Lemma 12 23

Supplementary Note 3. Proof of Main Theorem 1 24
A. Proof of Proposition 6 25
B. Proof of Proposition 7 26

Supplementary Note 4. Proof of Theorem 5: Accuracy of the effective Hamiltonian with multi-energy cut-off 28
A. Preliminaries 29
1. Upper bound of the spectral gap for $\tilde{H}_{\rm t}$ 29
2. Lower bound of the spectral gap $\tilde{\Delta}_{\rm t}$ for $\tilde{H}_{\rm t}$ 30
3. Lower bound of $\langle\psi|\tilde{H}_{\rm t}|\psi\rangle$ 31
4. Upper bound of the norm difference $\||0_{\rm t}\rangle - |\tilde{0}_{\rm t}\rangle\|$ 32
B. Outline of the proof 33
C. Proof of Proposition 9 by utilizing Proposition 8 35
D. Proof of Proposition 8: the first part (278) 35
1. Proof of Lemma 17 37
E. Proof of Proposition 8: the second part (280) 38

* tomotaka.kuwahara@riken.jp
† saitoh@rk.phys.keio.ac.jp

F. Proof of Proposition 10 40
1. Proof of the inequality (358) 41

Supplementary Note 5. List of notations and definitions 42

Supplementary References 43

Supplementary Note 1. OUTLINE OF AREA LAW PROOF

A. Set up and assumption

We here restate the setup of the system. We consider a one-dimensional quantum system with n sites, where each of the sites has d-dimensional Hilbert space. We denote the total set of the sites by Λ, namely $\Lambda = \{1, 2, \ldots, n\}$.

In Supplementary Table I, we give a list of parameters which are used throughout the proof. In Supplementary Note 5, we give a list of definitions and notations which we use several times in the proof.

1. *Definition of the Hamiltonian*

We define the system Hamiltonian H as

$$H = \sum_{|Z| \le k} h_Z \tag{1}$$

with $|Z|$ the cardinality of Z, where each of $\{h_Z\}_{|Z|\le k}$ denotes an interaction between the sites in $Z \subset \Lambda$. For example, in the case of $k = 2$, the Hamiltonian is given in the form of

$$H = \sum_{Z:|Z|=2} h_Z + \sum_{Z:|Z|=1} h_Z = \sum_{i<j} h_{i,j} + \sum_{i=1}^{n} h_i. \tag{2}$$

This is the Hamiltonian that we considered in the main paper.

The Hamiltonian (1) describes a generic k-body-interacting system. We assume the power-law decaying interaction as

$$\max_{i\in\Lambda} \sum_{Z:Z\ni i,\mathrm{diam}(Z)=r} \|h_Z\| \le \frac{J}{r^\alpha} \quad (\alpha > 1), \tag{3}$$

and

$$\max_{i\in\Lambda} \|h_{\{i\}}\| \le B, \tag{4}$$

where $\mathrm{diam}(Z) = \max_{i,j\in Z}(|i-j|)$ and $\|\cdots\|$ is the operator norm. In the proof, we often use the notation of $\sum_{Z:\mathrm{condition}}$ which means the summation over all Z satisfying the condition. Thus, $\sum_{Z:Z\ni i,\mathrm{diam}(Z)=r}$ means the summation which picks up all the subsets $Z \subset \Lambda$ such that $Z \ni i$ and $\mathrm{diam}(Z) = r$. From Ineqs. (3) and (4), we immediately obtain

$$\max_{i\in\Lambda} \sum_{Z:Z\ni i} \|h_Z\| = B + \max_{i\in\Lambda} \sum_{r=1}^{\infty} \sum_{Z:Z\ni i,\mathrm{diam}(Z)=r} \|h_Z\| \le B + \frac{\alpha J}{\alpha - 1} =: g, \tag{5}$$

where in the last inequality we use

$$\sum_{r=1}^{\infty} \sum_{Z:Z\ni i,\mathrm{diam}(Z)=r} \|h_Z\| \le J \sum_{r=1}^{\infty} r^{-\alpha} \le J + J\int_1^\infty x^{-\alpha}dx = \frac{\alpha J}{\alpha - 1}. \tag{6}$$

We assume a non-degenerate ground state $|0\rangle$ with a spectral gap Δ. We notice that the spectral gap is always smaller than $2g$ (see below for the derivation):

$$0 < \Delta \le 2g. \tag{7}$$

Throughout the paper, by appropriately choosing the energy unit, we set $g = 1$, or equivalently $B + \alpha J/(\alpha - 1) = 1$.

Proof of the inequality (7). We here would like to prove

$$\Delta \le 2 \max_{i\in\Lambda} \sum_{Z:Z\ni i} \|h_Z\| = 2g, \tag{8}$$

where we use the definition of g in (5). It has been already given in Ref. [1], but we show the proof here. Let us decompose the Hamiltonian as $H = H_{\Lambda_i} + V_i$, where H_{Λ_i} acts only on the sites $\Lambda_i := \Lambda \setminus \{i\}$ and $V_i := H - H_{\Lambda_i}$. We note that $\|V_i\| \le g$ from the inequality (5). we consider a quantum state $|\phi\rangle = |\phi_i\rangle \otimes |0_{\Lambda_i}\rangle$ with $|0_{\Lambda_i}\rangle$ the ground state of H_{Λ_i}. By choosing $|\phi_i\rangle$ such that $\langle 0|\phi\rangle = 0$, we have

$$\langle\phi|H|\phi\rangle - \langle 0|H|0\rangle \ge \Delta. \tag{9}$$

On the other hand, we have

$$\langle\phi|H|\phi\rangle \le \langle 0_{\Lambda_i}|H_{\Lambda_i}|0_{\Lambda_i}\rangle + \|V_i\| \le \langle 0_{\Lambda_i}|H_{\Lambda_i}|0_{\Lambda_i}\rangle + g \tag{10}$$

and

$$\langle 0|H|0\rangle \ge \langle 0_{\Lambda_i}|H_{\Lambda_i}|0_{\Lambda_i}\rangle - \|V_i\| \ge \langle 0_{\Lambda_i}|H_{\Lambda_i}|0_{\Lambda_i}\rangle - g, \tag{11}$$

which yields

$$\langle\phi|H|\phi\rangle - \langle 0|H|0\rangle \le 2g. \tag{12}$$

By combining the inequalities (9) and (12), we obtain the inequality (7). □

2. *Main assumption*

In order to give the condition under which the area law is obtained, we define the interaction operator $V_{X,Y}(\Lambda_0)$ between two subsystems $X \subset \Lambda$ and $Y \subset \Lambda$ as follows (see Supplementary Figure 1):

$$V_{X,Y}(\Lambda_0) := \sum_{\substack{Z:Z\subset\Lambda_0 \\ Z\cap X\neq\emptyset, Z\cap Y\neq\emptyset}} h_Z. \tag{13}$$

Here, $V_{X,Y}(\Lambda_0)$ is defined for an arbitrary subset $\Lambda_0 \subset \Lambda$ such that $X \sqcup Y \subset \Lambda_0$. Note that the operator $V_{X,Y}(\Lambda_0)$ composed of the interaction terms h_Z between X and Y which are supported on $\Lambda_0 \subset \Lambda$. In the case of $k = 2$ as in Eq. (2), $V_{X,Y}(\Lambda_0)$ does not depend on the choice of Λ_0 and is simply given by

$$V_{X,Y}(\Lambda_0) = V_{X,Y}(X \sqcup Y) = \sum_{i\in X} \sum_{j\in Y} h_{i,j}, \tag{14}$$

where each of the terms h_Z is given by $h_{i,j}$. We utilized the form (14) in Eq. (4) of the main paper. On the other hand, in the case of $k \ge 3$, $V_{X,Y}(\Lambda_0)$ usually depends on choice of the subsystem Λ_0.

Throughout the paper, we assume the following algebraic decay of $\|V_{X,Y}(\Lambda_0)\|$.

Assumption 1. *Let X and Y be arbitrary concatenated subsystems with* $\mathrm{dist}(X,Y) = r$*. Then, for arbitrary choice of $\Lambda_0 \subset \Lambda$, there exists a constant g_0 (≥ 1) such that*

$$\|V_{X,Y}(\Lambda_0)\| \le g_0 r^{-\bar{\alpha}} \tag{15}$$

with $\bar{\alpha} > 0$. In Supplementary Note 1 C, we will discuss how the parameters $\{g_0, \bar{\alpha}\}$ are given in terms of $\{J, \alpha\}$ in Eq. (3).

The assumption is utilized in deriving the inequalities (46) and (55). The former inequality (46) implies a finite upper bound of the boundary interaction along a cut (see Supplementary Figure 2 in Supplementary Note 1 D 1). The latter inequality (55) is an essential tool to upper-bound the error in truncating the long-range interaction (see also Supplementary Figure 2).

In order to discuss the entanglement entropy, we spatially decompose the total space into two subsystems L and R (see Supplementary Figure 2 for example), respectively. We denote the reduced density matrix of the ground state in L by ρ_L:

$$\rho_L = \mathrm{tr}_R(|0\rangle\langle 0|), \tag{16}$$

where $\mathrm{tr}_R(\cdots)$ denotes the partial trace operation with respect to the subsystem R. We define the entanglement entropy of this decomposition $\Lambda = L \sqcup R$ as

$$S(L) := -\mathrm{tr}(\rho_L \log \rho_L) = -\mathrm{tr}(\rho_R \log \rho_R). \tag{17}$$

Our purpose is to bound the entropy $S(L)$ from above by a function of d, Δ and $\{k, g_0, \bar{\alpha}\}$ (see also Table I).

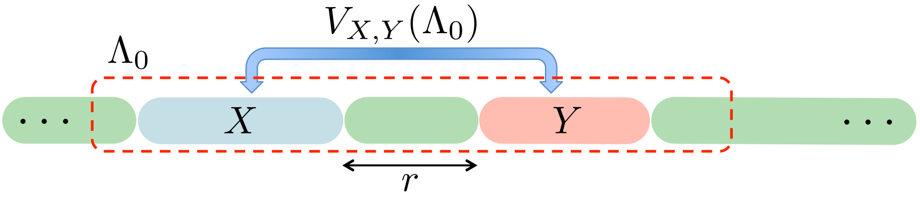

Supplementary Figure 1. Subsystem-subsystem interaction. In order to define $V_{X,Y}(\Lambda_0)$, we pick up all the terms h_Z in $\Lambda_0 \subset \Lambda$ which connects X and Y. Our condition for the area law is that the norm $\|V_{X,Y}(\Lambda_0)\|$ decays algebraically with respect to the distance between X and Y.

3. Schmidt rank

We consider an operator O and define the Schmidt rank $\mathrm{SR}(O,X)$ for $X \subseteq \Lambda$ as the minimum integer such that

$$O = \sum_{m=1}^{\mathrm{SR}(O,X)} O_{X,m} \otimes O_{X^{\mathrm{c}},m}, \tag{18}$$

where $O_{X,m}$ and $O_{X^{\mathrm{c}},m}$ are supported on the subsystems X and X^{c} (the complementary set of X), respectively. We also define the Schmidt rank $\mathrm{SR}(|\psi\rangle, X)$ of a state $|\psi\rangle$ as follows:

$$|\psi\rangle = \sum_{m=1}^{\mathrm{SR}(|\psi\rangle,X)} \mu_m |\psi_{X,m}\rangle \otimes |\psi_{X^{\mathrm{c}},m}\rangle, \tag{19}$$

which is the Schmidt decomposition. Especially in considering $\mathrm{SR}(O,L)$ (or $\mathrm{SR}(|\psi\rangle,L)$) for the target decomposition $\Lambda = L \sqcup R$, we simply denote $\mathrm{SR}(O)$ (or $\mathrm{SR}(|\psi\rangle)$) omitting the subsystem dependence.

B. Main results

Theorem 1 (Area law for 1D long-range interacting systems)**.** *For an arbitrary bipartition of the system $\Lambda = L \sqcup R$. The entanglement entropy $S(|0\rangle)$ is bounded from above by*

$$S(|0\rangle) \le c_0 \log^2(d) \left(\frac{\log(d)}{\Delta}\right)^{1+2/\bar{\alpha}} \log^{3+3/\bar{\alpha}}\left(\frac{\log(d)}{\Delta}\right), \tag{20}$$

where c_0 is a constant which depends only on k, g_0, $\bar{\alpha}$, which has a finite value in the limit of $\bar{\alpha} \to \infty$[*1]*. Also, there exists a quantum state $|\psi\rangle$ such that*

$$\||0\rangle - |\psi\rangle\| \le \delta \tag{21}$$

with the Schmidt rank of

$$\log\left[\mathrm{SR}(|\psi\rangle)\right] = \mathcal{O}(\bar{\alpha}^{-1}\log^{5/2}(1/\delta)) + \mathcal{O}(\log^{3/2}(1/\delta)) \tag{22}$$

for sufficiently small δ, where $\mathrm{SR}(|\psi\rangle)$ was defined in (19).

Supplementary Table I. Fundamental parameters in our statement

**Parameters**	**Definition**
d	Dimension of the Hilbert space of one site
Δ	Spectral gap between the ground state and the first excited state
k	Maximum number of sites involved in interactions (see Eq. (1))
g_0	Defined in Assumption 1 (see Ineq. (15))
$\bar{\alpha}$	Defined in Assumption 1 (see Ineq. (15))

[*1] In the main text, for the sake of readability, we do not introduce the quantity (5), and hence we explain that the coefficient c in Ineq. (6) depends on $\{\alpha, J, B, k, g_0, \bar{\alpha}\}$. However, we here set $g = 1$ without loss of generality taking an appropriate unit, and hence the coefficient c_0 depends only on $\{k, g_0, \bar{\alpha}\}$. We emphasize that there are no inconsistencies between these.

This theorem implies that in the limit of $\bar{\alpha} \to \infty$, the entanglement entropy is given by

$$S(|0\rangle) \lesssim c_0 \frac{\log^3(d)}{\Delta} \quad \text{for} \quad \bar{\alpha} \to \infty \tag{23}$$

up to a logarithmic correction. This reproduces the results by Arad-Kitaev-Landau-Vazirani for short-range interacting systems [2].

By applying Lemma 1 in Ref. [3] to the above theorem, we immediately obtain the efficiency of the MPS representation of the ground state $|0\rangle$ (see Method section in the main text for the proof).

Corollary 1. *Let us assume $\bar{\alpha} = \mathcal{O}(1)$. Then, under the same set up of Theorem 1, there exists a matrix product state $|\psi_D\rangle$ with its bond dimension $D = \exp[c'\bar{\alpha}^{-1} \log^{5/2}(1/\delta)]$ (c': constant) such that*

$$\|\mathrm{tr}_{X^{\mathrm{c}}}(|\psi_D\rangle\langle\psi_D|) - \mathrm{tr}_{X^{\mathrm{c}}}(|0\rangle\langle 0|)\|_1 \le \delta |X| \tag{24}$$

for an arbitrary concatenated subregion X, where $\|\cdot\|_1$ is the trace norm and $|X|$ denotes the cardinality of X. We here denote the complementary subset of X by $X^{\mathrm{c}} := \Lambda \setminus X$.

From the corollary, in order to approximate the ground state $|0\rangle$ by using the matrix product states with $\delta = 1/\mathrm{poly}(n)$, we need the bond dimension D_{MPS} of order

$$D_{\mathrm{MPS}} = \exp[c\bar{\alpha}^{-1} \log^{5/2}(n)] = n^{c\bar{\alpha}^{-1} \log^{3/2}(n)}. \tag{25}$$

Hence, the simulation of the gapped ground states requires quasi-polynomial computational time. This contrasts to the short-range interacting cases, where the sufficient bond-dimension for $\delta = 1/\mathrm{poly}(n)$ is sub-linear [2] as

$$D'_{\mathrm{MPS}} = \exp[c' \log^{3/4}(n)]. \tag{26}$$

C. Specific values of $\bar{\alpha}$ and g_0

We first derive the upper bound for $\|V_{X,Y}(\Lambda_0)\|$ only from the inequality (3). For this purpose, we prove the following lemma (see Supplementary Note 1 C 1):

Lemma 1. *Let $X \subset \Lambda$ be an arbitrary concatenated subsystem and $Y \subset \Lambda$ be the subsystem such that* $\mathrm{dist}(X, Y) = r$ *(see Supplementary Figure 1). Then, the norm of $V_{X,Y}(\Lambda_0)$ is bounded from above by*

$$\|V_{X,Y}(\Lambda_0)\| \le \frac{J\alpha}{\alpha - 2} r^{-\alpha+2}. \tag{27}$$

Hence, we have

$$g_0 = \frac{\alpha J}{\alpha - 2}, \quad \bar{\alpha} = \alpha - 2, \tag{28}$$

in the inequality (15).

From the lemma, the assumption 1 is always satisfied for $\alpha > 2$. This lower bound of α is the most general one and applied to arbitrary quantum many-body systems. On the other hand, the condition $\alpha > 2$ can be relaxed if we consider a specific class of Hamiltonians. For example, we here consider a fermion system with long-range hopping as follows:

$$H = \sum_{i<j} \frac{1}{r_{i,j}^{\alpha}} (A_{i,j} a_i a_j^\dagger + B_{i,j} a_i a_j + \mathrm{h.c.}) + V \quad \text{with} \quad |A_{i,j}|,\ |B_{i,j}| \le \tilde{J}, \tag{29}$$

where $r_{i,j} := \mathrm{dist}(i, j)$ and $\{a_i^\dagger, a_i\}_{i=1}^n$ are the creation and the annihilation operators for fermion, and V is arbitrary short-range interacting terms such as $a_i a_i^\dagger a_j a_j^\dagger$ with $r_{i,j} \le \mathcal{O}(1)$. In this case, we can prove the following lemma:

Lemma 2. *Let $X \subseteq \Lambda$ be an arbitrary concatenated subsystem and $Y \subseteq \Lambda$ be the subsystem such that* $\mathrm{dist}(X, Y) =$ *r. We assume that the distance r is larger than the short-range interaction length which is given by V. Then, the norm of $V_{X,Y}(\Lambda_0)$ is bounded from above by*

$$\|V_{X,Y}(\Lambda_0)\| \le 4\tilde{J} \sqrt{\frac{2\alpha}{2\alpha - 1} \frac{2\alpha - 1}{2\alpha - 3}} r^{-\alpha+3/2}, \tag{30}$$

Hence, we have

$$g_0 = 4\tilde{J} \sqrt{\frac{2\alpha}{2\alpha - 1} \frac{2\alpha - 1}{2\alpha - 3}}, \quad \bar{\alpha} = \alpha - 3/2, \tag{31}$$

in the inequality (15).

From the lemma, the assumption 1 is satisfied for $\alpha > 3/2$ (instead of $\alpha > 2$). In this way, depending on the situation, the condition for the power exponent α can be loosen. Other cases which gives a better condition than $\alpha > 2$ include quantum many-body systems with random long-range interactions [4].

1. *Proof of Lemma 1*

For the proof, we estimate the upper bound of

$$\overline{V}_{X,Y} := \sum_{Z:Z\cap X\neq\emptyset, Z\cap Y\neq\emptyset} \|h_Z\|, \tag{32}$$

which clearly gives an upper bound of $\|V_{X,Y}(\Lambda_0)\|$ for arbitrary choices of $\Lambda_0 \subset \Lambda$. From the inequality (3), we first obtain for an arbitrary integer $\tilde{r}$

$$\sum_{Z:Z\ni i,\mathrm{diam}(Z)\geq \tilde{r}} \|h_Z\| \leq \sum_{x=\tilde{r}}^{\infty} Jx^{-\alpha} \leq J\tilde{r}^{-\alpha} + J\int_{\tilde{r}}^{\infty} x^{-\alpha}dx \leq \frac{\alpha J}{\alpha-1}\tilde{r}^{-\alpha+1}, \tag{33}$$

where we use $\tilde{r}^{-\alpha} \leq \tilde{r}^{-\alpha+1}$ in the last inequality.

Second, in order to estimate the upper bound of $\overline{V}_{X,Y}$, we define $Y = \{i_0+1, i_0+2, \ldots, i_0+n_Y\}$ with $n_Y = |Y|$. Without loss of generality, we assume that the subsystem Y locates on the right side of X. Then, because of $\mathrm{dist}(i_0+j, X) = r-1+j$ we have

$$\begin{aligned}\sum_{Z:Z\cap X\neq\emptyset, Z\cap Y\neq\emptyset} \|h_Z\| &\leq \sum_{j=1}^{n_Y} \sum_{\substack{Z:Z\ni i_0+j \\ \mathrm{diam}(Z)\geq r+j-1}} \|h_Z\| \leq \sum_{j=1}^{n_Y} \frac{\alpha J}{\alpha-1}\frac{1}{(r+j-1)^{\alpha-1}} \\ &\leq \frac{\alpha J}{\alpha-1}\left(r^{-\alpha+1} + \int_r^{\infty} x^{-\alpha+1}dx\right) \leq \frac{\alpha J}{\alpha-2} r^{-\alpha+2},\end{aligned} \tag{34}$$

where we use the inequality (33) in the second inequality. This completes the proof. □

2. *Proof of Lemma 2*

Without loss of generality, we assume that the subsystem Y locates on the right side of X. Then, we notice that $V_{X,Y}(\Lambda_0)$ is given by

$$\begin{aligned}V_{X,Y}(\Lambda_0) = V_{X,Y}(X\sqcup Y) &= \sum_{i\in X}\sum_{j\in Y} \frac{1}{r_{i,j}^{\alpha}}(A_{i,j}a_i a_j^{\dagger} + B_{i,j}a_i a_j + \mathrm{h.c.}) \\ &= \sum_{i\in X} a_i \sum_{j\in Y} \frac{A_{i,j}a_j^{\dagger} + B_{i,j}a_j}{r_{i,j}^{\alpha}} + \mathrm{h.c.},\end{aligned} \tag{35}$$

which gives the upper bound of $\|V_{X,Y}(\Lambda_0)\|$ as

$$\|V_{X,Y}(\Lambda_0)\| \leq 2\sum_{i\in X}\left\|\sum_{j\in Y}\frac{A_{i,j}a_j^{\dagger}}{r_{i,j}^{\alpha}}\right\| + 2\sum_{i\in X}\left\|\sum_{j\in Y}\frac{B_{i,j}a_j}{r_{i,j}^{\alpha}}\right\|. \tag{36}$$

By using the condition $\|A_{i,j}\| \leq \tilde{J}$ in (29), the first term is bounded from above as

$$\left\|\sum_{j\in Y}\frac{A_{i,j}a_j^{\dagger}}{r_{i,j}^{\alpha}}\right\| \leq \left(\sum_{j\in Y}\frac{|A_{i,j}|^2}{r_{i,j}^{2\alpha}}\right)^{1/2} \leq \tilde{J}\left(\sum_{x=1}^{\infty}(r_i+x-1)^{-2\alpha}\right)^{1/2} \leq \tilde{J}\sqrt{\frac{2\alpha}{2\alpha-1}}r_i^{-\alpha+1/2}, \tag{37}$$

where we define $r_i = \mathrm{dist}(i, Y)$ and utilize the inequality

$$\sum_{x=1}^{\infty}(r_i+x-1)^{-2\alpha} \leq r_i^{-2\alpha} + \int_{r_i}^{\infty} x^{-2\alpha}dx = r_i^{-2\alpha} + \frac{r_i^{-2\alpha+1}}{2\alpha-1} \leq \frac{2\alpha}{2\alpha-1}r_i^{-2\alpha+1}. \tag{38}$$

The summation with respect to $i \in X$ reduces to the summation from $r_i = r$ to $r_i = r + |X| - 1$. Hence, we obtain

$$\sum_{i\in X}\left\|\sum_{j\in Y}\frac{A_{i,j}a_j^{\dagger}}{r_{i,j}^{\alpha}}\right\| \leq \sum_{r_i=r}^{\infty}\tilde{J}\sqrt{\frac{2\alpha}{2\alpha-1}}r_i^{-\alpha+1/2} \leq \tilde{J}\sqrt{\frac{2\alpha}{2\alpha-1}}\frac{2\alpha-1}{2\alpha-3}r^{-\alpha+3/2}. \tag{39}$$

We can derive the same inequality for the summation of $B_{i,j}a_j/r_{i,j}^{\alpha}$ with respect to $i \in X$ and $j \in Y$. By applying the above inequality to (36), we prove the inequality (30). □

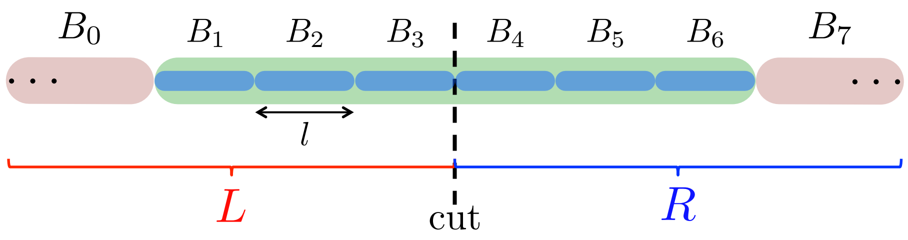

Supplementary Figure 2. Interaction truncation in the Hamiltonian. Across the dashed line indicated at the center of the system, we decompose the system into $(q+2)$-blocks ($q = 6$ in the above picture). Each of the blocks $\{B_s\}_{s=1}^q$ has a length l, and the edge blocks B_0 and B_{q+1} extend to the left and right ends of the system, respectively. Then, we truncate all the interactions between separated blocks. Because we only truncate the long-range interactions around the cut, the truncated Hamiltonian $H_{\rm t}$ in Eq. (45) is still close to the original Hamiltonian H as shown in Lemma 3.

D. Outline of the proof

1. *Preliminaries*

[Approximate ground state projection (AGSP)]

We here introduce the projection operator onto the ground state. It is usually difficult to construct the exact ground-state projection operator, and hence we consider an approximate one as

$$K|0\rangle \simeq |0\rangle \quad \text{and} \quad \|K(1-|0\rangle\langle 0|)\| \simeq 0, \tag{40}$$

where $(1-|0\rangle\langle 0|)$ is equivalent to the projection operator onto the space of the excited eigenstates. We assume that K is a Hermitian operator (i.e., $K = K^\dagger$). In the following, we characterize the approximate ground state projection (AGSP) operators by three parameters $\{\delta_K, \epsilon_K, D_K\}$. Let $|0_K\rangle$ be a quantum state that is invariant by K such that

$$K|0_K\rangle = |0_K\rangle. \tag{41}$$

Then, the parameters are defined by the following inequalities:

$$\||0\rangle - |0_K\rangle\| \le \delta_K, \quad \|K(1-|0_K\rangle\langle 0_K|)\| \le \epsilon_K, \quad \text{and} \quad \mathrm{SR}(K) \le D_K. \tag{42}$$

The second inequality implies for arbitrary $|\psi_\perp\rangle$ which is orthogonal to $|0_K\rangle$ (i.e., $\langle\psi_\perp|0_K\rangle = 0$)

$$\|K|\psi_\perp\rangle\| = \|K(1-|0_K\rangle\langle 0_K|)|\psi_\perp\rangle\| \le \epsilon_K. \tag{43}$$

Recall that in Supplementary Note 1 A 3 we denote $\mathrm{SR}(O,L)$ by $\mathrm{SR}(O)$ for the simplicity.

Note that the state $|0_K\rangle$ is an approximate ground state if $\delta_K \simeq 0$. When $\delta_K = \epsilon_K = 0$, the operator K is the exact ground state projection, namely $K = |0\rangle\langle 0|$. In the standard definition of the AGSP [2, 5, 6], we do not need to consider the parameter δ_K explicitly. However, in the present case of the long-range interacting systems, the error of $\||0\rangle - |0_K\rangle\|$ is too large to ignore and we have to correctly take the effect of δ_K into account.

[Interaction-truncated Hamiltonian]

We first decompose the total system into B_0, $\{B_s\}_{s=1}^q$ and B_{q+1} with $\bigcup_{s=0}^{q+1} B_s = \Lambda$, where q is an even integer ($q \ge 2$) and we choose B_s ($1 \le s \le q$) such that $|B_s| = l$. Note that we express the subsets L and R in terms of these blocks:

$$L = \bigcup_{s=0}^{q/2} B_s, \quad R = \bigcup_{s=q/2+1}^{q+1} B_s. \tag{44}$$

We now truncate all the interactions between the non-adjacent blocks. After the truncation, only the interactions between the adjacent blocks exist, namely

$$H_{\rm t} = \sum_{s=0}^{q+1} h_s + \sum_{s=0}^{q} h_{s,s+1}, \tag{45}$$

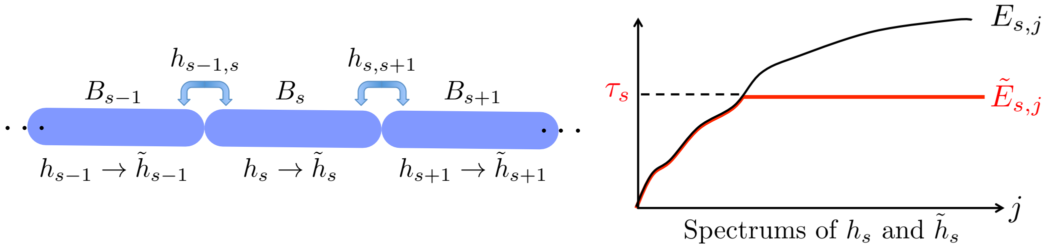

Supplementary Figure 3. Schematic picture of our effective Hamiltonian. In the effective Hamiltonian $\tilde{H}_{\rm t}$, we modify the energy spectrum so that the energy higher than τ_s is constant in each of the Hamiltonians $\{h_s\}_{s=0}^{q+1}$, whereas the other part of the Hamiltonian (i.e., $\{h_{s,s+1}\}_{s=0}^{q}$) is the same as the original Hamiltonian. As long as we focus on the low-energy spectrum, the effective Hamiltonian looks almost the same as the original Hamiltonian. It will be shown in Theorem 5 that the accuracy exponentially approaches with the cut-off energy τ.

where $h_{s,s+1} := V_{B_s,B_{s+1}}(B_s \sqcup B_{s+1})$ by choosing $X = B_s$, $Y = B_{s+1}$ and $\Lambda_0 = B_s \sqcup B_{s+1}$ in the definition of $V_{X,Y}(\Lambda_0)$ in Eq. (13), and h_s collects all the terms supported only on B_s. We notice that the assumption 1 with $r = 1$ gives

$$\|h_{s,s+1}\| \le g_0. \tag{46}$$

In the following, we describe $H_{\rm t}$ as

$$H_{\rm t} = \sum_{s=0}^{q} H_s, \quad \text{with} \quad H_0 = h_{s=0} + h_{s=1} + h_{s=0,s=1}, \quad H_s = h_{s+1} + h_{s,s+1} \ (s \ge 1). \tag{47}$$

We denote by $|0_{\rm t}\rangle$ the ground state of the truncated Hamiltonian $H_{\rm t}$. Throughout the paper, we take the origin of the energy so that $E_{{\rm t},0} = 0$, where $E_{{\rm t},0}$ is the ground-state energy of $H_{\rm t}$. Note that we set the origin of the energy through not the original Hamiltonian H, but the truncated Hamiltonian $H_{\rm t}$.

[Effective Hamiltonian by multi-energy cut-off]

In the construction of the AGSP operator (40), we need an effective Hamiltonian $\tilde{H}_{\rm t}$ which has a small norm but possesses almost the same low-energy properties as the original Hamiltonian $H_{\rm t}$. For the construction of such an effective Hamiltonian, we apply the energy cut-off in Ref. [7] to the Hamiltonian $H_{\rm t}$ in Eq. (45). For each of the block Hamiltonian $\{h_s\}_{s=0}^{q+1}$, we apply the following energy cut-off (Supplementary Figure 3):

$$\tilde{h}_s = \sum_{E_{s,j} < \tau_s} E_{s,j} |E_{s,j}\rangle\langle E_{s,j}| + \sum_{E_{s,j} \ge \tau_s} \tau_s |E_{s,j}\rangle\langle E_{s,j}| \tag{48}$$

with

$$\tau_s = E_{s,0} + \tau, \tag{49}$$

where $\{E_{s,j}, |E_{s,j}\rangle\}_j$ are the eigenvalues and the eigenstates of h_s, respectively. Then, the effective Hamiltonian $\tilde{H}_{\rm t}$ is given by

$$\tilde{H}_{\rm t} = \sum_{s=0}^{q+1} \tilde{h}_s + \sum_{s=0}^{q} h_{s,s+1}. \tag{50}$$

Note that we do not make any changes for the interaction terms $\{h_{s,s+1}\}_{s=0}^{q}$. We notice that Propositions 4 and 8 on the Schmidt rank are applicable to the effective Hamiltonian $\tilde{H}_{\rm t}$. We denote by $|\tilde{0}_{\rm t}\rangle$ the ground state of the effective Hamiltonian $\tilde{H}_{\rm t}$.

2. Brief outline

We here show the high-level overview of the area-law proof in one-dimensional long-range interacting systems (see Supplementary Figure 4). We have also shown it in Method section in the main text.

We first completely break the entanglement entropy of the ground state by performing a projection operator onto a product state with respect to the partition $L \sqcup R$. Note that the entanglement entropy is equal to zero

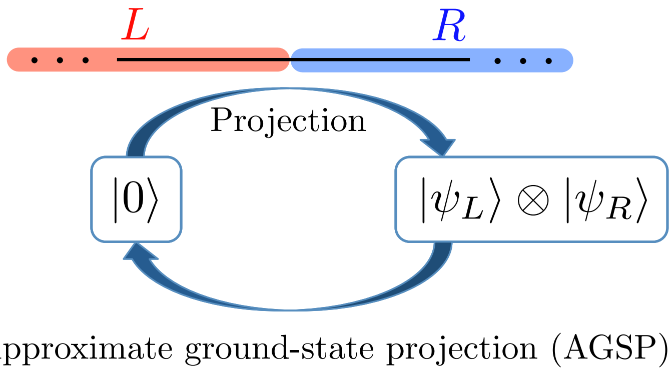

Supplementary Figure 4. Schematic picture of the proof of the one-dimensional area law. We decompose the total system Λ into the two subsystems L and R. In the proof of the area law, we first perform a projection onto a product state which has the maximum overlap with the ground state $|0\rangle$. We then recover the original state from this product state by the use of the approximate ground state projector K (AGSP) as in Eq. (40). The entanglement entropy can be bounded from above by the entanglement generation of AGSP (the Schmidt rank of K) because the product state contains no entanglement.

for product states. We second consider a reverse operator K from the product state to the ground state $|0\rangle$. The operator K is now taken as an approximate ground state projector (AGSP) as in Eq. (40).

We need to consider the following problems: how small is the overlap between the ground state and the product state? On this problem, we can utilize the bootstrapping lemma [2, 8] (i.e., Lemma 7 in our manuscript); that is, under a good choice of the AGSP operator, an overlap between the ground state and a product state is lower-bounded by using the AGSP parameters ϵ_K and D_K. Roughly speaking, we need to find an AGSP operator which satisfies $\epsilon_K^2 D_K \le 1/2$.

The primary problem is how to construct the AGSP operators with appropriate properties to apply the basic strategy. For the purpose, we first perform the truncation of long-range interactions in order to suppress the Schmidt rank D_K. If we simply truncate all the long-range interactions in the entire region, the truncated Hamiltonian $H_{\rm t}$ and the original Hamiltonian H is extensively different, namely $\|H - H_{\rm t}\| \approx \mathcal{O}(n)$. This may completely change the ground state's property. To avoid it, we truncate the long-range interaction only around the cut between L and R (see Supplementary Figure 2). This truncation ensures the small norm distance between the original Hamiltonian and the truncated Hamiltonian, which preserves the gap condition of $H_{\rm t}$ (Lemma 3) and ensures the closeness between both of the ground states (Lemma 4). This imposes the following condition for the block size l and the block number q:

$$ql^{-\bar{\alpha}} \lesssim 1. \tag{51}$$

In the construction of the AGSP operator from the Chebyshev polynomial, the projection error ϵ_K strongly depends on the norm of the Hamiltonian (see the inequality (164)). Hence, in the second step, we perform the multi-energy cut-off in each of the blocks as in Supplementary Figure 3 to define an effective Hamiltonian $\tilde{H}_{\rm t}$. Roughly speaking, the norm of the effective Hamiltonian in Eq. (50) is given by $\mathcal{O}(q\tau)$. The gap preservation and the closeness of the ground state is still ensured as long as the cut-off energy satisfies $\tau \gg \log(q)$ (Theorem 5). In the standard construction of the effective Hamiltonian [2, 7], we suppose to perform the energy cut-off only in the edge blocks (i.e., B_0 and B_{q+1}). However, this simple procedure allows us to prove the long-range area law only in the short-range power-exponent regimes (i.e., $\alpha > 3$). The multi-energy cut-off is crucial to prove the area law even in the long-range power-exponent regimes (i.e., $\alpha \le 3$).

We then need to derive basic properties of the AGSP so that they meet our present setup and purposes. In Proposition 2, we lower-bound the overlap between the ground state and low-entangled state. We then derive the upper bound of the entanglement entropy by using a sequence of the AGSP operators (Proposition 3). As for the connection between the AGSP parameters $\{D_K, \epsilon_K, \delta_K\}$ and polynomials of the effective Hamiltonian $\tilde{H}_{\rm t}$, we derive Lemma 8 and Proposition 4 for the Schmidt rank, and give Lemma 11 to upper-bound the projection error ϵ_K in terms of the norm of the effective Hamiltonian $\|\tilde{H}_{\rm t}\|$. Then, by using the mth order Chebyshev polynomial, the Schmidt rank D_K and the error of the AGSP ϵ_K is roughly given by

$$D_K \sim e^{m \log(q)/q + q^{1+1/\bar{\alpha}}}, \quad \epsilon_K \sim e^{-m/\sqrt{q \log q}} \tag{52}$$

under the condition (51) (see the inequality (199)), where we use Proposition 4 in estimating the Schmidt rank. These estimations for D_K and ϵ_K ensure the existence of $\{q, m\}$ which satisfies $\epsilon_K^2 D_K \le 1/2$ for the condition of the bootstrapping lemma (Proposition 2). We then prove the existence of a quantum state which is close to the original ground state with an error smaller than 1/2 and has a small Schmidt rank (Proposition 6).

Finally, based on Proposition 3 and Lemma 8, we construct the complete ground state $|0\rangle$ to upper-bound the entanglement entropy (Proposition 7). This yields our main Theorem 1. In Supplementary Figure 5, we give the flow chart of the whole proof.

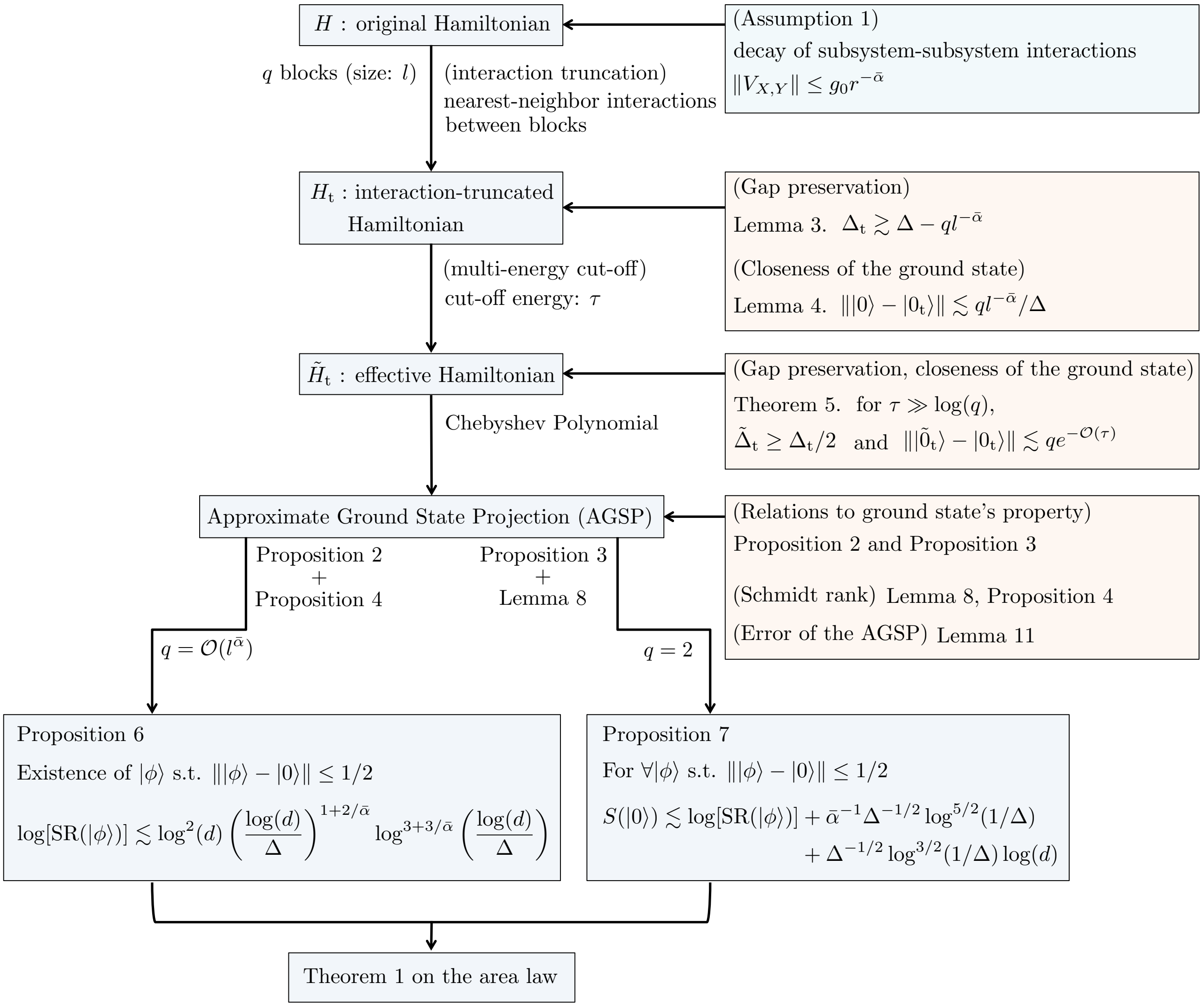

Supplementary Figure 5. Flow chart of the proof.

Supplementary Note 2. DETAILS OF TECHNICAL LEMMAS, PROPOSITIONS AND SUB-THEOREMS

Before giving the proof of Theorem 1, we show technical lemmas, propositions and sub-theorems, which are the key ingredients to prove the theorem. Several lemmas can be trivially derived from the previous analyses in Refs [2, 5–8] by extending their setups to the present setup. We show the details of almost all the lemmas, propositions, and sub-theorems so that all the readers can follow the proofs.

In the following analyses, we assume the open-boundary condition. In the periodic-boundary condition, we can also define the truncated Hamiltonian in the same way (see Supplementary Figure 6) by regarding the system as a one-dimensional ladder.

A. Gap condition for the truncated Hamiltonian $H_{\rm t}$

First of all, we analyze the ground state of the truncated Hamiltonian $H_{\rm t}$. For the purpose, we need to clarify the gap condition for $H_{\rm t}$. It is ensured by the following lemma which gives the norm difference between H and $H_{\rm t}$:

Lemma 3. *The norm distance between H and $H_{\rm t}$ is bounded from above by*

$$\|\delta H_{\rm t}\| \le g_0 q l^{-\bar{\alpha}}, \tag{53}$$

where we define $\delta H_{\rm t} := H - H_{\rm t}$. Also, the spectral gap $\Delta_{\rm t}$ of $H_{\rm t}$ is bounded from below by

$$\Delta_{\rm t} \ge \Delta - 2g_0 q l^{-\bar{\alpha}}. \tag{54}$$

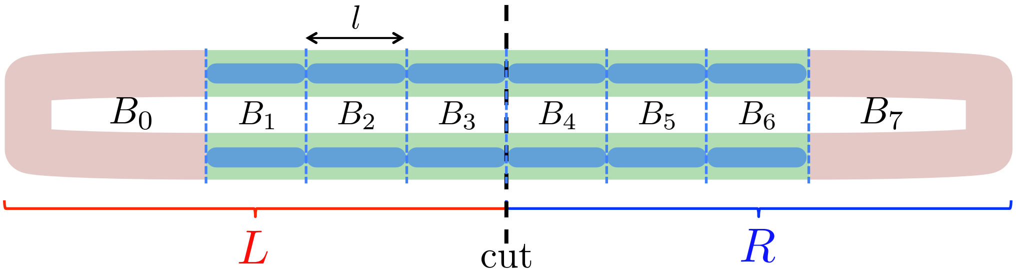

Supplementary Figure 6. Interaction truncation in the Hamiltonian in the periodic-boundary condition. In this case, we can apply the same discussion as in the case of the open-boundary condition by regarding the system as a one-dimensional ladder.

Proof of Lemma 3. We define $X_s := \bigcup_{j\geq s+2} B_j$ and $\Lambda_s = \bigcup_{j\geq s} B_j$ for $s = 0, 1, 2, \ldots, q-1$. Then, from the definition of the truncated Hamiltonian (45), we obtain

$$\|H - H_{\rm t}\| \leq \sum_{s=0}^{q-1} \|V_{B_s,X_s}(\Lambda_s)\|. \tag{55}$$

By using the assumption 1, the inequality (15) gives

$$\|V_{B_s,X_s}(\Lambda_s)\| \leq g_0 l^{-\bar{\alpha}}. \tag{56}$$

From the inequalities (55) and (56), we obtain the inequality (53).

In order to derive the inequality (54), we utilize the Weyl's inequality:

$$|E_j - E_{{\rm t},j}| \leq \|\delta H_{\rm t}\| \leq g_0 q l^{-\bar{\alpha}}, \tag{57}$$

where $\{E_j\}_{j\geq 0}$ and $\{E_{{\rm t},j}\}_{j\geq 0}$ are eigenvalues of H and $H_{\rm t}$ in ascending order (i.e., $E_j \leq E_{j'}$ for $j \leq j'$), respectively. We thus obtain the inequality (54) as follows:

$$\Delta_{\rm t} = E_{{\rm t},1} - E_{{\rm t},0} \geq E_1 - E_0 - |E_1 - E_{{\rm t},1}| - |E_0 - E_{{\rm t},0}| \geq \Delta - 2\|\delta H_{\rm t}\|. \tag{58}$$

This completes the proof. □

B. Perturbation of the ground state

Lemma 4. *Under the assumption of* $4\|\delta H_{\rm t}\| < \Delta$*, the original ground state* $|0\rangle$ *have an overlap with that of the truncated Hamiltonian* $|0_{\rm t}\rangle$ *as follows:*

$$\||0\rangle - |0_{\rm t}\rangle\| \leq \frac{\|\delta H_{\rm t}\|}{\Delta - 4\|\delta H_{\rm t}\|}. \tag{59}$$

Also, for an arbitrary quantum state $|\phi\rangle$*, the norm distance between* $|0\rangle$ *and* $|\phi\rangle$ *is bounded from above by*

$$\||0\rangle - |\phi\rangle\| \leq \||0_{\rm t}\rangle - |\phi\rangle\| + \frac{\|\delta H_{\rm t}\|}{\Delta - 4\|\delta H_{\rm t}\|}. \tag{60}$$

Proof of Lemma 4. The inequality (60) is simply derived from the triangle inequality, and hence we need to prove the inequality (59). We first expand $|0\rangle$ as follows:

$$|0\rangle = \zeta_1|0_{\rm t}\rangle + \zeta_2|\psi_{{\rm t},\perp}\rangle, \tag{61}$$

where $\langle 0_{\rm t}|\psi_{{\rm t},\perp}\rangle = 0$ and we choose the phase term of $|0_{\rm t}\rangle$ so that $\langle 0|0_{\rm t}\rangle$ has a positive real value, namely $|\zeta_1| = |\langle 0|0_{\rm t}\rangle| = \langle 0|0_{\rm t}\rangle = \zeta_1$. Then, the coefficients $\{\zeta_1, \zeta_2\}$ is determined by the eigen-problem of the following matrix:

$$\begin{pmatrix} \langle 0_{\rm t}|H|0_{\rm t}\rangle & \langle 0_{\rm t}|H|\psi_{{\rm t},\perp}\rangle \\ \langle \psi_{{\rm t},\perp}|H|0_{\rm t}\rangle & \langle \psi_{{\rm t},\perp}|H|\psi_{{\rm t},\perp}\rangle \end{pmatrix} =: \begin{pmatrix} f_0 & f \\ f^* & f_\perp \end{pmatrix}. \tag{62}$$

Then, the ground-state energy of H is formally given by

$$E_0 = \frac{f_0 + f_\perp - \sqrt{(f_0 - f_\perp)^2 + 4|f|^2}}{2}, \tag{63}$$

and the corresponding coefficients $\{\zeta_1, \zeta_2\}$ are

$$\{\zeta_1, \zeta_2\} \propto \left\{ f_\perp - f_0 + \sqrt{(f_\perp - f_0)^2 + 4|f|^2}, -2f^* \right\}. \tag{64}$$

Then, if $f_\perp > f_0$, we have

$$\frac{|\zeta_2|}{\zeta_1} = \frac{2|f|/(f_\perp - f_0)}{1 + \sqrt{1 + 4|f|^2/(f_\perp - f_0)^2}} \le \frac{|f|}{f_\perp - f_0}, \tag{65}$$

where we will prove the assumption $f_\perp > f_0$ afterward.

From the equation $\zeta_1^2 + |\zeta_2|^2 = 1$, we obtain

$$\zeta_1 = \frac{1}{\sqrt{1 + |\zeta_2/\zeta_1|^2}} \ge 1 - \frac{1}{2}\left|\frac{\zeta_2}{\zeta_1}\right|^2. \tag{66}$$

On the other hand, we have

$$\| |0\rangle - |0_{\rm t}\rangle \|^2 = (\zeta_1 - 1)^2 + |\zeta_2|^2 = 2 - 2\zeta_1, \tag{67}$$

where we use the fact that $\zeta_1 \in \mathbb{R}^+$. By combining the inequalities (65), (66) and (67), we obtain

$$\| |0\rangle - |0_{\rm t}\rangle \|^2 \le \left|\frac{\zeta_2}{\zeta_1}\right|^2 \le \left(\frac{|f|}{f_\perp - f_0}\right)^2, \tag{68}$$

which reduces to

$$\| |0\rangle - |0_{\rm t}\rangle \| \le \frac{|f|}{f_\perp - f_0}. \tag{69}$$

The remaining task is to obtain the upper bound or the lower bound of $|f|$, $f_\perp$ and f_0. First, we have

$$|f| = |\langle 0_{\rm t}|H|\psi_{{\rm t},\perp}\rangle| \le \|H|0_{\rm t}\rangle\| = \|\delta H_{\rm t}|0_{\rm t}\rangle\| \le \|\delta H_{\rm t}\|, \tag{70}$$

where we use $H_{\rm t}|0_{\rm t}\rangle = 0$. Also, we have

$$f_\perp = \langle \psi_{{\rm t},\perp}|H|\psi_{{\rm t},\perp}\rangle = \langle \psi_{{\rm t},\perp}|H_{\rm t}|\psi_{{\rm t},\perp}\rangle + \langle \psi_{{\rm t},\perp}|\delta H_{\rm t}|\psi_{{\rm t},\perp}\rangle \ge \Delta_{\rm t} - \|\delta H_{\rm t}\| \ge \Delta - 3\|\delta H_{\rm t}\|, \tag{71}$$

where we use $\langle \psi_{{\rm t},\perp}|H_t|\psi_{{\rm t},\perp}\rangle \ge \Delta_{\rm t}$ and Ineq. (58) in the first and second inequalities, respectively. Finally, we have

$$f_0 = \langle 0_{\rm t}|H|0_{\rm t}\rangle = \langle 0_{\rm t}|\delta H_{\rm t}|0_{\rm t}\rangle \le \|\delta H_{\rm t}\|. \tag{72}$$

By combining the above three inequalities (70), (71) and (72) with (69), we obtain the main inequality (59).

Finally, under the assumption of $\Delta > 4\|\delta H_{\rm t}\|$, the inequalities (71) and (72) ensures $f_\perp > f_0$ which we have utilized in (65):

$$f_\perp - f_0 \ge (\Delta - 3\|\delta H_{\rm t}\|) - \|\delta H_{\rm t}\| = \Delta - 4\|\delta H_{\rm t}\| > 0. \tag{73}$$

This completes the proof. □

C. Convenient lemmas on the Schmidt rank

We here show several convenient lemmas on the Schmidt rank.

Lemma 5.
i) For arbitrary quantum state $|\psi\rangle$ and operator O, the Schmidt rank of $O|\psi\rangle$ is bounded from above by

$$\mathrm{SR}(O|\psi\rangle, X) \le \mathrm{SR}(O, X)\mathrm{SR}(|\psi\rangle, X). \tag{74}$$

ii) For arbitrary two operators O_1 and O_2, the Schmidt rank $\mathrm{SR}(O_1O_2, X)$ and $\mathrm{SR}(O_1 + O_2, X)$ are bounded from above by

$$\mathrm{SR}(O_1O_2, X) \le \mathrm{SR}(O_1, X)\mathrm{SR}(O_2, X) \quad \text{and} \quad \mathrm{SR}(O_1 + O_2, X) \le \mathrm{SR}(O_1, X) + \mathrm{SR}(O_2, X), \tag{75}$$

respectively.
iii) For an arbitrary decomposition of $X = Y \sqcup Z$, the Schmidt rank $\mathrm{SR}(O, Y)$ is bounded from above by

$$\mathrm{SR}(O, Y) \le d^{2|Z|}\mathrm{SR}(O, X). \tag{76}$$

iv) For arbitrary operator O_Y which is supported on the subset Y, the Schmidt rank of $\mathrm{SR}(O_Y, X)$ is bounded from above by

$$\mathrm{SR}(O_Y, X) \le d^{|Y|} \tag{77}$$

for $\forall X \subseteq \Lambda$.
v) If an operator O_Y is supported on $Y \subseteq X$ (or $Y \subseteq X^{\mathrm{c}}$), the Schmidt rank $\mathrm{SR}(O_Y, X)$ is equal to 1:

$$\mathrm{SR}(O_Y, X) = 1 \quad \textit{for} \quad Y \subseteq X \quad \text{or} \quad Y \subseteq X^{\mathrm{c}}. \tag{78}$$

Lemma 5 is immediately derived from the definition.

On the Schmidt rank of the Hamiltonian (1), we prove the following lemma.

Lemma 6. *Let us define $L_s := \bigcup_{j=0}^{s} B_j$. Then, an interaction term H_s of H_{t} in Eq. (47) satisfies*

$$\mathrm{SR}(H_s, L_s) \le (2dl)^k. \tag{79}$$

Proof of Lemma 6. We first recall the definitions of $\{H_s\}_{s=0}^{q}$: $H_0 = h_{s=0} + h_{s=1} + h_{s=0,s=1}$ and $H_s = h_{s+1} + h_{s,s+1}$ ($s \ge 1$). From Eq. (78), we immediately obtain

$$\mathrm{SR}(h_s, L_s) = 1 \tag{80}$$

for $0 \le s \le q$. Also, the inequality (77) implies

$$\mathrm{SR}(h_Z, L_s) \le d^{|Z|} \le d^k \tag{81}$$

for arbitrary interaction terms $\{h_Z\}_{Z \subset \Lambda, |Z| \le k}$. The block-block interaction $h_{s,s+1}$ contains at most

$$\sum_{j=1}^{k} \left[\binom{2l}{j} - 2\binom{l}{j} \right] \tag{82}$$

interaction terms h_Z with $Z \subseteq B_s \sqcup B_{s+1}$ and $|Z| \le k$. Therefore, we obtain

$$\mathrm{SR}(h_{s,s+1}, L_s) \le d^k \sum_{j=1}^{k} \left[\binom{2l}{j} - 2\binom{l}{k} \right] \le (2dl)^k - 2. \tag{83}$$

By combining the inequalities (80) and (83), we obtain

$$\mathrm{SR}(H_s, L_s) \le \mathrm{SR}(h_{s,s+1}, L_s) + 2 \le (2dl)^k. \tag{84}$$

This completes the proof. □

D. The Eckart-Young theorem

We here show the Eckart-Young theorem [9] without the proof. Let us consider a normalized state $|\psi\rangle$ and give its Schmidt decomposition as

$$|\psi\rangle = \sum_{m=1}^{D_\psi} \mu_m |\psi_{1,m}\rangle \otimes |\psi_{2,m}\rangle, \tag{85}$$

where $\mu_1 \ge \mu_2 \ge \mu_3 \cdots \ge \mu_{D_\psi}$, and $\{|\psi_{1,m}\rangle\}_{m=1}^{D_\psi}$ and $\{|\psi_{2,m}\rangle\}_{m=1}^{D_\psi}$ are orthonormal states, respectively. We then consider another normalized state $|\phi\rangle$ with its Schmidt rank D_ϕ and define the overlap with the state $|\psi\rangle$ as

$$\| |\phi\rangle - |\psi\rangle \|. \tag{86}$$

The Eckart-Young theorem gives the following inequality:

$$\sum_{m > D_\phi} \mu_m^2 \le \| |\phi\rangle - |\psi\rangle \|^2. \tag{87}$$

E. Overlap between the ground state and low-entangled state

We relate the AGSP operator to the overlap between the ground state $|0_{\rm t}\rangle$ and the low-entangled state. Note that we here make the AGSP operator not for $|0\rangle$ but for $|0_{\rm t}\rangle$. On this point, we can prove the following proposition:

Proposition 2. *Let $K_{\rm t}$ be an AGSP operator for $|0_{\rm t}\rangle$ with the parameters $(\delta_{K_{\rm t}}, \epsilon_{K_{\rm t}}, D_{K_{\rm t}})$. If the following inequality holds*

$$\epsilon_{K_{\rm t}}^2 D_{K_{\rm t}} \le \frac{1}{2}, \tag{88}$$

there exists a quantum state $|\psi\rangle$ with $\mathrm{SR}(|\psi\rangle) \le D_{K_{\rm t}}$ such that

$$\| |\psi\rangle - |0_{\rm t}\rangle \| \le \epsilon_{K_{\rm t}} \sqrt{2D_{K_{\rm t}}} + \delta_{K_{\rm t}}. \tag{89}$$

Proof of Proposition 2. Let $|0_{K_{\rm t}}\rangle$ be a quantum state such that $K_{\rm t}|0_{K_{\rm t}}\rangle = |0_{K_{\rm t}}\rangle$ as in Eq. (42), namely

$$\| |0_{\rm t}\rangle - |0_{K_{\rm t}}\rangle \| \le \delta_{K_{\rm t}}, \quad \|K(1 - |0_{K_{\rm t}}\rangle\langle 0_{K_{\rm t}}|)\| \le \epsilon_{K_{\rm t}}, \quad \text{and} \quad \mathrm{SR}(K_{\rm t}) \le D_{K_{\rm t}}. \tag{90}$$

We then expand the state $|0_{K_{\rm t}}\rangle$ by the use of the Schmidt decomposition with respect to the partition $\Lambda = L \sqcup R$:

$$|0_{K_{\rm t}}\rangle = \sum_{m\ge 1} \mu_{K_{\rm t},m} |\mathcal{P}_{K_{\rm t},m}\rangle, \tag{91}$$

where the Schmidt coefficients $\{\mu_{K_{\rm t},m}\}$ are positive real numbers and defined in non-ascending order as $\mu_{K_{\rm t},1} \ge \mu_{K_{\rm t},2} \ge \mu_{K_{\rm t},3} \cdots$. Each of $\{|\mathcal{P}_{K_{\rm t},m}\rangle\}$ is a product state with respect to the partition $\Lambda = L \sqcup R$ (see Supplementary Figure 2). Hence, for an arbitrary product state $|\mathcal{P}\rangle$, the overlap with $|0_{K_{\rm t}}\rangle$ is smaller than $\langle 0_{K_{\rm t}}|\mathcal{P}_{K_{\rm t},1}\rangle = \mu_{K_{\rm t},1}$:

$$|\langle \mathcal{P}|0_{K_{\rm t}}\rangle| \le \mu_{K_{\rm t},1}. \tag{92}$$

Let us choose the target state $|\psi\rangle$ in the inequality (89) as

$$|\psi\rangle = \frac{K_{\rm t}|\mathcal{P}_{K_{\rm t},1}\rangle}{\|K_{\rm t}|\mathcal{P}_{K_{\rm t},1}\rangle\|}. \tag{93}$$

Then, our task is to upper-bound the following quantity $\Gamma_{K_{\rm t}}$:

$$\Gamma_{K_{\rm t}} := \left\| |0_{\rm t}\rangle - \frac{K_{\rm t}|\mathcal{P}_{K_{\rm t},1}\rangle}{\|K_{\rm t}|\mathcal{P}_{K_{\rm t},1}\rangle\|} \right\|. \tag{94}$$

The value of $\Gamma_{K_{\rm t}}$ is bounded from above as follows. First, from the triangle inequality, we obtain

$$\begin{aligned} \Gamma_{K_{\rm t}} = \left\| |0_{\rm t}\rangle - \frac{K_{\rm t}|\mathcal{P}_{K_{\rm t},1}\rangle}{\|K_{\rm t}|\mathcal{P}_{K_{\rm t},1}\rangle\|} \right\| &\le \left\| |0_{K_{\rm t}}\rangle - \frac{K_{\rm t}|\mathcal{P}_{K_{\rm t},1}\rangle}{\|K_{\rm t}|\mathcal{P}_{K_{\rm t},1}\rangle\|} \right\| + \| |0_{\rm t}\rangle - |0_{K_{\rm t}}\rangle \| \\ &\le \left\| |0_{K_{\rm t}}\rangle - \frac{K_{\rm t}|\mathcal{P}_{K_{\rm t},1}\rangle}{\|K_{\rm t}|\mathcal{P}_{K_{\rm t},1}\rangle\|} \right\| + \delta_{K_{\rm t}}, \end{aligned} \tag{95}$$

where the last inequality is given by the inequality (90).

Second, we prove the inequality of

$$\left\| |0_{K_{\rm t}}\rangle - \frac{K_{\rm t}|\mathcal{P}_{K_{\rm t},1}\rangle}{\|K_{\rm t}|\mathcal{P}_{K_{\rm t},1}\rangle\|} \right\| \le \frac{\epsilon_{K_{\rm t}}}{\mu_{K_{\rm t},1}}, \tag{96}$$

which reduces the inequality (95) to

$$\Gamma_{K_{\rm t}} \le \frac{\epsilon_{K_{\rm t}}}{\mu_{K_{\rm t},1}} + \delta_{K_{\rm t}}. \tag{97}$$

In order to derive the inequality (96), we express the product state $|\mathcal{P}_{K_{\rm t},1}\rangle$ in the definition (91) as

$$|\mathcal{P}_{K_{\rm t},1}\rangle = \mu_{K_{\rm t},1}|0_{K_{\rm t}}\rangle + \sqrt{1-\mu_{K_{\rm t},1}^2}\,|\psi_{K_{\rm t},\perp}\rangle, \tag{98}$$

where $|\psi_{K_{\rm t},\perp}\rangle$ is a state orthogonal to $|0_{K_{\rm t}}\rangle$. Note that $\mu_{K_{\rm t},1} \in \mathbb{R}^+$ from the definition of the Schmidt decomposition. From $K_{\rm t}|0_{K_{\rm t}}\rangle = |0_{K_{\rm t}}\rangle$, we have

$$\begin{aligned} &K_{\rm t}^2|\mathcal{P}_{K_{\rm t},1}\rangle = \mu_{K_{\rm t},1}|0_{K_{\rm t}}\rangle + \sqrt{1-\mu_{K_{\rm t},1}^2}\,K_{\rm t}^2|\psi_{K_{\rm t},\perp}\rangle, \\ &\|K_{\rm t}|\mathcal{P}_{K_{\rm t},1}\rangle\|^2 = \langle \mathcal{P}_{K_{\rm t},1}|K_{\rm t}^2|\mathcal{P}_{K_{\rm t},1}\rangle = \mu_{K_{\rm t},1}^2 + (1-\mu_{K_{\rm t},1}^2)\langle \psi_{K_{\rm t},\perp}|K_{\rm t}^2|\psi_{K_{\rm t},\perp}\rangle, \end{aligned} \tag{99}$$

where we use $\langle 0_{K_t}|K_t^2|\psi_{K_t,\perp}\rangle = \langle 0_{K_t}|\psi_{K_t,\perp}\rangle = 0$ in the second equation.

From the above equation, we obtain

$$\left\| |0_{K_t}\rangle - \frac{K_t|\mathcal{P}_{K_t,1}\rangle}{\|K_t|\mathcal{P}_{K_t,1}\rangle\|}\right\|^2 = \left\| |0_{K_t}\rangle - \frac{\mu_{K_t,1}|0_{K_t}\rangle + \sqrt{1-\mu_{K_t,1}^2}K_t|\psi_{K_t,\perp}\rangle}{\|K_t|\mathcal{P}_{K_t,1}\rangle\|}\right\|^2$$
$$= \frac{(\|K_t|\mathcal{P}_{K_t,1}\rangle\| - \mu_{K_t,1})^2 + (1-\mu_{K_t,1}^2)\langle\psi_{K_t,\perp}|K_t^2|\psi_{K_t,\perp}\rangle}{\|K_t|\mathcal{P}_{K_t,1}\rangle\|^2}$$
$$= \frac{2\|K_t|\mathcal{P}_{K_t,1}\rangle\|^2 - 2\mu_{K_t,1}\|K_t|\mathcal{P}_{K_t,1}\rangle\|}{\|K_t|\mathcal{P}_{K_t,1}\rangle\|^2}$$
$$= 2 - 2\left(1 + \frac{1-\mu_{K_t,1}^2}{\mu_{K_t,1}^2}\langle\psi_{K_t,\perp}|K_t^2|\psi_{K_t,\perp}\rangle\right)^{-1/2}, \tag{100}$$

where we use Eq. (99) in derivations of the third and the fourth equations. Then, from the inequality (43), we have $\langle\psi_{K_t,\perp}|K_t^2|\psi_{K_t,\perp}\rangle \le \epsilon_{K_t}^2$, and hence

$$\left\| |0_{K_t}\rangle - \frac{K_t|\mathcal{P}_{K_t,1}\rangle}{\|K_t|\mathcal{P}_{K_t,1}\rangle\|}\right\|^2 = 2 - 2\left(1 + \frac{1-\mu_{K_t,1}^2}{\mu_{K_t,1}^2}\langle\psi_{K_t,\perp}|K_t^2|\psi_{K_t,\perp}\rangle\right)^{-1/2}$$
$$\le \frac{1-\mu_{K_t,1}^2}{\mu_{K_t,1}^2}\langle\psi_{K_t,\perp}|K_t^2|\psi_{K_t,\perp}\rangle \le \frac{\epsilon_{K_t}^2}{\mu_{K_t,1}^2}, \tag{101}$$

where we use $(1+x)^{-1/2} \ge 1 - x/2$ for $x \ge 0$ in the first inequality. We thus obtain the inequality (96), and hence the inequality (97) is also proven.

To finish the proof, we need to derive a relationship between the coefficient $\mu_{K_t,1}$ and the AGSP parameters $\{\delta_{K_t}, \epsilon_{K_t}, D_{K_t}\}$. It allows us to obtain the upper bound of Γ_{K_t} in Eq. (94) only by the AGSP parameters. For the purpose, we here utilize the following statement called *the bootstrapping lemma*;

Lemma 7 (Bootstrapping lemma [2]). *If the AGSP operator K_t satisfies $\epsilon_{K_t}^2 D_{K_t} \le 1/2$, $\mu_{K_t,1}$ is bounded from below by*

$$\mu_{K_t,1} \ge \frac{1}{\sqrt{2D_{K_t}}}, \tag{102}$$

where $\mu_{K_t,1}$ was defined in Eq. (91).

By combining the inequalities (102) and (97), we have

$$\Gamma_{K_t} \le \frac{\epsilon_{K_t}}{\mu_{K_t,1}} + \delta_{K_t} \le \epsilon_{K_t}\sqrt{2D_{K_t}} + \delta_{K_t}. \tag{103}$$

This completes the proof of Proposition 2.

1. *Proof of Lemma 7*

We first denote the Schmidt decomposition of $K_t|\mathcal{P}_{K_t,1}\rangle$ by

$$K_t|\mathcal{P}_{K_t,1}\rangle = \sum_{j=1}^{D_{K_t}} \mu'_{K_t,j}|\mathcal{P}'_j\rangle. \tag{104}$$

Note that $K_t|\mathcal{P}_{K_t,1}\rangle$ is not normalized. We obtain

$$\langle 0_{K_t}|K_t|\mathcal{P}_{K_t,1}\rangle = \sum_{j=1}^{D_{K_t}} \mu'_{K_t,j}\langle 0_{K_t}|\mathcal{P}'_j\rangle \le \sqrt{\sum_{j=1}^{D_{K_t}}\mu'^2_{K_t,j}}\sqrt{\sum_{j=1}^{D_{K_t}}|\langle 0_{K_t}|\mathcal{P}'_j\rangle|^2} = \|K_t|\mathcal{P}_{K_t,1}\rangle\|\sqrt{\sum_{j=1}^{D_{K_t}}|\langle 0_{K_t}|\mathcal{P}'_j\rangle|^2}, \tag{105}$$

where the first inequality is given by the Cauchy-Schwartz inequality. We now have

$$\langle 0_{K_t}|K_t|\mathcal{P}_{K_t,1}\rangle = \langle 0_{K_t}|\mathcal{P}_{K_t,1}\rangle = \mu_{K_t,1},$$
$$\sum_{j=1}^{D_{K_t}}|\langle 0_{K_t}|\mathcal{P}'_j\rangle|^2 \le \sum_{j=1}^{D_{K_t}}\mu_{K_t,1}^2 = D_{K_t}\mu_{K_t,1}^2,$$
$$\|K_t|\mathcal{P}_{K_t,1}\rangle\| \le \sqrt{\mu_{K_t,1}^2 + \epsilon_{K_t}^2}, \tag{106}$$

where the first equation is given by the definition $K_{\rm t}|0_{K_{\rm t}}\rangle = |0_{K_{\rm t}}\rangle$, the second inequality is derived from Ineq. (92), and the third inequality is derived from Ineq. (99) with $\langle\psi_{K_{\rm t},\perp}|K_{\rm t}^2|\psi_{K_{\rm t},\perp}\rangle \le \epsilon_{K_{\rm t}}^2$. Thus, the inequality (105) reduces to

$$\mu_{K_{\rm t},1} \le \sqrt{\mu_{K_{\rm t},1}^2 + \epsilon_{K_{\rm t}}^2}\sqrt{D_{K_{\rm t}}\mu_{K_{\rm t},1}^2}, \tag{107}$$

which gives the inequality

$$\mu_{K_{\rm t},1}^2 \ge \frac{1}{D_{K_{\rm t}}} - \epsilon_{K_{\rm t}}^2 = \frac{1-D_{K_{\rm t}}\epsilon_{K_{\rm t}}^2}{D_{K_{\rm t}}} \ge \frac{1}{2D_{K_{\rm t}}}, \tag{108}$$

where we utilized $D_{K_{\rm t}}\epsilon_{K_{\rm t}}^2 \le 1/2$. This completes the proof. □

F. Upper bound of the entanglement entropy by the AGSP operators

We here relate the AGSP operator to the ground-state entropy $S(|0\rangle)$. For this purpose, we make a sequence of the AGSP operators $\{K_p\}_{p=1}^\infty$ for $|0\rangle$ each of which has a state $|0_{K_p}\rangle$ such that $K_p|0_{K_p}\rangle = |0_{K_p}\rangle$ with Eq. (42). For simplicity, we denote $\{\delta_{K_p}, \epsilon_{K_p}, D_{K_p}\}$ by $\{\delta_p, \epsilon_p, D_p\}$. We choose K_p so that K_∞ may satisfy $\epsilon_\infty = 0$, $\delta_\infty = 0$; in other words, K_∞ is the exact ground-state projector. We denote the exact ground state $|0\rangle = |0_\infty\rangle$ by

$$|0\rangle = \sum_{m=1}^{D_\infty} \mu_m |\mathcal{P}_m\rangle. \tag{109}$$

Note that because of $K_\infty = |0\rangle\langle 0|$ the Schmidt rank of $|0\rangle$ is equal to D_∞.

We now obtain the following proposition:

Proposition 3. *Let $|\psi_D\rangle$ be an arbitrary quantum state with*

$$\||\psi_D\rangle - |0\rangle\| = \nu_0 \quad \text{and} \quad \mathrm{SR}(|\psi_D\rangle) = D. \tag{110}$$

Also, we define $\{K_p\}_{p=1}^\infty$ as $(\{\delta_p, \epsilon_p, D_p\}_{p=1}^\infty)$-AGSP operators, respectively, where errors ϵ_p and δ_p decrease with the index p, namely $\epsilon_1 \ge \epsilon_2 \ge \cdots$ and $\delta_1 \ge \delta_2 \ge \cdots$. Then, we prove for each of $\{K_p\}_{p=1}^\infty$

$$\left\| \frac{K_p e^{-i\theta_p}|\psi_D\rangle}{\|K_p|\psi_D\rangle\|} - |0\rangle \right\| \le \gamma_p \tag{111}$$

with $\theta_p \in \mathbb{R}$ given in Eq. (114), where $\{\gamma_p\}_{p=1}^\infty$ are defined as

$$\gamma_p := \frac{\epsilon_p}{1-\nu_0-\delta_p} + \delta_p. \tag{112}$$

Moreover, under the condition $\gamma_p \le 1$ for all p, the entanglement entropy $S(E_0)$ is bounded from above by

$$S(|0\rangle) \le \log(D) - \sum_{p=0}^{\infty} \gamma_p^2 \log\frac{\gamma_p^2}{3D_{p+1}}, \tag{113}$$

where we set $\gamma_0 := 1$.

Proof of Proposition 3. For the proof, we construct an approximate ground state by means of $K_p e^{-i\theta_p}|\psi_D\rangle$ with $e^{-i\theta_p}$ an appropriate phase factor such that

$$\langle 0_{K_p}|e^{-i\theta_p}|\psi_D\rangle = |\langle 0_{K_p}|\psi_D\rangle|. \tag{114}$$

We now want to know how close it is to the exact ground state $|0\rangle$. We first define the Schmidt rank of $K_p e^{-i\theta_p}|\psi_D\rangle$ by D'_p which is smaller than DD_p:

$$D'_p := \mathrm{SR}(K_p e^{-i\theta_p}|\psi_D\rangle) = \mathrm{SR}(K_p|\psi_D\rangle) \le \mathrm{SR}(|\psi_D\rangle)\mathrm{SR}(K_p) = DD_p. \tag{115}$$

Second, we apply the Eckart-Young theorem by letting $|\psi\rangle = |0\rangle$, $|\phi\rangle = \frac{K_p e^{-i\theta_p}|\psi_D\rangle}{\|K_p|\psi_D\rangle\|}$ and $D_\phi = D'_p$ in (87):

$$\sum_{m>D'_p} \mu_m^2 \le \left\| \frac{K_p e^{-i\theta_p}|\psi_D\rangle}{\|K_p|\psi_D\rangle\|} - |0\rangle \right\|^2 =: \Gamma_p^2, \tag{116}$$

where the Schmidt decomposition for the ground state has been given in Eq. (109). Therefore, in order to derive the inequality (111), we need to prove $\Gamma_p \le \gamma_p$ with γ_p defined in Eq. (112).

In order to upper-bound Γ_p by using the AGSP parameters ($\{\delta_p, \epsilon_p, D_p\}_{p=1}^{\infty}$), we start from the triangle inequality as follows:

$$\Gamma_p := \left\| |0\rangle - \frac{K_p e^{-i\theta_p}|\psi_D\rangle}{\|K_p|\psi_D\rangle\|} \right\| \le \left\| |0_{K_p}\rangle - \frac{K_p e^{-i\theta_p}|\psi_D\rangle}{\|K_p|\psi_D\rangle\|} \right\| + \| |0_{K_p}\rangle - |0\rangle \| \\ \le \left\| |0_{K_p}\rangle - \frac{K_p e^{-i\theta_p}|\psi_D\rangle}{\|K_p|\psi_D\rangle\|} \right\| + \delta_p, \tag{117}$$

where the last inequality is derived from the definition of the AGSP parameter as in the inequality (42). We, in the following, derive the upper bound of the first term in (117). By using Eq. (114), we decompose the quantum state $e^{-i\theta_p}|\psi_D\rangle$ by

$$e^{-i\theta_p}|\psi_D\rangle = \nu_p |0_{K_p}\rangle + \sqrt{1-|\nu_p|^2}|\psi_{p,\perp}\rangle \quad \text{with} \quad \nu_p := |\langle 0_{K_p}|\psi_D\rangle|, \tag{118}$$

where $|\psi_{p,\perp}\rangle$ is a state orthogonal to $|0_{K_p}\rangle$. We then derive the upper bound of ν_p, which is given by

$$\nu_p = |(\langle 0| + \langle 0_{K_p}| - \langle 0|)|\psi_D\rangle)| \ge |\langle 0|\psi_D\rangle| - \| |0_{K_p}\rangle - |0\rangle \| \ge 1 - \nu_0 - \delta_p, \tag{119}$$

where ν_0 has been defined in Eq. (110) and we use $|\langle 0|\psi_D\rangle| \ge 1 - \| |\psi_D\rangle - |0\rangle \| = 1 - \nu_0$ in the last inequality. We then follow the same steps as the derivations of Ineq. (99), (100) and (101); in these inequalities, we replace as

$$|\mathcal{P}_{K_t,1}\rangle \to |\psi_D\rangle, \quad |0_{K_t}\rangle \to |0_{K_p}\rangle, \quad \mu_{K_t,1} \to \nu_p. \tag{120}$$

Thus, we obtain

$$\left\| |0_{K_p}\rangle - \frac{K_p e^{-i\theta_p}|\psi_D\rangle}{\|K_p|\psi_D\rangle\|} \right\| \le \frac{\epsilon_p}{\nu_p} \le \frac{\epsilon_p}{1-\nu_0-\delta_p}, \tag{121}$$

where we use (119) in the second inequality. By combining the inequalities (117) and (121), we obtain $\Gamma_p \le \gamma_p$.

The remaining task is to upper-bound the entanglement entropy to derive the inequality (113). We first define

$$\Gamma_{p,p+1}^2 := \sum_{D'_p < m \le D'_{p+1}} \mu_m^2, \tag{122}$$

where we define $D'_0 = 0$. Note that from Eq. (109) we have

$$\sum_{p=0}^{\infty} \Gamma_{p,p+1}^2 = \sum_{0<m\le D'_\infty} \mu_m^2 = \sum_{m=1}^{D_\infty} \mu_m^2 = 1. \tag{123}$$

From the inequality (116), we have

$$\Gamma_{p,p+1}^2 \le \Gamma_p^2 \le \gamma_p^2 \le 1, \tag{124}$$

where the last inequality is given by the condition in the proposition. From the above definition, we have

$$- \sum_{D'_p < m \le D'_{p+1}} \mu_m^2 \log(\mu_m^2) \le - \sum_{D'_p < m \le D'_{p+1}} \frac{\Gamma_{p,p+1}^2}{D'_{p+1} - D'_p} \log \frac{\Gamma_{p,p+1}^2}{D'_{p+1} - D'_p} \\ = -\Gamma_{p,p+1}^2 \log \frac{\Gamma_{p,p+1}^2}{D'_{p+1} - D'_p} \le -\Gamma_{p,p+1}^2 \log \frac{\Gamma_{p,p+1}^2}{D_{p+1}} + \Gamma_{p,p+1}^2 \log D, \tag{125}$$

where in the last inequality we use $D'_p \le D D_p$ in (115).

By using $\{\Gamma_{p,p+1}\}_{p=1}^{\infty}$ and the ineuqlaity (125), the entanglement entropy of $|0\rangle$ is bounded from above by

$$S(|0\rangle) = -\sum_{m\ge 1} \mu_m^2 \log(\mu_m^2) = -\sum_{p=0}^{\infty} \sum_{D'_p < m \le D'_{p+1}} \mu_m^2 \log(\mu_m^2) \le \log D - \sum_{p=0}^{\infty} \Gamma_{p,p+1}^2 \log \frac{\Gamma_{p,p+1}^2}{D_{p+1}}, \tag{126}$$

where we use Eq. (123) in the second equation. We have $-x\log(x/3) \le -y\log(y/3)$ for $0 < x \le y \le 1$, and hence

$$-\Gamma_{p,p+1}^2 \log \Gamma_{p,p+1}^2 \le -\Gamma_{p,p+1}^2 \log(\Gamma_{p,p+1}^2/3) \le -\gamma_p^2 \log(\gamma_p^2/3), \tag{127}$$

which reduces the inequality (126) to the main inequality (113). This completes the proof of Proposition 3. □

G. Schmidt rank of the polynomials of the truncated Hamiltonian

We first show the following lemma:

Lemma 8. *The Schmidt rank of the power of the truncated Hamiltonian* $\mathrm{SR}(H_{\mathrm{t}}^m)$ *is bounded from above by*

$$\mathrm{SR}(H_{\mathrm{t}}^m) \le [2 + (2dl)^k]^m. \tag{128}$$

Proof of Lemma 8. We first decompose H_{t} into

$$H_{\mathrm{t}} = H_{q/2} + H_{<q/2} + H_{>q/2}, \tag{129}$$

where $H_{<q/2} := \sum_{s<q/2} H_s$ and $H_{>q/2} := \sum_{s>q/2} H_s$. Note that $H_{<q/2}$ and $H_{>q/2}$ are supported on the subsystems L and R, respectively. From Lemmas 5 and 6, we have

$$\begin{aligned} &\mathrm{SR}(H_{\mathrm{t}}^m) \le [\mathrm{SR}(H_{\mathrm{t}})]^m, \\ &\mathrm{SR}(H_{q/2}) \le (2dl)^k, \quad \mathrm{SR}(H_{<q/2}) = \mathrm{SR}(H_{>q/2}) = 1, \end{aligned} \tag{130}$$

which yield the inequality (128). This completes the proof.

Roughly speaking, the inequality (128) gives the Schmidt rank of order of $\exp[\mathcal{O}(m)\log(dl)]$. In fact, when q is large, we obtain much better bound for $\mathrm{SR}(H_{\mathrm{t}}^m)$ as shown in the following proposition:

Proposition 4. *The Schmidt rank of the power of the truncated Hamiltonian* $\mathrm{SR}(H_{\mathrm{t}}^m)$ *is bounded from above by*

$$\mathrm{SR}(H_{\mathrm{t}}^m) \le d^{ql}(q+m+1)^{q+1}[e(q+1)^2(2dl)^k]^{m/(q+1)} \le d^{2ql}[e(q+1)^2(2dl)^k]^{m/(q+1)}, \tag{131}$$

where for the simplicity we assume $(q+m+1)^{q+1} \le d^{ql}$ *which yields the second inequality.*

The above estimation gives the Schmidt rank of order of $\exp[\mathcal{O}(ql)\log(d) + \mathcal{O}(m/q)\log(dl)]$.

Proof of Proposition 4. We can prove the proposition by extending the original argument in Ref. [2] to the present long-range interacting case. In order to estimate the Schmidt rank of H_{t}^m, we first describe it as

$$H_{\mathrm{t}}^m = \sum_{s_0+s_1+\cdots+s_q=m} O_{s_0,s_1,\ldots,s_q}, \tag{132}$$

where each of $\{O_{s_0,s_1,\ldots,s_q}\}$ is given by summation of the operator products in which H_i appears s_i times for $0 \le i \le q$. We here define $O_{i,s}$ as the summation of $\{O_{s_0,s_1,\ldots,s_q}\}$ such that $s_i = s$, $\min(s_0, s_1, \ldots, s_{i-1}) > s$ and $\min(s_{i+1}, s_{i+2}, \ldots, s_q) \ge s$; that is, the number of appearance of H_i is minimum. Notice that the definition of $O_{i,s}$ implies $s \le \lfloor m/(q+1) \rfloor$. Explicitly, $O_{i,s}$ is given by

$$O_{i,s} = \sum_{\substack{s_0+s_1+\cdots+s_q=m \\ s_i=s,\ \min(s_0,s_1,\ldots,s_{i-1})>s\ \ \min(s_{i+1},s_{i+2},\ldots,s_q)\ge s}} O_{s_0,s_1,\ldots,s_q}. \tag{133}$$

By using the notation of $O_{i,s}$, we obtain

$$H_{\mathrm{t}}^m = \sum_{i=0}^{q} \sum_{s=0}^{\lfloor m/(q+1) \rfloor} O_{i,s}. \tag{134}$$

From the basic property of the Schmidt rank, we obtain

$$\mathrm{SR}(H_{\mathrm{t}}^m) \le \sum_{i=0}^{q} \sum_{s=0}^{\lfloor m/(q+1) \rfloor} \mathrm{SR}(O_{i,s}). \tag{135}$$

Our task is to estimate the Schmidt rank $\mathrm{SR}(O_{i,s})$. For the purpose, instead of considering $O_{i,s}$ in itself, we consider the following alternative operator $P_{i,s}$ which depends on parameters $\vec{z} = \{z_i\}_{i=0}^q \in \mathbb{C}^{\otimes q+1}$:

$$P_{i,s}(\vec{z}) = \sum_{\substack{s_0+s_1+\cdots+s_q=m \\ s_i=s}} z_0^{s_0} z_1^{s_1} \cdots z_q^{s_q} O_{s_0,s_1,\ldots,s_q}. \tag{136}$$

We notice that $P_{i,s}(\vec{1})$ is not generally equal to $O_{i,s}$ since the conditions $\min(s_0, \ldots, s_{i-1}) > s$ and $\min(s_{i+1}, \ldots, s_q) \ge s$ are not imposed for $P_{i,s}(\vec{1})$.

In the following, we aim to express $O_{i,s}$ by using $P_{i,s}(\vec{z})$ for specific choices of $\{\vec{z}_\alpha\}_{\alpha=1}^{\mathcal{N}_s}$:

$$O_{i,s} = \sum_{\alpha=1}^{\mathcal{N}_s} \lambda_\alpha P_{i,s}(\vec{z}_\alpha), \tag{137}$$

where $\lambda_\alpha \in \mathbb{C}$ for $\alpha = 1, 2, \ldots, \mathcal{N}_s$. The equation (137) gives the upper bound of $\mathrm{SR}(O_{i,s})$ as

$$\mathrm{SR}(O_{i,s}) \le \mathcal{N}_s \sup_{\vec{z} \in \mathbb{C}^{\otimes q+1}} \mathrm{SR}[P_{i,s}(\vec{z})]. \tag{138}$$

As shown in the following lemma, the Schmidt rank of $P_{i,s}(\vec{z})$ can be efficiently estimated:

Lemma 9. *The Schmidt rank of $P_{i,s}(\vec{z})$ is bounded from above as*

$$\mathrm{SR}[P_{i,s}(\vec{z})] \le d^{ql}(m+1)[em^2(2dl)^k/s^2]^s \tag{139}$$

for $\forall \vec{z} \in \mathbb{C}^{\otimes q+1}$.

Second, we can prove the following lemma:

Lemma 10. *There exists a set of $\{\vec{z}_\alpha\}_{\alpha=1}^{\mathcal{N}_s}$ which gives Eq. (137) as long as*

$$\mathcal{N}_s = \binom{q+m-s-1}{q-1} \le (q+m)^{q-1}. \tag{140}$$

From the lemmas 9 and 10, the inequality (138) reduces to

$$\mathrm{SR}(O_{i,s}) \le (q+m)^{q-1} d^{ql}(m+1)[em^2(2dl)^k/s^2]^s, \tag{141}$$

which monotonically increases with s for $s \le m/(q+1)$. From Eq. (135) and $\lfloor m/(q+1) \rfloor \le m/(q+1)$, we have

$$\begin{aligned} \mathrm{SR}(H_{\mathrm{t}}^m) &\le \sum_{i=0}^{q} \sum_{s=0}^{\lfloor m/(q+1) \rfloor} (q+m)^{q-1} d^{ql}(m+1)[e(q+1)^2(2dl)^k]^{m/(q+1)} \\ &\le d^{ql}(q+m)^{q-1}(q+m+1)(m+1)[e(q+1)^2(2dl)^k]^{m/(q+1)} \\ &\le d^{ql}(q+m+1)^{q+1}[e(q+1)^2(2dl)^k]^{m/(q+1)}, \end{aligned} \tag{142}$$

where in the second inequality we use $(q+1)(\lfloor m/(q+1) \rfloor + 1) \le q+m+1$. This completes the proof of Proposition 4. □

1. *Proof of Lemma 9*

We first define parametrized Hamiltonian $H(\vec{z})$ as follows:

$$H(\vec{z}) = \sum_{j=0}^{q} z_j H_j =: z_i H_i + H_{\neq i}(\vec{z}), \tag{143}$$

where we define $H_{\neq i}(\vec{z}) := \sum_{j \neq i} z_j H_j$. Then, $P_{i,s}(\vec{z})$ is given by

$$P_{i,s}(\vec{z}) = \sum_{t_1+t_2+\cdots+t_{s+1}=m-s} [H_{\neq i}(\vec{z})]^{t_1}(z_iH_i)[H_{\neq i}(\vec{z})]^{t_2}(z_iH_i)\cdots[H_{\neq i}(\vec{z})]^{t_s}(z_iH_i)[H_{\neq i}(\vec{z})]^{t_{s+1}}. \tag{144}$$

We now estimate the Schmidt rank of $[H_{\neq i}(\vec{z})]^t$ and z_iH_i. The latter one has been already given by Lemma 6 as $\mathrm{SR}(z_iH_i, L_i) \le (2dl)^k$. Recall that the subset $L_i \in \Lambda$ has been defined in Lemma 6 as $L_i := \bigcup_{j=0}^{i} B_j$. In order to estimate $\mathrm{SR}([H_{\neq i}(\vec{z})]^t, L_i)$, we define $H(\vec{z})_{<i}$ and $H(\vec{z})_{>i}$ as

$$H_{<i}(\vec{z}) = \sum_{j<i} z_j H_j, \quad H_{>i}(\vec{z}) = \sum_{j>i} z_j H_j, \tag{145}$$

where $H_{<i}(\vec{z})$ and $H_{>i}(\vec{z})$ are supported on the subsets L_i and L_i^{c}, respectively. Note that $H_{\neq i}(\vec{z}) = H_{<i}(\vec{z}) + H_{>i}(\vec{z})$. We have

$$\mathrm{SR}([H_{<i}(\vec{z})]^j \otimes [H_{>i}(\vec{z})]^{t-j}, L_i) = 1 \tag{146}$$

for $\forall j$, and hence the Schmidt rank of

$$[H_{\neq i}(\vec{z})]^t = \sum_{j=0}^{t} \binom{t}{j} [H_{<i}(\vec{z})]^j [H_{>i}(\vec{z})]^{t-j} \tag{147}$$

is bounded from by

$$\mathrm{SR}([H_{\neq i}(\vec{z})]^t, L_i) \le t+1. \tag{148}$$

We thus obtain

$$\begin{aligned}
\mathrm{SR}[P_{i,s}(\vec{z}), L_i] &\le \sum_{t_1+t_2+\cdots+t_{s+1}=m-s} (2dl)^{ks} \prod_{j=1}^{s+1} (t_j+1) \\
&\le \sum_{t_1+t_2+\cdots+t_{s+1}=m-s} (2dl)^{ks} (m+1)[m/(s+1)]^{s+1} \\
&= \left(\!\!\binom{s+1}{m-s}\!\!\right) (2dl)^{ks} (m+1)[m/(s+1)]^{s+1} \le (m+1)[em^2(2dl)^k/s^2]^s,
\end{aligned} \tag{149}$$

where the summation with respect to $\{t_1, t_2, \cdots, t_{s+1}\}$ such that $t_1 + t_2 + \cdots + t_{s+1} = m - s$ is equal to the $(m-s)$-multicombination from a set of $s+1$ elements, and in the inequality, we use

$$\begin{aligned}
&\prod_{j=1}^{s+1} (t_j+1) \le \left(\frac{m+1}{s+1}\right)^{s+1} \le (m+1)(m/s)^s, \\
&\left(\!\!\binom{s+1}{m-s}\!\!\right) = \binom{m}{s} \le (em/s)^s
\end{aligned} \tag{150}$$

for $t_1 + t_2 + \cdots + t_{s+1} = m - s$. Finally, by applying the inequality (76) to (149), we obtain the inequality (139). Note that $\max(|L_i \setminus L|, |L \setminus L_i|) \le ql/2$ for $0 \le i \le q$. This completes the proof. □

2. *Proof of Lemma 10*

For the proof, we first choose each of $\{z_i\}_{i=0}^q$ as $z_i = x^{(m+1)^i}$ with x a parameter which is fixed afterward. It reduces Eq. (136) to

$$P_{i,s}(x) = \sum_{\substack{s_0+s_1+\cdots+s_q=m \\ s_i=s}} x^{d(\vec{s})} O_{\vec{s}} \tag{151}$$

with $d(\vec{s}) := \sum_{i=0}^q s_i (m+1)^i$, where $\vec{s} = \{s_j\}_{j=0}^q$ and $O_{\vec{s}} := O_{s_0,s_1,\ldots,s_q}$. We notice that we have $d(\vec{s}) \neq d(\vec{s}')$ if $\vec{s} \neq \vec{s}'$ because of $s_j \le m$ for $\forall j$.

We label different $\vec{s} = \{s_j\}_{j=0}^q$ such that $s_0 + s_1 + \cdots + s_q = m$ with $s_i = s$ by $\{\vec{s}_u\}_{u=1}^{\mathcal{N}}$, where the total number $\mathcal{N}$ is equal to the $(m-s)$-multicombination from a set of q elements:

$$\mathcal{N} = \left(\!\!\binom{q}{m-s}\!\!\right) = \binom{q+m-s-1}{m-s} = \mathcal{N}_s. \tag{152}$$

We also order $\{\vec{s}_u\}_{u=1}^{\mathcal{N}}$ so that $d(\vec{s}_1) > d(\vec{s}_2) > \cdots > d(\vec{s}_{\mathcal{N}_s}) > 0$. In this notation, Eq. (151) reduces to

$$P_{i,s}(x) = \sum_{u=1}^{\mathcal{N}_s} x^{d(u)} O_u, \tag{153}$$

where $d(u) := d(\vec{s}_u)$ and $O_u := O_{\vec{s}_u}$. Therefore, if there exists a set of $\{x_v\}_{v=1}^{\mathcal{N}_s}$ such that the matrix

$$M = \begin{pmatrix} x_1^{d(1)} & x_1^{d(2)} & \cdots & x_1^{d(\mathcal{N}_s)} \\ x_2^{d(1)} & x_2^{d(2)} & \cdots & x_2^{d(\mathcal{N}_s)} \\ \vdots & \vdots & \cdots & \vdots \\ x_{\mathcal{N}_s}^{d(1)} & x_{\mathcal{N}_s}^{d(2)} & \cdots & x_{\mathcal{N}_s}^{d(\mathcal{N}_s)} \end{pmatrix} \tag{154}$$

has full rank, an arbitrary O_u is described by

$$O_u = \sum_{v=1}^{\mathcal{N}_s} \lambda_{u,v} P_{i,s}(x_v). \tag{155}$$

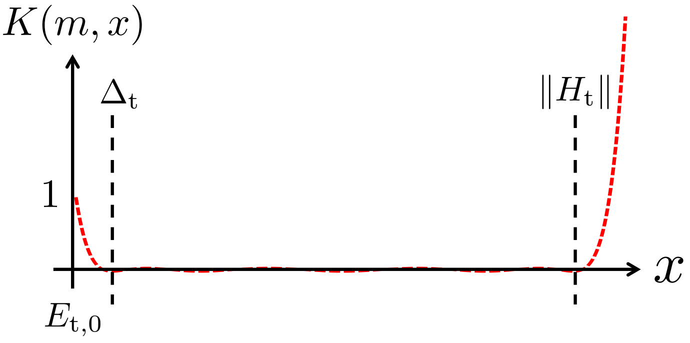

Supplementary Figure 7. By the use of the Chebyshev polynomials, we can construct a function $K(m,x)$ which approximately satisfies $K(m,0)=1$ and $K(m,x)\simeq 0$ for $\Delta_{\rm t}\le x\le \|H_{\rm t}\|$. The error of the approximation is bounded from above as in (164).

Now, $\lambda_{u,v}$ is given by $\lambda_{u,v}=(M^{-1})_{u,v}$ with M^{-1} the inverse matrix of M.

In order to show the full rank of M, we prove $\det(M)\neq 0$ for a particular choice of $\{x_v\}_{v=1}^{\mathcal{N}_s}$. Because of $d(1)>d(2)>\cdots>d(\mathcal{N}_s)$, $\det(M)$ is equal to the product of the Vandermonde's determinant and the Schur polynomial. The former one is given by $\prod_{v<v'}(x_v-x_{v'})$ and is non-zero as long as $x_v\neq x_{v'}$ for $v\neq v'$. Moreover, the latter one is positive if $x_v>0$ for $\forall v$ since the Schur polynomial is composed of monomials with positive coefficients [10, 11]. Hence, if we choose $\{x_v\}_{v=1}^{\mathcal{N}}$ such that $x_v>0$ for $\forall v$ and $x_v\neq x_{v'}$ for $v\neq v'$, we have $\det(M)\neq 0$.

This completes the proof of Lemma 10. □

H. Construction of the AGSP

We here discuss how we can find the AGSP operator satisfying (88). In order to construct the AGSP, we utilize a polynomial of the Hamiltonian like Poly($H_{\rm t}$). For example, one of the candidates for AGSP $\{K_p\}_{p=1}^{\infty}$ is given by $K_p=\left(1-\frac{H_{\rm t}}{\|H_{\rm t}\|}\right)^p$. In this case, ϵ_{K_p} in Ineq. (42) is upper-bounded by $e^{-p\Delta/\|H_{\rm t}\|}$, and we thereby obtain the exact ground-state projection in the limit of $p\to\infty$. However, this AGSP cannot satisfy the condition $D_p\epsilon_p^2\le 1/2$ of the bootstrapping lemma. For the proof of the area law, we need to construct an AGSP operator with a higher accuracy and a lower Schmidt rank.

For this purpose, we first define an mth-order polynomial $K(m,x)$ such that $K(m,0)=1$ and

$$|K(m,x)|\le \epsilon_m \tag{156}$$

for $\Delta_{\rm t}\le x\le \|H_{\rm t}\|$ with ϵ_m a positive number (see Supplementary Figure 7 for the schematic picture). From the definition $E_{{\rm t},0}=0$, this polynomial gives

$$\|K(m,H_{\rm t})-|0_{\rm t}\rangle\langle 0_{\rm t}|\|\le \epsilon_m. \tag{157}$$

In the construction of the polynomial $K(m,x)$, we employ the Chebyshev polynomial [2, 5, 6]:

$$T_m(x):=\frac{\left(x+\sqrt{x^2-1}\right)^m+\left(x-\sqrt{x^2-1}\right)^m}{2}. \tag{158}$$

The first few polynomials are given by

$$\begin{aligned} &T_1(x)=x,\quad T_2(x)=2x^2-1,\quad T_3(x)=4x^3-3x,\\ &T_4(x)=8x^4-8x^2+1,\quad T_5(x)=16x^5-20x^3+5x. \end{aligned} \tag{159}$$

As shown in the following lemma, the Chebyshev polynomial $T_m(x)$ approximately behaves as a boxcar function in the range $[-1,1]$.

Lemma 11 (Lemma B.2 in Kuwahara, Arad, Amico and Vedral [6]). *The Chebyshev polynomial $T_m(x)$ satisfies*

$$|T_m(x)|\le 1 \quad \text{for} \quad |x|\le 1, \tag{160}$$

$$\frac{1}{2}\exp\left(2m\sqrt{\frac{|x|-1}{|x|+1}}\right)\le |T_m(x)|\le \frac{(2x)^m}{2} \quad \text{for} \quad |x|\ge 1. \tag{161}$$

We now choose $K(m,x)$ as follows:

$$K(m,x) = \frac{T_m\left[\frac{2x-(\|H_t\|+\Delta_t)}{\|H_t\|-\Delta_t}\right]}{T_m\left[-\frac{\|H_t\|+\Delta_t}{\|H_t\|-\Delta_t}\right]}, \tag{162}$$

$$\frac{2x-(\|H_t\|+\Delta_t)}{\|H_t\|-\Delta_t}\begin{cases} = -1 & \text{for} \quad x = \Delta_t, \\ \in (-1,1) & \text{for} \quad \Delta_t < x < \|H_t\|, \\ = 1 & \text{for} \quad x = \|H_t\|, \end{cases}$$

where $K(m,0) = 1$ and the lemma 11 implies

$$T_m\left[-\frac{\|H_t\|+\Delta_t}{\|H_t\|-\Delta_t}\right] \geq \frac{1}{2}\exp\left(2m\sqrt{\frac{\Delta_t}{\|H_t\|}}\right). \tag{163}$$

Thus, ϵ_m in the inequality (156) is upper-bounded by

$$\epsilon_m = \sup_{\Delta_t \leq x \leq \|H_t\|} |K(m,x)| \leq \frac{1}{T_m\left[-\frac{\|H_t\|+\Delta_t}{\|H_t\|-\Delta_t}\right]} \leq 2e^{-2m\sqrt{\Delta_t/\|H_t\|}}. \tag{164}$$

We therefore conclude that the error of the AGSP $K(m,H_t)$ decreases as $e^{-2m\sqrt{\Delta_t/\mathcal{O}(n)}}$ because of $\|H_t\| = \mathcal{O}(n)$. However, in this case, we have to take m as large as $\mathcal{O}(\sqrt{n})$ for a good approximation, which may result in a high Schmidt rank of the AGSP operator. We thus need to achieve a good approximation with smaller m. We thereby consider an effective Hamiltonian instead of the original Hamiltonian.

I. Effective Hamiltonian with a small norm

In order to construct the AGSP operator that satisfies the condition (88) for the bootstrapping lemma, it is convenient to utilize an effective Hamiltonian $\tilde{H}_t$ instead of the original Hamiltonian H_t. Here, the effective Hamiltonian $\tilde{H}_t$ has almost the same ground state as the original one. The points are the followings:

1. The effective Hamiltonian has the norm much smaller than that of the original Hamiltonian, namely $\|\tilde{H}_t\| \ll \|H_t\|$.
2. The Schmidt rank of $\tilde{H}_t^m$ should be as small as that of H_t^m. This condition implies that the effective Hamiltonian $\tilde{H}_t$ should still have the similar locality to the original one H_t.

Note that because of the inequality (164) the norm of the Hamiltonian critically determines the error of the AGSP operators. By applying the Chebyshev-based AGSP construction (162) to the effective Hamiltonian, we can construct the AGSP $K(m,\tilde{H}_t)$ which satisfies the condition (88).

We, in the following, analyze the fundamental property of the effective Hamiltonian given in Eq. (50) (see also Supplementary Figure 3):

$$\tilde{H}_t = \sum_{s=0}^{q+1}\tilde{h}_s + \sum_{s=0}^{q} h_{s,s+1} \tag{165}$$

with

$$\tilde{h}_s = \sum_{E_{s,j}<\tau_s} E_{s,j}|E_{s,j}\rangle\langle E_{s,j}| + \sum_{E_{s,j}\geq\tau_s} \tau_s|E_{s,j}\rangle\langle E_{s,j}|,$$
$$\tau_s = E_{s,0} + \tau, \tag{166}$$

where $\{E_{s,j}, |E_{s,j}\rangle\}_{j\geq 0}$ are the eigenvalues and the eigenstates of h_s, respectively.

We first notice that Lemma 8 and Proposition 4 on the Schmidt rank are applicable to the effective Hamiltonian $\tilde{H}_t$.

From the definition (166), we immediately obtain $\|\tilde{h}_s\| \leq \tau$. Also, the inequality (46) gives $\|h_{s,s+1}\| \leq g_0$, and hence the norm of the effective Hamiltonian$\|\tilde{H}_t\|$ is upper-bounded by

$$\|\tilde{H}_t\| \leq \sum_{s=0}^{q+1}\|\tilde{h}_s\| + \sum_{s=0}^{q}\|h_{s,s+1}\| \leq \sum_{s=0}^{q+1}(\tau + |E_{s,0}|) + (q+1)g_0. \tag{167}$$

The ground-state energy $|E_{s,0}|$ can have a value of $\mathcal{O}(l)$, which gives the upper bound of $\|\tilde{H}_t\| \leq \mathcal{O}(ql) + \mathcal{O}(q\tau)$. However, by appropriately shifting each of the energy origins of $\{h_s\}_{s=0}^{q+1}$ as $\{h_s + \mathcal{E}_s\}_{s=0}^{q+1}$, we can achieve $|E_{s,0} + \mathcal{E}_s| = \mathcal{O}(1)$ for $s = 0, 1, 2, \ldots, q+1$.

One can obtain the following lemma (see Supplementary Note 2 I 1 for the proof).

Lemma 12. *There exists an energy shift from* $\{h_s\}_{s=0}^{q+1}$ *to* $\{h_s + \mathcal{E}_s\}_{s=0}^{q+1}$ *such that*

$$\sum_{s=0}^{q+1} \mathcal{E}_s = 0, \tag{168}$$

and the absolute values of the ground-state energy $\{E_{s,0}\}_{s=0}^{q+1}$ *are bounded from above by*

$$|E_{s,0} + \mathcal{E}_s| \le \frac{q+1}{q+2} g_0 \le g_0, \tag{169}$$

where g_0 *has been defined in* (15)*. Note that the condition* (168) *guarantees that* $H_{\rm t}$ *remains the same as before shifting the energies.*

By following Lemma 12, we shift the energy origin so that the inequality (169) is satisfied. We then obtain the upper bound of $\|\tilde{H}_{\rm t}\|$ as follows:

$$\|\tilde{H}_{\rm t}\| \le \tau(q+2) + 2g_0(q+1) \le (q+2)(\tau + 2g_0). \tag{170}$$

which is roughly as large as $\mathcal{O}(q\tau)$.

If the cut-off energy τ becomes sufficiently large, we expect that the low-energy behavior of both Hamiltonians $H_{\rm t}$ and $\tilde{H}_{\rm t}$ are approximately identical. We now want to know the τ-dependence of the accuracy of the low-energy spectrum of $\tilde{H}$ compared to that of the original Hamiltonian. The accuracy has been investigated by Arad, Kuwahara and Landau [7] when the energy cut-off is considered only for a single block Hamiltonian. Unfortunately, the accuracy of the multi-energy cut-off has not been considered so far, and the generalization to the multi-energy cut-off necessitates highly intricate analyses (see Supplementary Note 4).

We prove the following theorem, which ensures the exponentially accurate approximation with respect to the value of $\tau \gg \log(q)$:

Theorem 5. *Let us choose* τ *such that*

$$\tau \ge \max\left[8g_0 + \frac{1}{\lambda'}\log\left(\frac{88g_0(q+1)(q+2)}{\Delta_{\rm t}}\right), 4g_0 + \frac{1}{\lambda}\log\left(\frac{432(q+2)}{\lambda\Delta_{\rm t}}\right)\right], \tag{171}$$

where g_0 *has been defined in* (15) *and* $\{\lambda, \lambda'\}$ *are defined as follows:*

$$\lambda := \frac{1}{12k^2 + 4g_0}, \quad \lambda' := \min\left(\frac{1}{112g_0}, \frac{1}{12k^2}\right). \tag{172}$$

Then, the spectral gap $\tilde{\Delta}_{\rm t}$ *of the effective Hamiltonian is preserved as*

$$\tilde{\Delta}_{\rm t} \ge \frac{1}{2}\Delta_{\rm t}. \tag{173}$$

Moreover, the norm distance between the original ground state $|0_{\rm t}\rangle$ *and the effective one* $|\tilde{0}_{\rm t}\rangle$ *is exponentially small with respect to the cut-off energy* τ*:*

$$\||\tilde{0}_{\rm t}\rangle - |0_{\rm t}\rangle\| \le \frac{54(q+2)}{\lambda\Delta_{\rm t}} e^{-\lambda(\tau - 4g_0)}. \tag{174}$$

We show the proof of this theorem in Supplementary Note 4.

1. *Proof of Lemma 12*

For the proof, we aim to find energy shifts $\{\mathcal{E}_s\}_{s=0}^{q+1}$ with $\sum_{s=0}^{q+1} \mathcal{E}_s = 0$ such that the inequality (169) is satisfied. For the purpose, we first consider a quantum state $|\psi\rangle := \bigotimes_{s=0}^{q+1} |E_{0,s}\rangle$. We then obtain

$$\langle\psi|H_{\rm t}|\psi\rangle \ge E_{{\rm t},0} = 0 \tag{175}$$

and

$$\langle\psi|\left(\sum_{s=0}^{q+1} h_s + \sum_{s=0}^{q} h_{s,s+1}\right)|\psi\rangle = \sum_{s=0}^{q+1} E_{s,0} + \sum_{s=0}^{q} \langle\psi|h_{s,s+1}|\psi\rangle \le \sum_{s=0}^{q+1} E_{s,0} + (q+1)g_0, \tag{176}$$

where we use $\|h_{s,s+1}\| \le g_0$ as shown in Ineq. (46). Also, we have

$$0 = E_{{\rm t},0} \ge \sum_{s=0}^{q+1} E_{s,0} - \sum_{s=0}^{q} \|h_{s,s+1}\| \ge \sum_{s=0}^{q+1} E_{s,0} - (q+1)g_0. \tag{177}$$

By combining the above three inequalities, we have

$$-(q+1)g_0 \leq \sum_{s=0}^{q+1} E_{s,0} \leq (q+1)g_0. \tag{178}$$

We here shift the energy origins such that $E_{s,0} + \mathcal{E}_s = E_{s',0} + \mathcal{E}_{s'}$ for $\forall s, s'$, which implies

$$(q+2)(E_{s,0} + \mathcal{E}_s) = \sum_{s=0}^{q+1}(E_{s,0} + \mathcal{E}_s) = \sum_{s=0}^{q+1} E_{s,0},$$

$$\text{or} \quad \mathcal{E}_s = -E_{s,0} + \frac{1}{q+2}\sum_{s=0}^{q+1} E_{s,0} \tag{179}$$

for arbitrary $\{\mathcal{E}_s\}_{s=0}^{q+1}$, where we use the condition $\sum_{s=0}^{q+1} \mathcal{E}_s = 0$. Conversely, the above choice satisfies $\sum_{s=0}^{q+1} \mathcal{E}_s = 0$. From the inequality (178), the above choice of $\mathcal{E}_s$ leads to

$$-\frac{q+1}{q+2}g_0 \leq E_{s,0} + \mathcal{E}_s \leq \frac{q+1}{q+2}g_0 \tag{180}$$

for $s = 0, 1, 2, \ldots, q+1$. This completes the proof. □

Supplementary Note 3. PROOF OF MAIN THEOREM 1

We now have all the ingredients to prove the main theorem. Proof of Theorem 1 consists of the following two Propositions which we will prove in the subsequent subsections. In the first proposition, we prove the existence of a quantum state which has an $\mathcal{O}(1)$ overlap with the exact ground state and has a small Schmidt rank.

Proposition 6. *There exists a quantum state* $|\phi\rangle$ *such that*

$$\||0\rangle - |\phi\rangle\| \leq \frac{1}{2} \tag{181}$$

with

$$\log\left[\mathrm{SR}(|\phi\rangle)\right] \leq c^* \log^2(d)\left(\frac{\log(d)}{\Delta}\right)^{1+2/\bar{\alpha}} \log^{3+3/\bar{\alpha}}\left(\frac{\log(d)}{\Delta}\right), \tag{182}$$

where c^* *is a constant which depends only on* k*,* g_0*,* $\bar{\alpha}$*, which is finite in the limit of* $\bar{\alpha} \to \infty$*.*

In the second proposition, by using the quantum state given in Proposition 6, we construct an approximate ground state with a desired accuracy and estimate the Schmidt rank of the state. Based on this approximation, we also give the upper bound of the entanglement entropy.

Proposition 7. *Let* $|\phi\rangle$ *be an arbitrary quantum state such that*

$$\||0\rangle - |\phi\rangle\| \leq \frac{1}{2} \tag{183}$$

with $D_\phi := \mathrm{SR}(|\phi\rangle)$*. Then, there exists a quantum state which approximates the ground state* $|0\rangle$ *by*

$$\||0\rangle - |\psi\rangle\| \leq \delta \tag{184}$$

with the state $|\psi\rangle$ *satisfying*

$$\log[\mathrm{SR}(|\psi\rangle)]\| \leq \log(D_\phi) + c_1\bar{\alpha}^{-1}\frac{\log^{5/2}[2/(\delta\Delta)]}{\sqrt{\Delta}} + c_2\frac{\log^{3/2}[2/(\delta\Delta)]\log(d)}{\sqrt{\Delta}}. \tag{185}$$

Also, the entanglement entropy $S(|0\rangle)$ *is bounded from above by*

$$S(|0\rangle) \leq \log(D_\phi) + c_3\bar{\alpha}^{-1}\frac{\log^{5/2}(3/\Delta)}{\sqrt{\Delta}} + c_4\frac{\log^{3/2}(3/\Delta)\log(d)}{\sqrt{\Delta}}. \tag{186}$$

Here, c_1, c_2, c_3, c_4 *are constants of* $\mathcal{O}(1)$ *which depend only on* k*,* g_0*.*

By applying Proposition 6 to Proposition 7, we immediately prove Theorem 1. □

A. Proof of Proposition 6

In the following, we choose the number of the blocks q such that

$$\|\delta H_{\rm t}\| \le \frac{\Delta}{8} \quad \text{or} \quad \Delta_{\rm t} \ge \frac{3}{4}\Delta, \tag{187}$$

where the second inequality is derived from (58), namely $\Delta_{\rm t} \ge \Delta - 2\|\delta H_{\rm t}\|$. The inequality (187) implies from Ineq. (53)

$$\|\delta H_{\rm t}\| \le g_0 q l^{-\bar{\alpha}} \le \frac{\Delta}{8}, \\ \text{or} \quad q = \left\lfloor \frac{\Delta}{8g_0} l^{\bar{\alpha}} \right\rfloor = \mathcal{O}(l^{\bar{\alpha}}\Delta). \tag{188}$$

From Ineq. (60) in Lemma 4, the above choice of q gives the following inequality:

$$\||0\rangle - |\phi\rangle\| \le \||0_{\rm t}\rangle - |\phi\rangle\| + \frac{1}{4} \tag{189}$$

for an arbitrary quantum state $|\phi\rangle$. From Proposition 2, if we find an AGSP operator $K_{\rm t}$ such that

$$\epsilon_{K_{\rm t}}^2 D_{K_{\rm t}} \le \frac{1}{2}, \tag{190}$$

there exists a quantum state $|\psi\rangle$ which satisfies

$$\||0_{\rm t}\rangle - |\psi\rangle\| \le \epsilon_{K_{\rm t}}\sqrt{2D_{K_{\rm t}}} + \delta_{K_{\rm t}} \quad \text{with} \quad \mathrm{SR}(|\psi\rangle) \le D_{K_{\rm t}}. \tag{191}$$

Hence, if we can prove the existence of the AGSP operator which satisfies

$$\epsilon_{K_{\rm t}}\sqrt{2D_{K_{\rm t}}} + \delta_{K_{\rm t}} \le \frac{1}{4}, \tag{192}$$

$$\log(D_{K_{\rm t}}) \le c^* \log^2(d) \left(\frac{\log(d)}{\Delta}\right)^{1+2/\bar{\alpha}} \log^{3+3/\bar{\alpha}}\left(\frac{\log(d)}{\Delta}\right), \tag{193}$$

we prove the Proposition 6 by replacing $|\phi\rangle$ with $|\psi\rangle$ in Ineq. (189).

In the construction of the AGSP operator, we utilize the effective Hamiltonian from the truncated Hamiltonian $H_{\rm t}$. We here consider the operator $K(m, \tilde{H}_{\rm t})$ as the AGSP operator $K_{\rm t}$ for the ground state $|0_{\rm t}\rangle$, where the function $K(m,x)$ was defined in Eq. (162). It has the parameters $(\delta_{K_{\rm t}}, \epsilon_{K_{\rm t}}, D_{K_{\rm t}})$ as defined in (42), which depend only on m, q and τ. We need to appropriately determine these three parameters so that the conditions (190), (192) and (193) are satisfied.

The parameters are bounded from above as follows. First, from Ineq (174) in Theorem 5, we obtain

$$\delta_{K_{\rm t}} \le \frac{54(q+2)}{\lambda\Delta_{\rm t}} e^{-\lambda(\tau-4g_0)} \le \frac{216(q+2)}{3\lambda\Delta} e^{-\lambda(\tau-4g_0)} \tag{194}$$

under the condition of (171), where we use $\Delta_{\rm t} \ge \frac{3}{4}\Delta$ as in (187). Second, from Ineqs. (164) and (170), we obtain

$$\epsilon_{K_{\rm t}} \le 2e^{-2m\sqrt{\tilde{\Delta}_{\rm t}/\|\tilde{H}_{\rm t}\|}} \le 2\exp\left(-2m\sqrt{\frac{\tilde{\Delta}_{\rm t}}{(q+2)(\tau+2g_0)}}\right) \le 2\exp\left(-2m\sqrt{\frac{3\Delta}{8(q+2)(\tau+2g_0)}}\right), \tag{195}$$

where we use $\tilde{\Delta}_{\rm t} \ge \Delta_{\rm t}/2 \ge \frac{3}{8}\Delta$ in the third inequality. Third, from Proposition 4, we obtain

$$D_{K_{\rm t}} = \mathrm{SR}[K(m, \tilde{H}_{\rm t})] \le md^{2ql}[e(q+1)^2(2dl)^k]^{m/(q+1)} \tag{196}$$

under the assumption of $(q+m+1)^{q+1} \le d^{ql}$.

We choose τ such that $\delta_{K_{\rm t}} \le 1/8$, or equivalently from Ineq. (194),

$$\tau \ge 4g_0 + \frac{1}{\lambda}\log\left(\frac{1728}{3\lambda}\frac{q+2}{\Delta}\right) \quad \to \quad \tau = c_\tau \log(q/\Delta), \tag{197}$$

where c_τ is a constant depending only on g_0 and k which is chosen to satisfy the condition of (171). Then, for the inequality (192) to be satisfied, we need to choose m and q such that

$$\epsilon_{K_{\rm t}}\sqrt{2D_{K_{\rm t}}} \le \frac{1}{8}. \tag{198}$$

From the inequalities (195) and (196), we can formally give ϵ_{K_t} and D_{K_t} as

$$\epsilon_{K_t} \le \exp\left(-b_1 m \sqrt{\frac{\Delta}{q\log(q/\Delta)}}\right), \quad D_{K_t} \le \exp\left(c_1 \log(d) q (q/\Delta)^{1/\bar{\alpha}} + c_2 \frac{\log(dq/\Delta) m}{q}\right), \tag{199}$$

where the constants b_1, c_1 depend only on $\{g_0, k\}$, and c_2 depends on $\{\bar{\alpha}, g_0, k\}$. Also, the constant c_2 is proportional to $1/\bar{\alpha}$ and hence it is finite in the limit of $\bar{\alpha} \to \infty$. Note that in Eq. (199) we utilize $l \propto (q/\Delta)^{1/\bar{\alpha}}$ from Eq. (188).

In the following, we choose m and q as such $\epsilon_{K_t} D_{K_t} \le \epsilon_K^{1/2}$, which reduces the conditions (190) and (198) to

$$\epsilon_{K_t}^2 D_{K_t} \le \epsilon_{K_t}^{3/2} \le \frac{1}{2}, \qquad \epsilon_{K_t}\sqrt{2D_{K_t}} \le \sqrt{2}\epsilon_{K_t}^{3/4} \le \frac{1}{8}. \tag{200}$$

From the inequalities in (199), the condition $\epsilon_{K_t} D_{K_t} \le \epsilon_{K_t}^{1/2}$ is satisfied for

$$\begin{aligned} c_1 \log(d) q (q/\Delta)^{1/\bar{\alpha}} &\le \frac{b_1}{4} m \sqrt{\frac{\Delta}{q\log(q/\Delta)}}, \\ c_2 \frac{\log(dq/\Delta)m}{q} &\le \frac{b_1}{4} m \sqrt{\frac{\Delta}{q\log(q/\Delta)}}. \end{aligned} \tag{201}$$

The first inequality in (201) gives the lower bound of m as follows:

$$m \ge \frac{4c_1\log(d)}{b_1} \Delta^{-1/2-1/\bar{\alpha}} q^{3/2+1/\bar{\alpha}} \sqrt{\log(q/\Delta)}. \tag{202}$$

The second one implies

$$\frac{1}{\log(q/\Delta)}\sqrt{\frac{q}{\log(q/\Delta)}} \ge \frac{4c_2}{b_1}\frac{\log(d)}{\sqrt{\Delta}}, \tag{203}$$

which is satisfied for

$$q \ge \frac{c_q c_2 \log^2(d)}{\Delta} \log^3\left(\frac{\log(d)}{\Delta}\right), \tag{204}$$

where c_q is a constant depending only on k and g_0. By choosing $q = \left\lceil \frac{c_q c_2 \log^2(d)}{\Delta} \log^3\left(\frac{\log(d)}{\Delta}\right)\right\rceil$, the parameters ϵ_K exponentially decays with m. Hence, there exists a constant c_m such that

$$m = c_m c_2 \log(d) \Delta^{-1/2-1/\bar{\alpha}} \left(\frac{\log^2(d)}{\Delta}\right)^{3/2+1/\bar{\alpha}} \log^{5+3/\bar{\alpha}}\left(\frac{\log(d)}{\Delta}\right) \tag{205}$$

satisfies (200) and (202), where c_m is a constant depending only on k and g_0.

Finally, under the above choices of m and l, the Schmidt rank D_{K_t} is bounded from above as

$$\log(D_{K_t}) \le c^* \log^2(d) \left(\frac{\log(d)}{\Delta}\right)^{1+2/\bar{\alpha}} \log^{3+3/\bar{\alpha}}\left(\frac{\log(d)}{\Delta}\right), \tag{206}$$

where c^* is a constant depending only on k, g_0, $\bar{\alpha}$ and c^* is finite in the limit of $\bar{\alpha} \to \infty$. We thus obtain Eq. (193). This completes the proof. □

B. Proof of Proposition 7

In the proof, we utilize Proposition 3. For the purpose, we set $q = 2$ and construct the AGSP operator for $|0\rangle$ by using $K(m, \tilde{H}_t)$. From Ineq. (53) with $q = 2$, we obtain the upper bound of $\|\delta H_t\|$ as

$$\|\delta H_t\| \le 2g_0 l^{-\bar{\alpha}}. \tag{207}$$

From the inequality (58), namely $\Delta_t \ge \Delta - 2\|\delta H_t\|$, the condition

$$\|\delta H_t\| \le 2g_0 l^{-\bar{\alpha}} \le \frac{\Delta}{8} \tag{208}$$

implies $\Delta_{\rm t} \geq 3\Delta/4$. In the following discussions, we choose l as

$$l \geq \left(\frac{16g_0}{\Delta}\right)^{1/\bar{\alpha}} \tag{209}$$

so that the condition (208) is satisfied. Also, from Lemma 4, for an arbitrary quantum state $|\phi\rangle$, we have

$$\begin{aligned} \||0\rangle - |\phi\rangle\| &\leq \||0_{\rm t}\rangle - |\phi\rangle\| + \frac{\|\delta H_{\rm t}\|}{\Delta - 4\|\delta H_{\rm t}\|} \\ &\leq \||0_{\rm t}\rangle - |\phi\rangle\| + \frac{2\|\delta H_{\rm t}\|}{\Delta} \leq \||0_{\rm t}\rangle - |\phi\rangle\| + \frac{4g_0 l^{-\bar{\alpha}}}{\Delta}, \end{aligned} \tag{210}$$

where we use Ineq. (208) in the first inequality and Ineq. (207) in the second inequality.

For the construction of the AGSP operator, we utilize the effective Hamiltonian from the truncated Hamiltonian $H_{\rm t}$ with $q = 2$. We here assume for τ the condition (171), which is now given by

$$\tau \geq \max\left[8g_0 + \frac{1}{\lambda'}\log\left(\frac{1408g_0}{\Delta}\right), 4g_0 + \frac{1}{\lambda}\log\left(\frac{2304}{\lambda\Delta}\right)\right]. \tag{211}$$

Then, Theorem 5 gives

$$\||\tilde{0}_{\rm t}\rangle - |0_{\rm t}\rangle\| \leq \frac{216}{\lambda\Delta_{\rm t}} e^{-\lambda(\tau - 4g_0)} \leq \frac{288}{\lambda\Delta} e^{-\lambda(\tau-4g_0)}, \tag{212}$$

$$\tilde{\Delta}_{\rm t} \geq \frac{\Delta_{\rm t}}{2} \geq \frac{3\Delta}{8}, \tag{213}$$

where we use $\Delta_t \geq 3\Delta/4$ in the second inequality.

Here, the operator $K(m, \tilde{H}_{\rm t})$ depends only on the parameters m, l and τ, and hence we denote it by $K_{m,l,\tau}$ and define the AGSP parameters as $(\delta_{m,l,\tau}, \epsilon_{m,l,\tau}, D_{m,l,\tau})$. We note that the parameters $(\delta_{m,l,\tau}, \epsilon_{m,l,\tau}, D_{m,l,\tau})$ do not depend on the choice of the energy origin for $H_{\rm t}$. Hence, when we consider the truncated Hamiltonians $H_{\rm t}$ with different parameters q and l, we will retake the energy origin so that $E_{{\rm t},0} = 0$.

The upper bounds of the parameters are given as follows. First, $\delta_{m,l,\tau}$ is bounded from above by

$$\delta_{m,l,\tau} = \||0\rangle - |\tilde{0}_{\rm t}\rangle\| \leq \frac{288}{\lambda\Delta} e^{-\lambda(\tau-4g_0)} + \frac{4g_0 l^{-\bar{\alpha}}}{\Delta}, \tag{214}$$

where we use the inequality (210) with $|\phi\rangle = |\tilde{0}_{\rm t}\rangle$ and apply Ineq. (212) to $\||0_{\rm t}\rangle - |\tilde{0}_{\rm t}\rangle\|$. Second, we obtain a similar inequality to (195) as

$$\epsilon_{m,l,\tau} \leq 2\exp\left(-2m\sqrt{\frac{\tilde{\Delta}_{\rm t}}{(q+2)(\tau+2g_0)}}\right) \leq 2\exp\left(-m\sqrt{\frac{3\Delta}{8(\tau+2g_0)}}\right), \tag{215}$$

where we use $q = 2$ and $\tilde{\Delta}_{\rm t} \geq 3\Delta/8$ in the inequality (213). Third, from Lemma 8, we obtain

$$D_{m,l,\tau} \leq [2 + (2dl)^k]^m = e^{m\mathcal{O}(\log(ld))}. \tag{216}$$

In the following, we consider a sequence of the AGSP operator $\{K_p\}_{p=1}^{\infty}$. We here characterize the AGSP parameters of K_p by $\delta_p = \delta_{m_p,l_p,\tau_p}$, $\epsilon_p = \epsilon_{m_p,l_p,\tau_p}$ and $D_p = D_{m_p,l_p,\tau_p}$. From Proposition 3, the entanglement entropy $S(|0\rangle)$ is bounded from above by

$$S(|0\rangle) \leq \log(3D_\phi D_1) - \sum_{p=1}^{\infty} \gamma_p^2 \log\frac{\gamma_p^2}{3D_{p+1}} \quad \text{with} \quad \gamma_p := \frac{\epsilon_p}{1 - \nu_0 - \delta_p} + \delta_p, \tag{217}$$

where $\nu_0 := \||0\rangle - |\phi\rangle\|$ and $D_\phi = {\rm SR}(|\phi\rangle)$. Note that the quantum state ϕ is given as in Eq. (183) and satisfies $\nu_0 \leq 1/2$. Moreover, the quantity γ_p characterizes the norm distance between $|0\rangle$ and $K_p|\phi\rangle$ (see Ineq. (111)). We then choose $\{m_p, l_p, \tau_p\}$ such that

$$\gamma_p \leq \frac{1}{p}, \tag{218}$$

which is satisfied by choosing

$$\delta_p \leq \frac{1}{3p}, \quad \frac{\epsilon_p}{1-\nu_0-\delta_p} \leq \frac{2}{3p} \quad \text{or} \quad \epsilon_p \leq \frac{1}{9p}, \tag{219}$$

where the second inequality is derived from

$$\frac{\epsilon_p}{1-\nu_0-\delta_p} \le \frac{\epsilon_p}{1/2-\delta_p} \le \frac{\epsilon_p}{1/2-1/3} = 6\epsilon_p \le \frac{2}{3p}. \tag{220}$$

In the first inequality, we use $\nu_0 \le 1/2$.

From the inequality (214), the following choices of τ_p and l_p ensure the condition $\delta_p \le 1/(3p)$:

$$\frac{288}{\lambda\Delta} e^{-\lambda(\tau_p - 4g_0)} \le \frac{1}{6p}, \quad \frac{4g_0 l_p^{-\bar{\alpha}}}{\Delta} \le \frac{1}{6p}, \tag{221}$$

which implies

$$\tau_p \ge 4g_0 + \frac{\lambda_p}{\lambda} \log\left(\frac{1728p}{\lambda\Delta}\right), \quad l_p \ge \left(\frac{24g_0 p}{\Delta}\right)^{1/\bar{\alpha}}, \tag{222}$$

where λ_p is a constant for the condition (211) to be satisfied. Note that λ_p depends only on k and g_0. For $\epsilon_p \le 1/(9p)$, we choose m_p such that

$$2\exp\left(-m_p\sqrt{\frac{3\Delta}{8(\tau_p+2g_0)}}\right) \le \frac{1}{9p}, \tag{223}$$

which yields from Ineq. (222)

$$m_p \ge \frac{\log(18p)}{\sqrt{\Delta}} \sqrt{\frac{48g_0}{3} + \frac{8\lambda_p}{3\lambda}\log\left(\frac{1728p}{\lambda\Delta}\right)}. \tag{224}$$

Under the above choice, the Schmidt rank (216) is formally given by

$$\log(3D_p) \le c_1\bar{\alpha}^{-1}\frac{\log^{5/2}(3p/\Delta)}{\sqrt{\Delta}} + c_2\frac{\log^{3/2}(3p/\Delta)\log(d)}{\sqrt{\Delta}} \tag{225}$$

with c_1 and c_2 constants which depend only on k, g_0. We note that the dependence of $\bar{\alpha}$ results from (222), and hence $\log(3D_p)$ are finitely bounded in the limit of $\bar{\alpha} \to \infty$. Thus, by using Ineq. (111) with Eq. (112), we obtain the inequality (185) in Proposition 7.

Finally, from the inequality (217), we have

$$\begin{aligned} S(|0\rangle) &\le \log(3D_\phi D_1) + \sum_{p=1}^{\infty}\frac{1}{p^2}\left(\log(p^2) + c_1\bar{\alpha}^{-1}\frac{\log^{5/2}[3(p+1)/\Delta]}{\sqrt{\Delta}} + c_2\frac{\log^{3/2}[3(p+1)/\Delta]\log(d)}{\sqrt{\Delta}}\right) \\ &\le \log(D_\phi) + c_3\bar{\alpha}^{-1}\frac{\log^{5/2}(3/\Delta)}{\sqrt{\Delta}} + c_4\frac{\log^{3/2}(3/\Delta)\log(d)}{\sqrt{\Delta}}, \end{aligned} \tag{226}$$

where c_3 and c_4 are constants which depend only on k, g_0. We also use $\sum_{p=1}^{\infty} p^{-2}\log(p^2) = 1.875095\cdots$. We thus obtain the upper bound (186) for the entanglement entropy. This completes the proof of Proposition 7. □

Supplementary Note 4. PROOF OF THEOREM 5: ACCURACY OF THE EFFECTIVE HAMILTONIAN WITH MULTI-ENERGY CUT-OFF

In this section, we show the proof of Theorem 5. Throughout the section, we explicitly take the parameter g into account; in the setup, g was defined in (5) and set to be 1. For the convenience for the reader, we show the statement again in the following:

Theorem 5. *Let us choose τ such that*

$$\tau \ge \max\left[8g_0 + \frac{1}{\lambda'}\log\left(\frac{88g_0(q+1)(q+2)}{\Delta_{\rm t}}\right), 4g_0 + \frac{1}{\lambda}\log\left(\frac{432(q+2)}{\lambda\Delta_{\rm t}}\right)\right], \tag{227}$$

where g_0 has been defined in (15) and $\{\lambda, \lambda'\}$ are defined as follows:

$$\lambda := \frac{1}{12gk^2 + 4g_0}, \quad \lambda' := \min\left(\frac{1}{112g_0}, \frac{1}{12gk^2}\right). \tag{228}$$

Then, the spectral gap $\tilde{\Delta}_{\rm t}$ of the effective Hamiltonian is preserved as

$$\tilde{\Delta}_{\rm t} \ge \frac{1}{2}\Delta_{\rm t}. \tag{229}$$

Moreover, the norm distance between the original ground state $|0_{\rm t}\rangle$ and the effective one $|\tilde{0}_{\rm t}\rangle$ is exponentially small with respect to the cut-off energy τ:

$$\||\tilde{0}_{\rm t}\rangle - |0_{\rm t}\rangle\| \le \frac{54(q+2)}{\lambda\Delta_{\rm t}} e^{-\lambda(\tau-4g_0)}. \tag{230}$$

We notice that in Supplementary Note 2 I the above theorem was given by setting $g=1$ in the definitions of λ and λ'.

A. Preliminaries

We first define the projection operator onto the eigenspace of h_s as

$$\Pi_I^{(s)} = \sum_{E_{s,j}\in I} |E_{s,j}\rangle\langle E_{s,j}| \tag{231}$$

for $I \subset \mathbb{R}$. Especially for $\Pi^{(s)}_{(-\infty,x)}$ and $\Pi^{(s)}_{(-\infty,x]}$, we denote them by $\Pi^{(s)}_{<x}$ and $\Pi^{(s)}_{\le x}$, respectively. In the same way, we define $\Pi^{(s)}_{>x}$ and $\Pi^{(s)}_{\ge x}$. By using the above notations, we describe the effective Hamiltonian $\tilde{H}_{\rm t}$ in Eq. (50) as

$$\tilde{H}_{\rm t} = \sum_{s=0}^{q+1} \tilde{h}_s + \sum_{s=0}^{q} h_{s,s+1}, \quad \tilde{h}_s := h_s \Pi^{(s)}_{\le \tau_s} + \tau_s \Pi^{(s)}_{>\tau_s} \tag{232}$$

for $s = 0,1,2\ldots,q+1$, where we choose the cut-off energies $\{\tau_s\}_{s=0}^{q+1}$ as

$$\tau_s = E_{s,0} + \tau \quad \text{for} \quad s = 0,1,2\ldots,q+1 \tag{233}$$

For the total Hamiltonian $H_{\rm t}$ and the effective Hamiltonian $\tilde{H}_{\rm t}$, we define Π_I and $\tilde{\Pi}_I$ as

$$\Pi_I = \sum_{E_{{\rm t},j}\in I} |E_{{\rm t},j}\rangle\langle E_{{\rm t},j}|,$$
$$\tilde{\Pi}_I = \sum_{\tilde{E}_{{\rm t},j}\in I} |\tilde{E}_{{\rm t},j}\rangle\langle \tilde{E}_{{\rm t},j}|. \tag{234}$$

We here define $\{E_{{\rm t},j}, |E_{{\rm t},j}\rangle\}_j$ are the eigenvalues and the eigenstates of $H_{\rm t}$, respectively. Also, we define $\{\tilde{E}_{{\rm t},j}, |\tilde{E}_{{\rm t},j}\rangle\}_j$ are the eigenvalues and the eigenstates of $\tilde{H}_{\rm t}$, respectively.

For $\Pi_{(-\infty,x)}$ (or $\Pi_{(-\infty,x]}$) and $\tilde{\Pi}_{(-\infty,x)}$ (or $\tilde{\Pi}_{(-\infty,x]}$), we denote them by $\Pi_{<x}$ (or $\Pi_{\le x}$) and $\tilde{\Pi}_{<x}$ (or $\tilde{\Pi}_{\le x}$), respectively.

1. *Upper bound of the spectral gap for $\tilde{H}_{\rm t}$*

We first estimate the upper bound of the spectral gap $\tilde{\Delta}_{\rm t}$ for the effective Hamiltonian $\tilde{H}_{\rm t}$, which is necessary in utilizing the inequality (280) in Proposition 8. We can prove the following lemma.

Lemma 13. *The spectral gap $\tilde{\Delta}_{\rm t} = \tilde{E}_{{\rm t},1} - \tilde{E}_{{\rm t},0}$ is bounded from above by*

$$\tilde{\Delta}_{\rm t} \le 4g_0, \tag{235}$$

where g_0 has been defined in (15).

Proof of Lemma 13. For the proof, let us pick up the block B_0 and consider a quantum state

$$|\psi\rangle = (a_0|E_{0,0}\rangle + a_1|E_{0,1}\rangle) \otimes |\tilde{E}_{\Lambda_0,0}\rangle, \tag{236}$$

where $|\tilde{E}_{\Lambda_0,0}\rangle$ is the minimum energy state for $\tilde{H}_{\rm t} - \tilde{h}_{s=0} - h_{s=0,s=1}$ and is supported on $\Lambda_0 := \Lambda \setminus B_0$. Note that $|E_{0,0}\rangle$ and $|E_{0,1}\rangle$ have been defined as the ground state and the first excited state of $h_{s=0}$, respectively. We define the energy $\langle \tilde{E}_{\Lambda_0,0}|(\tilde{H}_{\rm t} - \tilde{h}_{s=0} - h_{s=0,s=1})|\tilde{E}_{\Lambda_0,0}\rangle$ as $\tilde{E}_{\Lambda_0,0}$. Here, we choose a_0 and a_1 such that $\langle\psi|\tilde{0}_{\rm t}\rangle = 0$. We then obtain

$$\begin{aligned}\langle\psi|\tilde{H}_{\rm t}|\psi\rangle &= (|a_0|^2 E_{0,0} + |a_1|^2 E_{0,1}) + \tilde{E}_{\Lambda_0,0} + \langle\psi|h_{s=0,s=1}|\psi\rangle \\ &\le E_{0,1} + \tilde{E}_{\Lambda_0,0} + \|h_{s=0,s=1}\| \le E_{0,0} + \tilde{E}_{\Lambda_0,0} + 2g + g_0,\end{aligned} \tag{237}$$

where we use $\|h_{s,s+1}\| \le g_0$ for $\forall s \in \{0,1,\dots,q+1\}$ and $E_{0,1} - E_{0,0} \le 2g$ from the inequality (7).

On the other hand, we have for the ground state $|\tilde{0}_{\rm t}\rangle$

$$\langle \tilde{0}_{\rm t}|\tilde{H}_{\rm t}|\tilde{0}_{\rm t}\rangle \ge E_{0,0} + \tilde{E}_{\Lambda_0,0} - \|h_{s=0,s=1}\| \ge E_{0,0} + \tilde{E}_{\Lambda_0,0} - g_0. \tag{238}$$

We thus obtain the upper bound of $\tilde{\Delta}_{\rm t}$ as follows

$$\tilde{\Delta}_{\rm t} \le \langle \psi|\tilde{H}_{\rm t}|\psi\rangle - \langle \tilde{0}_{\rm t}|\tilde{H}_{\rm t}|\tilde{0}_{\rm t}\rangle \le 2g + 2g_0 \le 4g_0, \tag{239}$$

where we use $g_0 \ge 1 = g$ (see Assumption 1). This completes the proof of Lemma 13. □

2. *Lower bound of the spectral gap $\tilde{\Delta}_{\rm t}$ for $\tilde{H}_{\rm t}$*

In the following lemma, we obtain a lower bound of $\tilde{\Delta}_{\rm t}$:

Lemma 14. *Let us define the spectral decomposition of $|0_{\rm t}\rangle$ as follows:*

$$|0_{\rm t}\rangle = a_0|\tilde{0}_{\rm t}\rangle + a_1|\tilde{E}_{{\rm t},1}\rangle + \sum_{j\ge 2} a_j|\tilde{E}_{{\rm t},j}\rangle. \tag{240}$$

Then, the quantum state

$$|\psi\rangle = \frac{1}{\sqrt{|a_0|^2 + |a_1|^2}} \left(a_1^*|\tilde{0}_{\rm t}\rangle - a_0^*|\tilde{E}_{{\rm t},1}\rangle\right) \tag{241}$$

gives

$$|\psi\rangle = \arg \inf_{\psi:\langle\psi|0_{\rm t}\rangle=0} (\langle\psi|\tilde{H}_{\rm t}|\psi\rangle). \tag{242}$$

Also, the spectral gap of the Hamiltonian $\tilde{H}_{\rm t}$ is bounded from below by

$$\tilde{\Delta}_{\rm t} \ge \langle\psi|\tilde{H}_{\rm t}|\psi\rangle - \langle 0_{\rm t}|\tilde{H}_{\rm t}|0_{\rm t}\rangle. \tag{243}$$

Proof of Lemma 14. First, the quantum state as $\arg\inf_{\psi:\langle\psi|0_{\rm t}\rangle=0}(\langle\psi|\tilde{H}_{\rm t}|\psi\rangle)$ is given in the form of

$$|\psi\rangle = b_0|\tilde{0}_{\rm t}\rangle + b_1|\tilde{E}_{{\rm t},1}\rangle. \tag{244}$$

Then, due to the constraint of $\langle\psi|0_{\rm t}\rangle = 0$, the coefficients $\{b_0, b_1\}$ are uniquely determined as

$$\{b_0, b_1\} = \frac{1}{\sqrt{|a_0|^2 + |a_1|^2}}\{a_1^*, -a_0^*\} \tag{245}$$

up to a phase factor. We thus prove Eq. (242).

Also, the definition (241) implies

$$\langle\psi|\tilde{H}_{\rm t}|\psi\rangle = \frac{|a_1|^2\tilde{E}_{{\rm t},0} + |a_0|^2\tilde{E}_{{\rm t},1}}{|a_0|^2 + |a_1|^2}. \tag{246}$$

On the other hand, the decomposition (240) yields

$$\langle 0_{\rm t}|\tilde{H}_{\rm t}|0_{\rm t}\rangle \ge \frac{|a_0|^2\tilde{E}_{{\rm t},0} + |a_1|^2\tilde{E}_{{\rm t},1}}{|a_0|^2 + |a_1|^2}. \tag{247}$$

By combining the inequalities (246) and (247), we obtain

$$\langle\psi|\tilde{H}_{\rm t}|\psi\rangle - \langle 0_{\rm t}|\tilde{H}_{\rm t}|0_{\rm t}\rangle \le \frac{|a_0|^2 - |a_1|^2}{|a_0|^2 + |a_1|^2}(\tilde{E}_{{\rm t},1} - \tilde{E}_{{\rm t},0}) \le \tilde{\Delta}_{\rm t}. \tag{248}$$

This completes the proof of Lemma 14. □

3. Lower bound of $\langle\psi|\tilde{H}_{\rm t}|\psi\rangle$

In order to apply Lemma 14, we need to give a lower bound of $\langle\psi|\tilde{H}_{\rm t}|\psi\rangle$ with $|\psi\rangle$ defined in Eq. (241). It is given by the following lemma:

Lemma 15. *Let $|\psi\rangle$ be in the form of Eq. (241). We then obtain*

$$\langle\psi|\tilde{H}_{\rm t}|\psi\rangle \ge E_\perp, \tag{249}$$

where we define

$$E_\perp := \Delta_{\rm t}(1-\kappa)^2 - 2g_0\kappa(1+\kappa)(q+1), \tag{250}$$

$$\kappa := \sum_{s=0}^{q+1} \left\| \Pi^{(s)}_{>\tau_s} \tilde{\Pi}_{\le \tilde{E}_{\rm t,1}} \right\|. \tag{251}$$

We also notice that because of Eq. (242) we have

$$\langle\phi|\tilde{H}_{\rm t}|\phi\rangle \ge \langle\psi|\tilde{H}_{\rm t}|\psi\rangle \ge E_\perp \tag{252}$$

for an arbitrary quantum state such that $\langle\phi|0_{\rm t}\rangle = 0$.

Proof of Lemma 15. Let P_s and Q_s be the projection operators such that

$$P_s := \Pi^{(s)}_{\le\tau_s}, \quad Q_s := \Pi^{(s)}_{>\tau_s} \tag{253}$$

for $s = 0, 1, \ldots, q+1$, where each of $\{P_s, Q_s\}_{s=0}^{q+1}$ is supported on the subset $B_s \subset \Lambda$. We define $P^{(m)}$ as follows:

$$P^{(m)} := P_0 P_1 \cdots P_m, \quad Q^{(m)} = 1 - P^{(m)}. \tag{254}$$

From Eq. (232), we can express $\tilde{h}_s$ as $\tilde{h}_s = h_s P_s + \tau_s Q_s$ (note that $\tilde{h}_s P_s = h_s P_s$), and hence

$$\tilde{H}_{\rm t} P^{(q+1)} = H_{\rm t} P^{(q+1)}. \tag{255}$$

We here prove the following inequality:

$$\|P^{(q+1)}|\psi\rangle - |\psi\rangle\| \le \sum_{s=0}^{q+1} \|Q_s|\psi\rangle\| \le \kappa \tag{256}$$

with κ defined in Eq. (251), where the second inequality is derived from

$$\|Q_s|\psi\rangle\| = \|\Pi^{(s)}_{>\tau_s}|\psi\rangle\| = \|\Pi^{(s)}_{>\tau_s}\tilde{\Pi}_{\le\tilde{E}_{\rm t,1}}|\psi\rangle\| \le \|\Pi^{(s)}_{>\tau_s}\tilde{\Pi}_{\le\tilde{E}_{\rm t,1}}\| \tag{257}$$

for $s \in \{0, 1, 2, \ldots, q+1\}$. Note that the definition (241) implies $|\psi\rangle = \tilde{\Pi}_{\le\tilde{E}_{\rm t,1}}|\psi\rangle$. In order to prove the first inequality in (256), we consider

$$P^{(q+1)}|\psi\rangle - |\psi\rangle = \sum_{s=0}^{q+1} [P^{(s)} - P^{(s-1)}]|\psi\rangle, \tag{258}$$

where we set $P^{(-1)} = 1$. Then, by combining the inequalities

$$\|P^{(q+1)}|\psi\rangle - |\psi\rangle\| \le \sum_{s=0}^{q+1} \|[P^{(s)} - P^{(s-1)}]|\psi\rangle\|, \tag{259}$$

and

$$\|[P^{(s)} - P^{(s-1)}]|\psi\rangle\| = \|P^{(s-1)}(P_s - 1)|\psi\rangle\| \le \|Q_s|\psi\rangle\|, \tag{260}$$

we obtain the first inequality in (256).

By using the notations of $P^{(q+1)}$ and $Q^{(q+1)}$, we obtain

$$\begin{aligned}\langle\psi|\tilde{H}_{\rm t}|\psi\rangle \ge& \langle\psi|P^{(q+1)}\tilde{H}_{\rm t}P^{(q+1)}|\psi\rangle - |\langle\psi|P^{(q+1)}\tilde{H}_{\rm t}Q^{(q+1)}|\psi\rangle| \\ &- |\langle\psi|Q^{(q+1)}\tilde{H}_{\rm t}P^{(q+1)}|\psi\rangle| + \langle\psi|Q^{(q+1)}\tilde{H}_{\rm t}Q^{(q+1)}|\psi\rangle.\end{aligned} \tag{261}$$

We consider the lower bound of the first term in (261). From Eq. (255) and $E_{\rm t,0}=0$, we obtain

$$\begin{aligned}\langle\psi|P^{(q+1)}\tilde{H}_{\rm t}P^{(q+1)}|\psi\rangle &= \langle\psi|P^{(q+1)}H_{\rm t}P^{(q+1)}|\psi\rangle \\ &= \langle\psi|P^{(q+1)}\Pi_{\geq\Delta_{\rm t}}H_{\rm t}\Pi_{\geq\Delta_{\rm t}}P^{(q+1)}|\psi\rangle \geq \Delta_{\rm t}\|\Pi_{\geq\Delta_{\rm t}}P^{(q+1)}|\psi\rangle\|^2 \end{aligned} \tag{262}$$

From the inequality (256), we immediately obtain

$$\begin{aligned}\|\Pi_{\geq\Delta_{\rm t}}P^{(q+1)}|\psi\rangle\| &\geq \|\Pi_{\geq\Delta_{\rm t}}|\psi\rangle\| - \left\|\Pi_{\geq\Delta_{\rm t}}\left(P^{(q+1)}|\psi\rangle - |\psi\rangle\right)\right\| \\ &\geq 1 - \|P^{(q+1)}|\psi\rangle - |\psi\rangle\| \geq 1-\kappa, \end{aligned} \tag{263}$$

where we use $\Pi_{\geq\Delta_{\rm t}}|\psi\rangle = |\psi\rangle$ due to $\langle 0_{\rm t}|\psi\rangle = 0$. By combining the above two inequalities, we obtain

$$\langle\psi|P^{(q+1)}\tilde{H}_{\rm t}P^{(q+1)}|\psi\rangle \geq \Delta_{\rm t}(1-\kappa)^2, \tag{264}$$

We then consider the second and third terms in (261). Because of $[P_s,\tilde{h}_s]=0$ and $P^{(q+1)}Q^{(q+1)}=0$, we have $P^{(q+1)}\tilde{h}_sQ^{(q+1)}=0$ for $s=0,1,2\ldots,q+1$. Hence, the second term is bounded from below as

$$\begin{aligned}-|\langle\psi|P^{(q+1)}\tilde{H}_{\rm t}Q^{(q+1)}|\psi\rangle| &\geq -\sum_{s=0}^{q}|\langle\psi|P^{(q+1)}h_{s,s+1}Q^{(q+1)}|\psi\rangle| \\ &\geq -g_0(q+1)\|(1-P^{(q+1)})|\psi\rangle\| \geq -g_0\kappa(q+1), \end{aligned} \tag{265}$$

where we use $\|h_{s,s+1}\|\leq g_0$ and $P^{(q+1)}+Q^{(q+1)}=1$ in the second inequality, and use Ineq. (256) in the last inequality. We obtain the same inequality for the third term. Finally, the fourth term is bounded from below by

$$\langle\psi|Q^{(q+1)}\tilde{H}_{\rm t}Q^{(q+1)}|\psi\rangle \geq \|Q^{(q+1)}|\psi\rangle\|^2\tilde{E}_{\rm t,0} \geq -2g_0\kappa^2(q+1), \tag{266}$$

where we use $\|Q^{(q+1)}|\psi\rangle\| = \|(1-P^{(q+1)})|\psi\rangle\| \leq \kappa$ from Ineq. (256) and

$$\tilde{E}_{\rm t,0} \geq \sum_{s=0}^{q+1}E_{s,0} - \sum_{s=0}^{q}\|h_{s,s+1}\| \geq -2(q+1)g_0. \tag{267}$$

Note that we use Ineq. (178) in order to bound $E_{s,0}$ from below. By applying the inequalities (264), (265) and (266) to (261), we prove the inequality (249). This completes the proof of Lemma 15. □

4. *Upper bound of the norm difference* $\||0_{\rm t}\rangle - |\tilde{0}_{\rm t}\rangle\|$

Finally, we prove the following lemma which estimate $\||0_{\rm t}\rangle - |\tilde{0}_{\rm t}\rangle\|$:

Lemma 16. *Under the assumption of*

$$\langle 0_{\rm t}|\tilde{H}_{\rm t}|0_{\rm t}\rangle < E_{\perp}, \tag{268}$$

the norm of $|0_{\rm t}\rangle - |\tilde{0}_{\rm t}\rangle$ *is bounded from above as follows:*

$$\||\tilde{0}_{\rm t}\rangle - |0_{\rm t}\rangle\| \leq \frac{\|\tilde{H}_{\rm t}|0_{\rm t}\rangle\|}{E_{\perp} - \langle 0_{\rm t}|\tilde{H}_{\rm t}|0_{\rm t}\rangle}. \tag{269}$$

Proof of Lemma 16. The proof is almost the same as that of Lemma 4. For the convenience for readers, we show the full proof. We first expand $|\tilde{0}_{\rm t}\rangle$ as follows:

$$|\tilde{0}_{\rm t}\rangle = \zeta_1|0_{\rm t}\rangle + \zeta_2|\phi_0\rangle, \tag{270}$$

where $\langle 0_{\rm t}|\phi_0\rangle = 0$ and we choose the phase term of $|\tilde{0}_{\rm t}\rangle$ so that $\langle 0_{\rm t}|\tilde{0}_{\rm t}\rangle$ has a positive real value, namely $|\zeta_1| = |\langle 0_{\rm t}|\tilde{0}_{\rm t}\rangle| = \langle 0_{\rm t}|\tilde{0}_{\rm t}\rangle = \zeta_1$. Then, the coefficients $\{\zeta_1,\zeta_2\}$ is determined by the eigen-problem of the following matrix:

$$\begin{pmatrix}\langle 0_{\rm t}|\tilde{H}_{\rm t}|0_{\rm t}\rangle & \langle 0_{\rm t}|\tilde{H}_{\rm t}|\phi_0\rangle \\ \langle\phi_0|\tilde{H}_{\rm t}|0_{\rm t}\rangle & \langle\phi_0|\tilde{H}_{\rm t}|\phi_0\rangle\end{pmatrix} =: \begin{pmatrix} f_0 & f \\ f^* & f_{\perp}\end{pmatrix}. \tag{271}$$

Then, the ground-state energy of $\tilde{H}_{\rm t}$ is formally given by

$$\tilde{E}_{\rm t,0} = \frac{f_0+f_{\perp}-\sqrt{(f_0-f_{\perp})^2+4|f|^2}}{2}, \tag{272}$$

and the corresponding coefficients $\{\zeta_1, \zeta_2\}$ are

$$\{\zeta_1, \zeta_2\} \propto \left\{ f_\perp - f_0 + \sqrt{(f_\perp - f_0)^2 + 4|f|^2}, -2f^* \right\}. \tag{273}$$

By using Ineq. (252), we have $f_\perp \geq E_\perp$, and hence the assumption (268) implies $f_\perp - f_0 \geq 0$. We thus obtain

$$\frac{|\zeta_2|}{\zeta_1} = \frac{2|f|/(f_\perp - f_0)}{1 + \sqrt{1 + 4|f|^2/(f_\perp - f_0)^2}} \leq \frac{|f|}{f_\perp - f_0} \leq \frac{\|\tilde{H}_{\rm t}|0_{\rm t}\rangle\|}{E_\perp - \langle 0_{\rm t}|\tilde{H}_{\rm t}|0_{\rm t}\rangle}, \tag{274}$$

where we use $|f| = |\langle 0_{\rm t}|\tilde{H}_{\rm t}|\phi_0\rangle| \leq \|\tilde{H}_{\rm t}|0_{\rm t}\rangle\|$.

From the equation $\zeta_1^2 + |\zeta_2|^2 = 1$, we obtain

$$\zeta_1 = \frac{1}{\sqrt{1 + |\zeta_2/\zeta_1|^2}} \geq 1 - \frac{1}{2}\left|\frac{\zeta_2}{\zeta_1}\right|^2. \tag{275}$$

On the other hand, we have

$$\||\tilde{0}_{\rm t}\rangle - |0_{\rm t}\rangle\|^2 = (\zeta_1 - 1)^2 + |\zeta_2|^2 = 2 - 2\zeta_1, \tag{276}$$

where we use the fact that $\zeta_1 \in \mathbb{R}$ and $\zeta_1 \geq 0$. By combining the inequalities (274), (275) and (276), we obtain

$$\||\tilde{0}_{\rm t}\rangle - |0_{\rm t}\rangle\|^2 \leq \left|\frac{\zeta_2}{\zeta_1}\right|^2 \leq \left(\frac{\|\tilde{H}_{\rm t}|0_{\rm t}\rangle\|}{E_\perp - \langle 0_{\rm t}|\tilde{H}_{\rm t}|0_{\rm t}\rangle}\right)^2, \tag{277}$$

which reduces to the inequality (269). This completes the proof. □

B. Outline of the proof

We here prove Theorem 5. For the proof, we need to derive the following two proposition. The first proposition is related to the energy distribution of the subsystem $B_s \subset \Lambda$ under the condition that the total energy is involved in an interval $(-\infty, E]$. We can prove the exponential decay of the distribution of h_s:

Proposition 8. *The overlap between the projections $\Pi^{(s)}_{>E'}$ and $\Pi_{\leq E}$ is bounded from above by*

$$\left\|\Pi^{(s)}_{>E'}\Pi_{\leq E}\right\| \leq \frac{4e^{3/2}}{e-1} e^{-\lambda(\delta E'_s - \delta E - 4g_0)} \tag{278}$$

with

$$\lambda := \frac{1}{12gk^2 + 4g_0}, \tag{279}$$

where $\delta E'_s := E' - E_{s,0}$, $\delta E := E - E_{\rm t,0}$ with $E_{s,0}$ and $E_{\rm t,0}$ the ground-state energies of h_s and $H_{\rm t}$, respectively. A similar inequality is satisfied for $\tilde{\Pi}_{\leq E}$:

$$\left\|\Pi^{(s)}_{>E'}\tilde{\Pi}_{\leq E}\right\| \leq \frac{4e^{3/2}}{e-1} e^{-\lambda'(\min(E', \tau_s) - E_{s,0} - \delta\tilde{E} - 4g_0)} \tag{280}$$

with $\delta\tilde{E} := E - \tilde{E}_{\rm t,0}$ and

$$\lambda' := \min\left(\frac{1}{112g_0}, \frac{1}{12gk^2}\right). \tag{281}$$

Second, we prove the norm difference between $H_{\rm t}$ and $\tilde{H}_{\rm t}$ under the constraint that the total energy is involved in an interval $(-\infty, E]$:

Proposition 9. *Let us define τ_s such that $\tau_s - E_{s,0} = \tau$, where τ is a fixed constant. We then obtain*

$$\|(H_{\rm t} - \tilde{H}_{\rm t})\Pi_{\leq E}\| \leq \frac{27(q+2)}{\lambda} e^{-\lambda(\tau - \delta E - 4g_0)}, \tag{282}$$

where δE has been defined as $\delta E := E - E_{\rm t,0}$.

Remark. We can derive the similar inequality for $\|(H_{\rm t}-\tilde{H}_{\rm t})\tilde{\Pi}_{\le E}\|$. However, the inequality becomes rather weak as follow

$$\|(H_{\rm t}-\tilde{H}_{\rm t})\tilde{\Pi}_{\le E}\| \le \|H_{\rm t}\|(q+2)e^{-\lambda'(\tau-\delta E)}, \tag{283}$$

which does not work in the thermodynamic limit as $\|H_{\rm t}\| \to \infty$.

Before going to the proof, we give the upper bound of κ and $\|\tilde{H}_{\rm t}|0_{\rm t}\rangle\|$. From the definition (251) of κ, we have

$$\kappa := \sum_{s=0}^{q+1} \left\| \Pi^{(s)}_{>\tau_s} \tilde{\Pi}_{\le \tilde{E}_{{\rm t},1}} \right\|. \tag{284}$$

We now obtain

$$\|\Pi^{(s)}_{>\tau_s} \tilde{\Pi}_{\le \tilde{E}_{{\rm t},1}}\| \le \frac{4e^{3/2}}{e-1} e^{-\lambda'(\tau_s - E_{s,0} - \tilde{\Delta}_{\rm t} - 4g_0)} = \frac{4e^{3/2}}{e-1} e^{-\lambda'(\tau - \tilde{\Delta}_{\rm t} - 4g_0)}, \tag{285}$$

where we use the inequality (280) with $E = \tilde{E}_{{\rm t},1}$ and $E' = \tau_s$. Note that $\tau_s = E_{s,0} + \tau$ and $\tilde{\Delta}_{\rm t} = \tilde{E}_{{\rm t},1} - \tilde{E}_{{\rm t},0}$. Then, κ is bounded from above by

$$\kappa \le \frac{4e^{3/2}(q+2)}{e-1} e^{-\lambda'(\tau - \tilde{\Delta}_{\rm t} - 4g_0)} \le 11(q+2)e^{-\lambda'(\tau - 8g_0)}, \tag{286}$$

where we use $\tilde{\Delta}_{\rm t} \le 4g_0$ in Lemma 13. Also, by using Proposition 9 with $E = 0$, we obtain

$$\|(H_{\rm t} - \tilde{H}_{\rm t})|0_{\rm t}\rangle\| = \|\tilde{H}_{\rm t}|0_{\rm t}\rangle\| \le \frac{27(q+2)}{\lambda} e^{-\lambda(\tau - 4g_0)}, \tag{287}$$

where we use $H_{\rm t}|0_{\rm t}\rangle = 0$ in the first equation.

Under the assumption of (227), we can obtain by straightforward calculations

$$\kappa \le \frac{\Delta_{\rm t}}{8g_0(q+1)} \le \frac{1}{12}, \tag{288}$$

and

$$\|\tilde{H}_{\rm t}|0_{\rm t}\rangle\| \le \frac{\Delta_{\rm t}}{16}, \tag{289}$$

where in the first inequality we use $g_0 \ge 1$ (Assumption 1), $q \ge 2$ and $\Delta_{\rm t} \le 2$. In particular, from the inequality (288), we have

$$E_\perp = \Delta_{\rm t}(1-\kappa)^2 - 2g_0\kappa(1+\kappa)(q+1) \ge \frac{41}{72}\Delta_{\rm t}, \tag{290}$$

where $E_\perp$ has been defined in Eq. (250).

We now have all the ingredients to prove the main inequalities in Theorem 5. First, from Lemmas 14 and 15, we obtain

$$\tilde{\Delta}_{\rm t} \ge \langle\psi|\tilde{H}_{\rm t}|\psi\rangle - \langle 0_{\rm t}|\tilde{H}_{\rm t}|0_{\rm t}\rangle \ge E_\perp - \|\tilde{H}_{\rm t}|0_{\rm t}\rangle\|. \tag{291}$$

By applying the upper bounds of (289) and (290) to the above inequality, we prove the inequality (229). In addition, from Lemma 16, we have

$$\||\tilde{0}_{\rm t}\rangle - |0_{\rm t}\rangle\| \le \frac{\|\tilde{H}_{\rm t}|0_{\rm t}\rangle\|}{E_\perp - \langle 0_{\rm t}|\tilde{H}_{\rm t}|0_{\rm t}\rangle} \le \frac{\|\tilde{H}_{\rm t}|0_{\rm t}\rangle\|}{E_\perp - \|\tilde{H}_{\rm t}|0_{\rm t}\rangle\|}. \tag{292}$$

By applying the inequalities (287), (289) and (290) to the above inequality, we have

$$\||\tilde{0}_{\rm t}\rangle - |0_{\rm t}\rangle\| \le \frac{1}{\frac{41}{72}\Delta_{\rm t} - \frac{1}{16}\Delta_{\rm t}} \frac{27(q+2)}{\lambda} e^{-\lambda(\tau-4g_0)} \le \frac{54(q+2)}{\lambda\Delta_{\rm t}} e^{-\lambda(\tau-4g_0)}. \tag{293}$$

This gives the proof of Ineq. (174).

This completes the proof of Theorem 5. □

C. Proof of Proposition 9 by utilizing Proposition 8

We first prove the Proposition 9 by assuming Proposition 8. We will prove Proposition 8 afterward. From the definition of the effective Hamiltonian (232), we calculate $\|(H_{\rm t}-\tilde{H}_{\rm t})\Pi_{\le E}\|$ as

$$\|(H_{\rm t}-\tilde{H}_{\rm t})\Pi_{\le E}\| \le \sum_{s=0}^{q+1} \left\|(h_s-\tau_s)\Pi^{(s)}_{>\tau_s}\Pi_{\le E}\right\|. \tag{294}$$

Hence, we need to estimate the upper bound of $\|(h_s-\tau_s)\Pi^{(s)}_{>\tau_s}\Pi_{\le E}\|$. For a given $y\in\mathbb{R}^+$ which we set afterward, we have

$$\begin{aligned}\left\|(h_s-\tau_s)\Pi^{(s)}_{>\tau_s}\Pi_{\le E}\right\| &= \left\|\sum_{j=0}^{\infty}(h_s-\tau_s)\Pi^{(s)}_{(\tau_s+jy,\tau_s+(j+1)y]}\Pi_{\le E}\right\| \\ &\le \sum_{j=0}^{\infty}\left\|(h_s-\tau_s)\Pi^{(s)}_{(\tau_s+jy,\tau_s+(j+1)y]}\right\|\cdot\left\|\Pi^{(s)}_{(\tau_s+jy,\tau_s+(j+1)y]}\Pi_{\le E}\right\| \\ &\le \sum_{j=0}^{\infty}(j+1)y\cdot\left\|\Pi^{(s)}_{>\tau_s+jy}\Pi_{\le E}\right\|,\end{aligned} \tag{295}$$

where in the last inequality we use

$$\left\|\Pi^{(s)}_{(\tau_s+jy,\tau_s+(j+1)y]}\Pi_{\le E}\right\| \le \left\|\Pi^{(s)}_{(\tau_s+jy,\infty)}\Pi_{\le E}\right\| = \left\|\Pi^{(s)}_{>\tau_s+jy}\Pi_{\le E}\right\|. \tag{296}$$

Thus, we can calculate the upper bound of $\left\|(h_s-\tau_s)\Pi^{(s)}_{>\tau_s}\Pi_{\le E}\right\|$ by estimating the norm of

$$\left\|\Pi^{(s)}_{>E'}\Pi_{\le E}\right\|. \tag{297}$$

By using the Proposition 8, we obtain

$$\|\Pi^{(s)}_{>\tau_s+jy}\Pi_{\le E}\| \le \frac{4e^{3/2}}{e-1}e^{-\lambda(\tau_s+jy-E_{s,0}-\delta E-4g_0)} = \frac{4e^{3/2}}{e-1}e^{-\lambda(\tau-\delta E-4g_0)}e^{-\lambda jy}, \tag{298}$$

where we use the definition $\tau_s := \tau + E_{s,0}$ for each of $s\in\{0,1,2,\dots,q+1\}$. It reduces the inequality (295) to

$$\begin{aligned}\left\|(h_s-\tau_s)\Pi^{(s)}_{>\tau_s}\Pi_{\le E}\right\| &\le \frac{4e^{3/2}}{e-1}e^{-\lambda(\tau-\delta E-4g_0)}\sum_{j=0}^{\infty}y(j+1)e^{-\lambda jy} \\ &= \frac{4e^{3/2}}{e-1}e^{-\lambda(\tau-\delta E-4g_0)}\frac{y\cdot e^{2\lambda y}}{(e^{\lambda y}-1)^2}.\end{aligned} \tag{299}$$

By choosing $y=1/\lambda$, we obtain

$$\left\|(h_s-\tau_s)\Pi^{(s)}_{>\tau_s}\Pi_{\le E}\right\| \le \frac{4e^{3/2}}{e-1}\cdot\frac{e^2}{\lambda(e-1)^2}e^{-\lambda(\tau-\delta E-4g_0)} \le \frac{27}{\lambda}e^{-\lambda(\tau-\delta E-4g_0)}. \tag{300}$$

Finally, by applying the above inequality to (294), we obatain

$$\|(H_{\rm t}-\tilde{H}_{\rm t})\Pi_{\le E}\| \le \frac{27(q+2)}{\lambda}e^{-\lambda(\tau-\delta E-4g_0)}. \tag{301}$$

This completes the proof of Proposition 9. □

D. Proof of Proposition 8: the first part (278)

For the proof, we first consider a normalized quantum state $|\psi\rangle$ and construct the following quantum state $|\phi\rangle$:

$$|\phi\rangle := \Pi^{(s)}_{>E'}\Pi_{\le E}|\psi\rangle. \tag{302}$$

Note that this state $|\phi\rangle$ may not be normalized. The norm $\|\Pi^{(s)}_{>E'}\Pi_{\le E}\|$ is now given by

$$\|\Pi^{(s)}_{>E'}\Pi_{\le E}\| = \sup_{|\psi\rangle}\|\phi\|, \tag{303}$$

where $\|\phi\|$ denotes the norm of the state $|\phi\rangle$. We then utilize the following inequality which we will prove below:

$$\|\phi\| \le \frac{4e^{3/2}}{e-1} e^{-\lambda(\langle H_{\rm t}\rangle_\phi - E)} \tag{304}$$

with

$$\langle H_{\rm t}\rangle_\phi = \frac{\langle \phi|H_{\rm t}|\phi\rangle}{\|\phi\|^2}, \tag{305}$$

where $\lambda := 1/(12gk^2 + 4g_0)$ as in Eq. (279).

In order to obtain an upper bound of $\|\phi\|^2$ from (304), we have to calculate a lower bound of $\langle H_{\rm t}\rangle_\phi$:

$$\langle H_{\rm t}\rangle_\phi = \langle h_s\rangle_\phi + \langle (h_{s,s+1} + h_{s-1,s})\rangle_\phi + \langle \delta H_s\rangle_\phi, \tag{306}$$

where we define $\delta H_s := H_{\rm t} - h_s - h_{s,s+1} - h_{s-1,s}$, which acts on the sites $\Lambda_s := \Lambda \setminus B_s$. We denote the ground state and the ground-state energy of δH_s by $|E_{\Lambda_s,0}\rangle$ and $E_{\Lambda_s,0}$, respectively. From the definition of $|\phi\rangle$, we obtain

$$\begin{aligned}
&\langle h_s\rangle_\phi = \frac{1}{\|\phi\|^2}\langle \psi|\Pi_{\le E}\Pi^{(s)}_{>E'} h_s \Pi^{(s)}_{>E'}\Pi_{\le E}|\psi\rangle \ge E', \\
&\langle (h_{s,s+1} + h_{s-1,s})\rangle_\phi \ge -(\|h_{s,s+1}\| + \|h_{s-1,s}\|) \ge -2g_0, \\
&\langle \delta H_s\rangle_\phi \ge E_{\Lambda_s,0} \ge E_{{\rm t},0} - E_{s,0} - 2g_0,
\end{aligned} \tag{307}$$

where the last inequality is derived from

$$\begin{aligned}
E_{{\rm t},0} \le& \big(\langle E_{s,0}| \otimes \langle E_{\Lambda_s,0}|\big) H_{\rm t} \big(|E_{s,0}\rangle \otimes |E_{\Lambda_s,0}\rangle\big) \\
\le& E_{s,0} + E_{\Lambda_s,0} + \|h_{s,s+1}\| + \|h_{s-1,s}\| \le E_{s,0} + E_{\Lambda_s,0} + 2g_0.
\end{aligned} \tag{308}$$

The inequalities in (307) imply the lower bound of $\langle H_{\rm t}\rangle_\phi$ in (306) as follows:

$$\langle H_{\rm t}\rangle_\phi \ge E_{{\rm t},0} + E' - E_{s,0} - 4g_0. \tag{309}$$

By applying the inequality (304) with (309) to Eq. (303), we prove the inequality (278).

[Proof of the inequality (304)]

We, in the following, prove the inequality (304). We start from the following equality:

$$\langle \phi|H_{\rm t}|\phi\rangle = \langle \phi|\Pi_{\le x} H_{\rm t} \Pi_{\le x}|\phi\rangle + \sum_{j=1}^\infty \langle \phi|\Pi_{[x+(j-1)y,x+jy)} H_{\rm t} \Pi_{[x+(j-1)y,x+jy)}|\phi\rangle, \tag{310}$$

where x and y are parameters which we set afterward. We here obtain

$$\begin{aligned}
\langle \phi|H_{\rm t}|\phi\rangle &\le x\|\Pi_{<x}|\phi\rangle\|^2 + \sum_{j=1}^\infty (x+jy)\|\Pi_{[x+(j-1)y,x+jy)}|\phi\rangle\|^2 \\
&= x\left(\|\Pi_{<x}|\phi\rangle\|^2 + \sum_{j=1}^\infty \|\Pi_{[x+(j-1)y,x+jy)}|\phi\rangle\|^2\right) + y\sum_{j=1}^\infty j\|\Pi_{[x+(j-1)y,x+jy)}|\phi\rangle\|^2 \\
&= x\|\phi\|^2 + y\sum_{j=1}^\infty j\|\Pi_{[x+(j-1)y,x+jy)}|\phi\rangle\|^2.
\end{aligned} \tag{311}$$

The definition of $|\phi\rangle$ in Eq. (302) implies

$$\begin{aligned}
\|\Pi_{[x+(j-1)y,x+jy)}|\phi\rangle\|^2 &= \|\Pi_{[x+(j-1)y,x+jy)}\Pi^{(s)}_{>E'}\Pi_{\le E}|\psi\rangle\|^2 \\
&\le \|\Pi_{[x+(j-1)y,x+jy)}\Pi^{(s)}_{>E'}\Pi_{\le E}\|^2.
\end{aligned} \tag{312}$$

In order to obtain the upper bound of $\|\Pi_{[x+(j-1)y,x+jy)}\Pi^{(s)}_{>E'}\Pi_{\le E}\|^2$, we utilize the following lemma (see Supplementary Note 4 D 1 for the proof):

Lemma 17. *Let O_s be an arbitrary operator such that $[O_s, h_s] = 0$. Then, we have*

$$\|\Pi_{\ge E'} O_s \Pi_{\le E}\| \le 4\|O_s\| \cdot e^{-\lambda(E'-E)} \tag{313}$$

with $\lambda := 1/(12gk^2 + 4g_0)$.

By choosing $O_s = \Pi^{(s)}_{\ge E'}$ in Lemma 17, we have $[\Pi^{(s)}_{\ge E'}, h_s] = 0$ from the definition (231). Hence, the inequality (313) implies

$$\|\Pi_{[x+(j-1)y,x+jy)}\Pi^{(s)}_{>E'}\Pi_{\le E}\|^2 \le 16e^{-2\lambda\left[x-E+(j-1)y\right]}, \tag{314}$$

where we use the fact that $\|\Pi^{(s)}_{\ge E'}\| = 1$. By the use of this inequality, we have from (312)

$$\sum_{j=1}^{\infty} j\|\Pi_{[x+(j-1)y,x+jy)}|\phi\rangle\|^2 \le 16e^{-2\lambda(x-E)}\sum_{j=1}^{\infty} je^{-2\lambda(j-1)y}$$
$$= 16e^{-2\lambda(x-E)}\frac{e^{4y\lambda}}{(e^{2y\lambda}-1)^2}. \tag{315}$$

This inequality reduces the inequality (311) to

$$\langle\phi|H_{\mathrm{t}}|\phi\rangle \le x\|\phi\|^2 + 16y\cdot e^{-2\lambda(x-E)}\frac{e^{4y\lambda}}{(e^{2y\lambda}-1)^2}. \tag{316}$$

By choosing $y = 1/(2\lambda)$, we obtain

$$\langle\phi|H_{\mathrm{t}}|\phi\rangle \le x\|\phi\|^2 + \frac{8e^2}{\lambda(e-1)^2}e^{-2\lambda(x-E)}. \tag{317}$$

From the definition (305), we have $\langle\phi|H_{\mathrm{t}}|\phi\rangle = \|\phi\|^2\cdot\langle H_{\mathrm{t}}\rangle_\phi$, which reduces the above inequality to

$$\|\phi\|^2 \le \frac{8e^2}{\lambda(e-1)^2(\langle H_{\mathrm{t}}\rangle_\phi - x)}e^{-2\lambda(x-E)}. \tag{318}$$

By choosing x so that $\langle H_{\mathrm{t}}\rangle_\phi - x = 1/(2\lambda)$, we finally obtain

$$\|\phi\| \le \frac{4e^{3/2}}{e-1}e^{-\lambda(\langle H_{\mathrm{t}}\rangle_\phi - E)}. \tag{319}$$

This completes the proof. □

1. *Proof of Lemma 17*

In order to derive the lemma, we utilize the following additional lemma [12]:

Lemma 18 (Theorem 2.1 in Ref. [12]). *Let H be an arbitrary Hamiltonian in the form of*

$$H = \sum_{Z:|Z|\le k} h_Z, \quad \sum_{Z:Z\ni i}\|h_Z\| \le g. \tag{320}$$

Then, for an arbitrary q-local operator $\Gamma^{(q)}$ (i.e., at most q-body interactions are included), we have

$$\|[H,\Gamma^{(q)}]\| \le 6gkq\|\Gamma^{(q)}\|. \tag{321}$$

In order to prove Lemma 17, we follow Ref. [7]. We start from the inequality

$$\|\Pi_{\ge E'}O_s\Pi_{\le E}\| = \left\|\Pi_{\ge E'}e^{-\nu H_{\mathrm{t}}}e^{\nu H_{\mathrm{t}}}O_s e^{-\nu H_{\mathrm{t}}}e^{\nu H_{\mathrm{t}}}\Pi_{\le E}\right\| \le e^{-\nu(E'-E)}\left\|e^{\nu H_{\mathrm{t}}}O_s e^{-\nu H_{\mathrm{t}}}\right\|. \tag{322}$$

Then, we need to upper-bound

$$\left\|e^{\nu H_{\mathrm{t}}}O_s e^{-\nu H_{\mathrm{t}}}\right\| \le \sum_{m=0}^{\infty}\frac{\nu^m}{m!}\|\mathrm{ad}^m_{H_{\mathrm{t}}}(O_s)\|, \tag{323}$$

where ad is the commutation operator, namely $\mathrm{ad}_{H_{\mathrm{t}}}(\cdot) := [H_{\mathrm{t}},\cdot]$. On the norm of $\|\mathrm{ad}^m_{H_{\mathrm{t}}}(O_s)\|$, we prove the following inequality:

$$\|\mathrm{ad}^m_{H_{\mathrm{t}}}(O_s)\| \le 2(6gk^2 + 2g_0)^m m!\|O_s\|. \tag{324}$$

For the proof of (324), we use the mathematical induction. For $m=1$, we prove the inequality (324) as follows:

$$\|\mathrm{ad}_{H_{\mathrm{t}}}(O_s)\| = \|[h_{s-1,s}+h_{s,s+1}, O_s]\| \le 2\|h_{s-1,s}+h_{s,s+1}\|\cdot\|O_s\| \le 4g_0\|O_s\| \le 2(6gk^2+2g_0)\|O_s\|. \tag{325}$$

We then assume the inequality (324) for $m \le m_0$ and consider the case of general m_0+1:

$$\begin{aligned}\|\mathrm{ad}_{H_{\mathrm{t}}}^{m_0+1}(O_s)\| &= \|\mathrm{ad}_{H_{\mathrm{t}}}^{m_0}\left([h_{s-1,s}+h_{s,s+1}, O_s]\right)\| \\ &\le 2\sum_{m_1+m_2=m_0}\|\mathrm{ad}_{H_{\mathrm{t}}}^{m_1}(O_s)\|\cdot\|\mathrm{ad}_{H_{\mathrm{t}}}^{m_2}\left(h_{s-1,s}+h_{s,s+1}\right)\|. \end{aligned}\tag{326}$$

By applying Lemma 18 to $\mathrm{ad}_{H_{\mathrm{t}}}^{m}(h_{s-1,s}+h_{s,s+1})$, we have

$$\|\mathrm{ad}_{H_{\mathrm{t}}}^{m}(h_{s-1,s}+h_{s,s+1})\| \le (6gk^2)^m m!\|h_{s-1,s}+h_{s,s+1}\| \le 2g_0(6gk^2)^m m!, \tag{327}$$

where we use the fact that $\mathrm{ad}_{H_{\mathrm{t}}}^{j}(h_{s-1,s}+h_{s,s+1})$ ($j\in\mathbb{N}$) has at most (jk)-body interactions. By applying the inequalities (324) and (327) to (326), we obtain

$$\begin{aligned}\|\mathrm{ad}_{H_{\mathrm{t}}}^{m_0+1}(O_s)\| &\le 4g_0\|O_s\|\sum_{m_1+m_2=m_0}(6gk^2+2g_0)^{m_1}(6gk^2)^{m_2}m_1!m_2! \\ &\le 2(6gk^2+2g_0)^{m_0+1}\|O_s\|m_0!\sum_{j=0}^{m_0}\frac{1}{\binom{m_0}{j}} \le 2(6gk^2+2g_0)^{m_0+1}\|O_s\|(m_0+1)!. \end{aligned}\tag{328}$$

This completes the proof of (324).

By applying the inequality (323) to (324), we obtain

$$\left\|e^{\nu H_{\mathrm{t}}}O_s e^{-\nu H_{\mathrm{t}}}\right\| \le 2\|O_s\|\sum_{m=0}^{\infty}[\nu(6gk^2+2g_0)]^m = \frac{2\|O_s\|}{1-\nu(6gk^2+2g_0)}, \tag{329}$$

which yields $\left\|e^{\nu H_{\mathrm{t}}}O_s e^{-\nu H_{\mathrm{t}}}\right\| \le 4\|O_s\|$ for $\nu = 1/(12gk^2+4g_0) =: \lambda$. Therefore, from (322), we prove Lemma 17. $\square$

E. Proof of Proposition 8: the second part (280)

We can prove the inequality (280) in the same way. We define $|\tilde{\phi}\rangle$ as follows:

$$|\tilde{\phi}\rangle := \Pi^{(s)}_{>E'}\tilde{\Pi}_{\le E}|\psi\rangle, \tag{330}$$

which implies

$$\|\Pi^{(s)}_{>E'}\tilde{\Pi}_{\le E}\| = \max_{|\psi\rangle}\|\tilde{\phi}\|, \tag{331}$$

where $\|\tilde{\phi}\|$ denotes the norm of the state $|\tilde{\phi}\rangle$. We then prove the following inequality similar to Ineq. (304):

$$\|\tilde{\phi}\| \le \frac{4e^{3/2}}{e-1}e^{-\lambda'(\langle\tilde{H}_{\mathrm{t}}\rangle_{\tilde{\phi}}-E)}, \tag{332}$$

with

$$\langle\tilde{H}_{\mathrm{t}}\rangle_{\tilde{\phi}} = \frac{\langle\tilde{\phi}|\tilde{H}_{\mathrm{t}}|\tilde{\phi}\rangle}{\|\tilde{\phi}\|^2}, \tag{333}$$

where λ' was defined in Eq. (281). We show the proof of the inequality (332) below.

We estimate a lower bound of $\langle\tilde{H}_{\mathrm{t}}\rangle_{\tilde{\phi}}$. We first obtain

$$\langle\tilde{H}_{\mathrm{t}}\rangle_{\tilde{\phi}} := \frac{\langle\tilde{\phi}|\tilde{H}_{\mathrm{t}}|\tilde{\phi}\rangle}{\|\tilde{\phi}\|^2} = \langle\tilde{h}_s\rangle_{\tilde{\phi}} + \langle(h_{s,s+1}+h_{s-1,s})\rangle_{\tilde{\phi}} + \langle\delta\tilde{H}_s\rangle_{\tilde{\phi}}, \tag{334}$$

where we define $\delta\tilde{H}_s := \tilde{H}_{\mathrm{t}} - \tilde{h}_s - h_{s,s+1} - h_{s-1,s}$. We here define the ground state and the ground-state energy of $\delta\tilde{H}_s$ as $|\tilde{E}_{\Lambda_s,0}\rangle$ and $\tilde{E}_{\Lambda_s,0}$, respectively. Note that $\delta\tilde{H}_s$ acts on the sites $\Lambda_s := \Lambda\setminus B_s$. We can obtain the similar inequalities to (307) as follows:

$$\begin{aligned}&\langle\tilde{h}_s\rangle_{\tilde{\phi}} \ge \frac{1}{\|\tilde{\phi}\|^2}\langle\psi|\tilde{\Pi}_{\le E}\Pi^{(s)}_{>E'}\tilde{h}_s\Pi^{(s)}_{>E'}\tilde{\Pi}_{\le E}|\psi\rangle \ge \min(E',\tau_s), \\ &\langle(h_{s,s+1}+h_{s-1,s})\rangle_{\tilde{\phi}} \ge -(\|h_{s,s+1}\|+\|h_{s-1,s}\|\|) \ge -2g_0, \\ &\langle\delta\tilde{H}_s\rangle_{\tilde{\phi}} \ge \tilde{E}_{\Lambda_s,0} \ge \tilde{E}_{\mathrm{t},0} - E_{s,0} - 2g_0, \end{aligned}\tag{335}$$

where the first inequality and the third inequality are derived from

$$\tilde{h}_s\Pi^{(s)}_{>E'} = \begin{cases} h_s\Pi^{(s)}_{(E',\tau_s)} + \tau_s\Pi^{(s)}_{[\tau_s,\infty)} & \text{for} \quad E' < \tau_s \\ \tau_s\Pi^{(s)}_{(E',\infty)} & \text{for} \quad E' \ge \tau_s. \end{cases} \tag{336}$$

and

$$\tilde{E}_{\mathrm{t},0} \le \big(\langle E_{s,0}| \otimes \langle \tilde{E}_{\Lambda_s,0}|\big)\tilde{H}_{\mathrm{t}}\big(|E_{s,0}\rangle \otimes |\tilde{E}_{\Lambda_s,0}\rangle\big) \le E_{s,0} + \tilde{E}_{\Lambda_s,0} + 2g_0. \tag{337}$$

We therefore obtain

$$\langle \tilde{H}_{\mathrm{t}}\rangle_{\tilde{\phi}} \ge \tilde{E}_{\mathrm{t},0} + \min(E',\tau_s) - E_{s,0} - 4g_0. \tag{338}$$

By combining the inequalities (332) and (338), we prove the inequality (280). □

[Proof of the inequality (332)]

We follow the same step as the proof of the inequality (304). We start from the following equality:

$$\langle\tilde{\phi}|\tilde{H}_{\mathrm{t}}|\tilde{\phi}\rangle = \langle\tilde{\phi}|\tilde{\Pi}_{\le x}\tilde{H}_{\mathrm{t}}\tilde{\Pi}_{\le x}|\tilde{\phi}\rangle + \sum_{j=1}^{\infty}\langle\tilde{\phi}|\tilde{\Pi}_{[x+(j-1)y,x+jy)}\tilde{H}_{\mathrm{t}}\tilde{\Pi}_{[x+(j-1)y,x+jy)}|\tilde{\phi}\rangle, \tag{339}$$

which yields

$$\begin{aligned}\langle\tilde{\phi}|\tilde{H}_{\mathrm{t}}|\tilde{\phi}\rangle &\le x\|\tilde{\Pi}_{\le x}|\tilde{\phi}\rangle\|^2 + \sum_{j=1}^{\infty}(x+jy)\|\tilde{\Pi}_{[x+(j-1)y,x+jy)}|\tilde{\phi}\rangle\|^2 \\ &= x\left(\|\tilde{\Pi}_{<x}|\phi\rangle\|^2 + \sum_{j=1}^{\infty}\|\tilde{\Pi}_{[x+(j-1)y,x+jy)}|\tilde{\phi}\rangle\|^2\right) + y\sum_{j=1}^{\infty}j\|\tilde{\Pi}_{[x+(j-1)y,x+jy)}|\tilde{\phi}\rangle\|^2 \\ &= x\|\tilde{\phi}\|^2 + y\sum_{j=1}^{\infty}j\|\tilde{\Pi}_{[x+(j-1)y,x+jy)}|\tilde{\phi}\rangle\|^2. \end{aligned} \tag{340}$$

From the definition of $|\tilde{\phi}\rangle$ as in Eq. (330), we have

$$\begin{aligned}\|\tilde{\Pi}_{[x+(j-1)y,x+jy)}|\tilde{\phi}\rangle\|^2 &= \|\tilde{\Pi}_{[x+(j-1)y,x+jy)}\Pi^{(s)}_{>E'}\tilde{\Pi}_{\le E}|\psi\rangle\|^2 \\ &\le \|\tilde{\Pi}_{[x+(j-1)y,x+jy)}\Pi^{(s)}_{>E'}\tilde{\Pi}_{\le E}\|^2. \end{aligned} \tag{341}$$

Now, the problem is to bound the norm $\|\tilde{\Pi}_{[x+(j-1)y,x+jy)}\Pi^{(s)}_{>E'}\tilde{\Pi}_{\le E}\|$ from above. Because the effective Hamiltonian $\tilde{H}_{\mathrm{t}}$ is no longer given in the form of k-local Hamiltonian as (320), we cannot use Lemma 17. Instead, we can prove the following proposition which is similar to Lemma 6.2 in Ref. [7]:

Proposition 10. *Let O_s be an arbitrary operator supported on the subset $B_s \subset \Lambda$ such that $[O_s, h_s] = 0$. Then, we have*

$$\|\tilde{\Pi}_{\ge E'}O_s\tilde{\Pi}_{\le E}\| \le 4\|O_s\| \cdot e^{-\lambda'(E'-E)}, \tag{342}$$

where $\lambda' := \min\left(\frac{1}{112g_0}, \frac{1}{12gk^2}\right)$.

By choosing $O_s = \Pi^{(s)}_{\ge E'}$ in Proposition 10, we obtain

$$\|\tilde{\Pi}_{[x+(j-1)y,x+jy)}\Pi^{(s)}_{>E'}\tilde{\Pi}_{\le E}\|^2 \le 16e^{-2\lambda'[x-E+(j-1)y]}, \tag{343}$$

where we use $\|\Pi^{(s)}_{\ge E'}\| = 1$. By the use of this inequality, we have from (341)

$$\sum_{j=1}^{\infty}j\|\tilde{\Pi}_{[x+(j-1)y,x+jy)}|\tilde{\phi}\rangle\|^2 \le 16e^{-2\lambda'(x-E)}\sum_{j=1}^{\infty}je^{-2\lambda'(j-1)y} = 16e^{-2\lambda'(x-E)}\frac{e^{4y\lambda'}}{(e^{2y\lambda'}-1)^2}. \tag{344}$$

This inequality reduces the inequality (340) to

$$\langle\tilde{\phi}|\tilde{H}_{\mathrm{t}}|\tilde{\phi}\rangle \le x\|\tilde{\phi}\|^2 + 16y \cdot e^{-2\lambda'(x-E)}\frac{e^{4y\lambda'}}{(e^{2y\lambda'}-1)^2}. \tag{345}$$

By choosing $y = 1/(2\lambda')$, we obtain

$$\langle\tilde{\phi}|\tilde{H}_{\rm t}|\tilde{\phi}\rangle \le x\|\tilde{\phi}\|^2 + \frac{8e^2}{\lambda'(e-1)^2} e^{-2\lambda'(x-E)}. \tag{346}$$

From the definition (333), we have $\langle\tilde{\phi}|\tilde{H}_{\rm t}|\tilde{\phi}\rangle = \|\tilde{\phi}\|^2 \cdot \langle\tilde{H}_{\rm t}\rangle_\phi$, which reduces the above inequality to

$$\|\tilde{\phi}\|^2 \le \frac{8e^2}{\lambda'(e-1)^2(\langle\tilde{H}_{\rm t}\rangle_{\tilde{\phi}} - x)} e^{-2\lambda'(x-E)}. \tag{347}$$

By choosing x so that $\langle\tilde{H}_{\rm t}\rangle_{\tilde{\phi}} - x = 1/(2\lambda')$, we finally obtain

$$\|\tilde{\phi}\| \le \frac{4e^{3/2}}{e-1} e^{-\lambda'(\langle\tilde{H}_{\rm t}\rangle_{\tilde{\phi}} - E)}. \tag{348}$$

This completes the proof. □

F. Proof of Proposition 10

We start from the following inequality:

$$\begin{aligned}\|\tilde{\Pi}_{\ge E'} O_s \tilde{\Pi}_{\le E}\| &= \left\|\tilde{\Pi}_{\ge E'} e^{-\nu\tilde{H}_{\rm t}} e^{\nu\tilde{H}_{\rm t}} O_s e^{-\nu\tilde{H}_{\rm t}} e^{\nu\tilde{H}_{\rm t}} \tilde{\Pi}_{\le E}\right\| \\ &\le e^{-\nu(E'-E)} \left\|e^{\nu\tilde{H}_{\rm t}} O_s e^{-\nu\tilde{H}_{\rm t}}\right\|,\end{aligned} \tag{349}$$

where ν is a parameter satisfying $\nu \le 1/(12gk^2)$ which is determined afterward. We then decompose $\tilde{H}_{\rm t}$ as $\tilde{H}_{\rm t} = G + F$:

$$G = \sum_{s=0}^{q+1} \tilde{h}_s, \quad F = \sum_{s=0}^{q} h_{s,s+1}. \tag{350}$$

By using the above decomposition, we have

$$e^{\nu\tilde{H}_{\rm t}} = \mathcal{T}_{\rightarrow}\left[e^{\int_0^\nu F(x)dx}\right] e^{\nu G}, \quad e^{-\nu\tilde{H}_{\rm t}} = e^{-\nu G}\mathcal{T}_{\leftarrow}\left[e^{-\int_0^\nu F(x)dx}\right], \tag{351}$$

where $F(x) := e^{xG} F e^{-xG}$ and $\mathcal{T}_{\rightarrow}$, $\mathcal{T}_{\leftarrow}$ are the ordering operator:

$$\begin{aligned}\mathcal{T}_{\rightarrow}\left[F(x_1)F(x_2)\cdots F(x_m)\right] &= F(x_{p_1})F(x_{p_2})\cdots F(x_{p_m}), \\ \mathcal{T}_{\leftarrow}\left[F(x_1)F(x_2)\cdots F(x_m)\right] &= F(x_{p_m})F(x_{p_{m-1}})\cdots F(x_{p_1}),\end{aligned} \tag{352}$$

with p a permutation such that $x_{p_1} \le x_{p_2} \le \cdots \le x_{p_m}$. From the above expressions, we obtain

$$e^{\nu\tilde{H}_{\rm t}} O_s e^{-\nu\tilde{H}_{\rm t}} = \mathcal{T}_{\rightarrow}\left[e^{\int_0^\nu F(x)dx}\right] e^{\nu G} O_s e^{-\nu G}\mathcal{T}_{\leftarrow}\left[e^{\int_0^\nu F(x)dx}\right]. \tag{353}$$

Because the operator O_s is supported on the subset $B_s \subset \Lambda$ and satisfies $[O_s, \tilde{h}_s] = 0$, we have $[O_s, G] = 0$ and hence

$$e^{\nu G} O_s e^{-\nu G} = O_s. \tag{354}$$

Also, from $[\tilde{h}_s, \tilde{h}_{s'}] = 0$ for $\forall s, s'$, we obtain

$$\begin{aligned}&F(x) = \sum_{s=0}^{q} h_{s,s+1}(x), \\ &h_{s,s+1}(x) = e^{xG} h_{s,s+1} e^{-xG} = e^{x(\tilde{h}_s + \tilde{h}_{s+1})} h_{s,s+1} e^{-x(\tilde{h}_s + \tilde{h}_{s+1})},\end{aligned} \tag{355}$$

where we use the fact that $[h_{s,s+1}, \tilde{h}_{s'}] = 0$ as long as $s' \ne s, s+1$. Therefore, $h_{s,s+1}(x)$ is still supported on the subset $B_s \sqcup B_{s+1} \subset \Lambda$. We now define

$$\mathfrak{g} := \sup_{\substack{0<x<\nu \\ s\in\{0,1,\ldots,q\}}} \|h_{s,s+1}(x)\|. \tag{356}$$

In order to calculate $\mathfrak{g}$ in Eq. (356), we need to consider the norm of

$$h_{s,s+1}(x) = e^{x\tilde{h}_s} e^{x\tilde{h}_{s+1}} h_{s,s+1} e^{-x\tilde{h}_{s+1}} e^{-x\tilde{h}_s}. \tag{357}$$

As we will prove in Supplementary Note 4 F 1, we prove the following inequality:

$$\|e^{x\tilde{h}_s} e^{x\tilde{h}_{s+1}} h_{s,s+1} e^{-x\tilde{h}_{s+1}} e^{-x\tilde{h}_s}\| \le 28 g_0. \tag{358}$$

under the condition of

$$x \le \nu \le \frac{1}{12gk^2}. \tag{359}$$

From the inequality (358), we obtain $\mathfrak{g} = 28g_0$.

Then, we consider the Baker-Campbell-Hausdorff expansion as follows:

$$e^{\nu \tilde{H}_{\rm t}} O_s e^{-\nu \tilde{H}_{\rm t}} = \sum_{m=0}^{\infty} \int_0^{\nu} dx_1 \int_{x_1}^{\nu} dx_2 \cdots \int_{x_{m-1}}^{\nu} {\rm ad}_{F(x_1)} {\rm ad}_{F(x_2)} \cdots {\rm ad}_{F(x_m)}(O_s) dx_m, \tag{360}$$

where ad is the commutation operator, namely ${\rm ad}_{F(x)}(\cdot) := [F(x), \cdot]$. By using Eq. (356), the norm of the commutators are bounded from above by

$$\begin{aligned} &\|{\rm ad}_{F(x)}(O_s)\| \le \|{\rm ad}_{h_{s,s+1}(x)}(O_s)\| + \|{\rm ad}_{h_{s-1,s}(x)}(O_s)\| \le 2(\|h_{s,s+1}(x)\| + \|h_{s-1,s}(x)\|)\|O_s\| \le 4\mathfrak{g}\|O_s\|, \\ &\|{\rm ad}_{F(x_1)}{\rm ad}_{F(x_2)}(O_s)\| \le 2^2 \cdot 3\mathfrak{g}(2\mathfrak{g}\|O_s\|), \quad \|{\rm ad}_{F(x_1)}{\rm ad}_{F(x_2)}{\rm ad}_{F(x_3)}(O_s)\| \le 2^3 \cdot 4\mathfrak{g}[3\mathfrak{g}(2\mathfrak{g}\|O_s\|)], \end{aligned} \tag{361}$$

which is generalized as

$$\|{\rm ad}_{F(x_1)}{\rm ad}_{F(x_2)} \cdots {\rm ad}_{F(x_m)}(O_s)\| \le (m+1)!(2\mathfrak{g})^m \|O_s\|. \tag{362}$$

By combining the inequality (362) with Eq. (360), we obtain the upper bound of

$$\|e^{\nu \tilde{H}_{\rm t}} O_s e^{-\nu \tilde{H}_{\rm t}}\| \le \sum_{m=0}^{\infty} \frac{\nu^m}{m!}(m+1)!(2\mathfrak{g})^m \|O_s\| = \frac{\|O_s\|}{(1-2\mathfrak{g}\nu)^2}. \tag{363}$$

Also, by choosing ν such that $2\mathfrak{g}\nu \le 1/2$, we have $\|e^{\nu \tilde{H}_{\rm t}} O_s e^{-\nu \tilde{H}_{\rm t}}\| \le 4\|O_s\|$ and reduce the inequality (349) to

$$\|\tilde{\Pi}_{\ge E'} O_s \tilde{\Pi}_{\le E}\| \le 4\|O_s\| e^{-\nu(E'-E)}. \tag{364}$$

This is the inequality which we aim to prove. Because of $\mathfrak{g} = 28g_0$, under the assumption of $\nu \le 1/(12gk^2)$, the condition $2\mathfrak{g}\nu \le 1/2$ is satisfied for

$$\nu = \lambda' := \min\left(\frac{1}{112g_0}, \frac{1}{12gk^2}\right). \tag{365}$$

This completes the proof of Proposition 10. □

1. *Proof of the inequality* (358)

For the proof, we start from $\|e^{x\tilde{h}_s} O e^{-x\tilde{h}_s}\|$ for an arbitrary operator O. From the definition of $\tilde{h}_s$, the operator $e^{x\tilde{h}_s}$ is decomposed as follows:

$$e^{x\tilde{h}_s} = \Pi^{(s)}_{<\tau_s} e^{xh_s} + \Pi^{(s)}_{\ge\tau_s} e^{x\tau_s}, \tag{366}$$

where we utilized the equalities $\tilde{h}_s \Pi^{(s)}_{<\tau_s} = h_s \Pi^{(s)}_{<\tau_s}$ and $\tilde{h}_s \Pi^{(s)}_{\ge\tau_s} = \tau_s \Pi^{(s)}_{\ge\tau_s}$. We then have

$$\begin{aligned} e^{x\tilde{h}_s} O e^{-x\tilde{h}_s} =& \Pi^{(s)}_{\ge\tau_s} O \Pi^{(s)}_{\ge\tau_s} + \Pi^{(s)}_{<\tau_s} e^{xh_s} O e^{-xh_s} \Pi^{(s)}_{<\tau_s} \\ &+ \Pi^{(s)}_{<\tau_s} e^{x(h_s-\tau_s)} O \Pi^{(s)}_{\ge\tau_s} + \Pi^{(s)}_{\ge\tau_s} e^{x\tau_s} O e^{-xh_s} \Pi^{(s)}_{<\tau_s}. \end{aligned} \tag{367}$$

The norms of the four terms are bounded from above by

$$\begin{aligned} \|\Pi^{(s)}_{\ge\tau_s} O \Pi^{(s)}_{\ge\tau_s}\| &\le \|O\|, \\ \|\Pi^{(s)}_{<\tau_s} e^{xh_s} O e^{-xh_s} \Pi^{(s)}_{<\tau_s}\| &\le \|e^{xh_s} O e^{-xh_s}\|, \\ \|\Pi^{(s)}_{<\tau_s} e^{x(h_s-\tau_s)} O \Pi^{(s)}_{\ge\tau_s}\| &\le \|\Pi^{(s)}_{<\tau_s} e^{x(h_s-\tau_s)}\| \cdot \|O \Pi^{(s)}_{\ge\tau_s}\| \le \|O\|, \\ \|\Pi^{(s)}_{\ge\tau_s} e^{x\tau_s} O_X e^{-xh_s} \Pi^{(s)}_{<\tau_s}\| &= \|\Pi^{(s)}_{\ge\tau_s} e^{x\tau_s} e^{-xh_s} e^{xh_s} O e^{-xh_s} \Pi^{(s)}_{<\tau_s}\| \\ &\le \|\Pi^{(s)}_{\ge\tau_s} e^{x(\tau_s-h_s)}\| \cdot \|e^{xh_s} O e^{-xh_s}\| \le \|e^{xh_s} O e^{-xh_s}\|. \end{aligned} \tag{368}$$

By combining the above two inequalities (367) and (368), we obtain

$$\|e^{x\tilde{h}_s} O e^{-x\tilde{h}_s}\| \le 2\|O\| + 2\|e^{xh_s} O e^{-xh_s}\|. \tag{369}$$

From the inequality (369), we obtain

$$\|e^{x\tilde{h}_{s+1}} e^{x\tilde{h}_s} h_{s,s+1} e^{-x\tilde{h}_s} e^{-x\tilde{h}_{s+1}}\| \le 2\|e^{x\tilde{h}_s} h_{s,s+1} e^{-x\tilde{h}_s}\| + 2\|e^{xh_{s+1}} e^{x\tilde{h}_s} h_{s,s+1} e^{-x\tilde{h}_s} e^{-xh_{s+1}}\|, \tag{370}$$

where we set $O = e^{x\tilde{h}_s} h_{s,s+1} e^{-x\tilde{h}_s}$ in (369). Furthermore, the inequality (369) gives

$$\|e^{x\tilde{h}_s} h_{s,s+1} e^{-x\tilde{h}_s}\| \le 2\|h_{s,s+1}\| + 2\|e^{xh_s} h_{s,s+1} e^{-xh_s}\|, \tag{371}$$

and

$$\begin{aligned} \|e^{xh_{s+1}} e^{x\tilde{h}_s} h_{s,s+1} e^{-x\tilde{h}_s} e^{-xh_{s+1}}\| &= \|e^{x\tilde{h}_s} e^{xh_{s+1}} h_{s,s+1} e^{-xh_{s+1}} e^{-x\tilde{h}_s}\| \\ &\le 2\|e^{xh_{s+1}} h_{s,s+1} e^{-xh_{s+1}}\| + 2\|e^{xh_s} e^{xh_{s+1}} h_{s,s+1} e^{-xh_{s+1}} e^{-xh_s}\|, \end{aligned} \tag{372}$$

where we use $[\tilde{h}_s, h_{s+1}] = 0$ which yields $e^{xh_{s+1}} e^{x\tilde{h}_s} = e^{x\tilde{h}_s} e^{xh_{s+1}}$. By applying the inequalities (371) and (372) to (370), we have

$$\begin{aligned} &\|e^{x\tilde{h}_{s+1}} e^{x\tilde{h}_s} h_{s,s+1} e^{-x\tilde{h}_s} e^{-x\tilde{h}_{s+1}}\| \\ \le& 4\|h_{s,s+1}\| + 4\|e^{xh_s} h_{s,s+1} e^{-xh_s}\| + 4\|e^{xh_{s+1}} h_{s,s+1} e^{-xh_{s+1}}\| + 4\|e^{xh_s} e^{xh_{s+1}} h_{s,s+1} e^{-xh_{s+1}} e^{-xh_s}\|. \end{aligned} \tag{373}$$

We here estimate an upper bound of each of the norms in (373). We first consider $\|e^{xh_s} h_{s,s+1} e^{-xh_s}\|$ by using the Baker-Campbell-Hausdorff expansion:

$$\|e^{xh_s} h_{s,s+1} e^{-xh_s}\| \le \sum_{m=0}^{\infty} \frac{x^m}{m!} \|\mathrm{ad}_{h_s}^m (h_{s,s+1})\|. \tag{374}$$

By applying Lemma 18 to $\mathrm{ad}_{h_s}^m (h_{s,s+1})$, we have

$$\|\mathrm{ad}_{h_s}^m (h_{s,s+1})\| \le (6gk^2)^m m! \|h_{s,s+1}\| \le g_0 (6gk^2)^m m!, \tag{375}$$

where we use $\|h_{s,s+1}\| \le g_0$ in (46). This inequality reduces (374) to

$$\|e^{xh_s} h_{s,s+1} e^{-xh_s}\| \le g_0 \sum_{m=0}^{\infty} (6gk^2 x)^m = g_0 \frac{1}{1 - 6gk^2 x} \le 2g_0, \tag{376}$$

where in the last inequality we use the condition of (359), namely $x \le \nu \le 1/(12gk^2)$. We obtain the same inequality for $\|e^{xh_{s+1}} h_{s,s+1} e^{-xh_{s+1}}\|$ and $\|e^{xh_s} e^{xh_{s+1}} h_{s,s+1} e^{-xh_{s+1}} e^{-xh_s}\|$. Notice that we can apply Lemma 18 to $h_s + h_{s+1}$, which allows us to obtain the upper bound of $\|e^{xh_s} e^{xh_{s+1}} h_{s,s+1} e^{-xh_{s+1}} e^{-xh_s}\| = \|e^{x(h_s + h_{s+1})} h_{s,s+1} e^{-x(h_s + h_{s+1})}\|$ in the same way of (376). Therefore, we finally obtain

$$\|e^{x\tilde{h}_{s+1}} e^{x\tilde{h}_s} h_{s,s+1} e^{-x\tilde{h}_s} e^{-x\tilde{h}_{s+1}}\| \le 28g_0. \tag{377}$$

This completes the proof. □

Supplementary Note 5. LIST OF NOTATIONS AND DEFINITIONS

We here give a list of definitions and notations which we use several times in the proof.

1. $\{|0\rangle, \Delta\}$ (Supplementary Note 1 A 1): the ground state and the spectral gap of the Hamiltonian H.
2. g [Eq. (5)]: upper bound of one-site energy, which is set to be equal to 1 by choosing the energy unit appropriately.
3. $V_{X,Y}(\Lambda_0)$ [Eq. (13)]: interaction operator between two subsystems $X \subset \Lambda$ and $Y \subset \Lambda$.
4. $\mathrm{SR}(O, X)$ and $\mathrm{SR}(|\psi\rangle, X)$ (Supplementary Note 1 A 3): Schmidt rank of operator and quantum state.
5. K [Eq. (40)]: approximate ground state projection (AGSP) for the ground state $|0\rangle$.
6. $|0_K\rangle$ [Eq. (41)]: quantum state which is invariant by AGSP K.
7. $\{\delta_K, \epsilon_K, D_K\}$ [Eq. (42)]: three parameters which characterize the AGSP operator K.

8. $\{B_s\}_{s=0}^{q+1}$ [Eq. (44), Supplementary Figure 2]: decomposed subsets which constitute the total system Λ. This decomposition has two parameters $q+2$ (the number of blocks) and l (the length of the bulk blocks, i.e., $B_1, B_2, \ldots, B_q$).

9. h_s [Eq. (45)]: internal interactions in block B_s.

10. $\{E_{s,j}, |E_{s,j}\rangle\}_j$ [Eq. (48)]: the eigenvalues and the eigenstates of h_s, respectively.

11. $h_{s,s+1}$ [Eq. (45)]: interactions between blocks B_s and B_{s+1}. The norm is bounded from above by g_0 as in (46).

12. $H_{\rm t}$ [Eq. (45)]: interaction-truncated Hamiltonian.

13. $\delta H_{\rm t}$ (Lemma 3): difference between $H_{\rm t}$ and H, namely $\delta H_{\rm t} := H - H_{\rm t}$.

14. $\{|0_{\rm t}\rangle, E_{{\rm t},0}, \Delta_{\rm t}\}$ (Lemmas 3 and 4): the ground state, the ground energy and the spectral gap of the Hamiltonian $H_{\rm t}$.

15. $K_{\rm t}$ (Proposition 2): approximate ground state projection (AGSP) for the ground state $|0_{\rm t}\rangle$.

16. $\{\tilde{h}_s, \tau_s, \tau\}$ [Eqs. (48) and (49)]: the block Hamiltonian in B_s with the energy cut-off up to $\tau_s = \tau + E_{s,0}$, where τ is a control parameter.

17. $\tilde{H}_{\rm t}$ [Eq. (50), Supplementary Figure 3]: effective Hamiltonian by using the multi-energy cut-off. The three parameters $\{q, l, \tau\}$ characterize $\tilde{H}_{\rm t}$.

18. $\{|\tilde{0}_{\rm t}\rangle, \tilde{E}_{{\rm t},0}, \tilde{\Delta}_{\rm t}\}$ (Theorem 5): the ground state, the ground energy and the spectral gap of the effective Hamiltonian $\tilde{H}_{\rm t}$.

19. $\{\lambda, \lambda'\}$ [Eq. (172)]: constants given in Theorem 5.

20. $K_m(x)$ [Eq. (162), Lemma 11]: approximate filter function which is utilized to construct the AGSP K.

21. $\Pi_I^{(s)}$, Π_I and $\tilde{\Pi}_I$ [Eqs. (231) and (234)]: the projection operators onto the eigenspaces of h_s, H and $\tilde{H}_{\rm t}$, respectively.

22. $\{E_\perp, \kappa\}$ [Eqs. (250) and (251)]: constants given in Lemma 15.

SUPPLEMENTARY REFERENCES

[1] B. Nachtergaele and R. Sims, Communications in Mathematical Physics **276**, 437 (2007).
[2] I. Arad, A. Kitaev, Z. Landau, and U. Vazirani, arXiv preprint arXiv:1301.1162 (2013), arXiv:1301.1162.
[3] F. Verstraete and J. I. Cirac, Phys. Rev. B **73**, 094423 (2006).
[4] W. W. Ho, I. Protopopov, and D. A. Abanin, Phys. Rev. Lett. **120**, 200601 (2018).
[5] I. Arad, Z. Landau, and U. Vazirani, Phys. Rev. B **85**, 195145 (2012).
[6] T. Kuwahara, I. Arad, L. Amico, and V. Vedral, Quantum Science and Technology **2**, 015005 (2017).
[7] I. Arad, T. Kuwahara, and Z. Landau, Journal of Statistical Mechanics: Theory and Experiment **2016**, 033301 (2016).
[8] M. B. Hastings, Journal of Statistical Mechanics: Theory and Experiment **2007**, P08024 (2007).
[9] C. Eckart and G. Young, Psychometrika **1**, 211 (1936).
[10] L. Biedenharn and J. Louck, Advances in Applied Mathematics **10**, 396 (1989).
[11] L. C. Biedenharn and J. D. Louck, Proceedings of the National Academy of Sciences of the United States of America **87**, 1441 (1990), 11607064[pmid].
[12] T. Kuwahara, New Journal of Physics **18**, 053034 (2016).
